# Fluorescent transmembrane anion transporters: shedding light on anionophoric activity in cells†
†Electronic supplementary information (ESI) available: Experimental synthesis details and characterisation of all compounds; ^1^H NMR titration binding studies, stacked spectra and fitted binding isotherms; UV-Vis and fluorescence spectroscopy studies data; various vesicle assays methods and Hill plots for ISE transport studies; details of MTT assay and annexin V assay, dose–response curves and IC_50_ values for cell viability studies; additional fluorescence microscopy images and co-staining experiments. CCDC 1424345. For ESI and crystallographic data in CIF or other electronic format see DOI: 10.1039/c6sc01643j
‡
‡The data underlying this paper has been made available online at http://dx.doi.org/10.5258/SOTON/392749 to comply with the EPSRC open data policy.


**DOI:** 10.1039/c6sc01643j

**Published:** 2016-04-26

**Authors:** Stuart N. Berry, Vanessa Soto-Cerrato, Ethan N. W. Howe, Harriet J. Clarke, Ishna Mistry, Ali Tavassoli, Young-Tae Chang, Ricardo Pérez-Tomás, Philip A. Gale

**Affiliations:** a Chemistry , University of Southampton , Southampton , SO17 1BJ , UK . Email: philip.gale@soton.ac.uk ; Tel: +44 (0)23 8059 3332; b Department of Pathology and Experimental Therapeutics , Cancer Cell Biology Research Group , University of Barcelona , Barcelona , Spain; c Singapore Bioimaging Consortium , Agency for Science , Technology and Research (A*STAR) , Singapore 138667 , Singapore; d Department of Chemistry and MedChem Program of Life Sciences Institute , National University of Singapore , Singapore 117543 , Singapore

## Abstract

A series of fluorescent anion transporters consisting of a urea or thiourea group linked to a naphthalimide fluorophore have been synthesised and their anion transport properties studied. The compounds possess similar anion transport properties to (thio)urea-based anionophores that have previously been reported. Fluorescence studies in cells show all anionophores cross the plasma membrane and localise within the interior of cells. The most lipophilic, aromatic substituted transporters localise homogeneously throughout the cell and are toxic towards cancer cells with the highly fluorinated compound **6** being the most effective. The least lipophilic, alkyl substituted transporters localise in specific vesicles and are non-toxic to cells. This work provides new insight to the actions of anionophores in cells and may be useful in the design of novel antineoplastic agents.

## Abstract 2

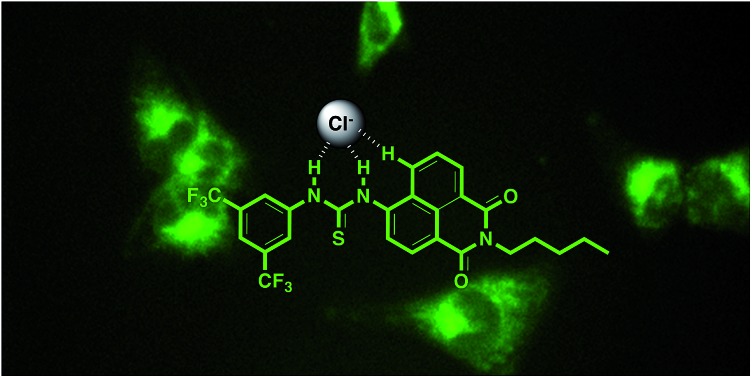
A series of fluorescent anion transporters have been synthesised and their anion transport properties and interactions with cancer cell lines studied.

## Introduction

The transport of anions across phospholipid bilayers is a key physiological process, important in regulating cellular pH, maintaining osmotic balance, and cellular signaling.^1^,^2^ In recent years, considerable research effort has been devoted to the design of small molecule synthetic compounds that mediate the transport of anions across lipid bilayer membranes.^3^–^5^ This research is driven in order to find potential future treatments for diseases that result from faulty anion transport in cells, known as ‘channelopathies’. As such anionophores may have potential to be developed as ‘channel replacement therapies’ restoring the permeability of cell membranes to anions.^6^ Additionally, the actions of anionophores have been linked to the disruption of pH gradients within acidic components of cells (such as lysosomes, endosomes and Golgi apparatus), leading to toxicity and subsequently these may have future applications as anticancer agents.^7^–^9^

Despite the recent effort devoted to the study of putative anionophores in phospholipid vesicles, our understanding of the actions of anion transporters in cells is still at a relatively early stage.^10^ Recently Gale, Sessler, Shin and co-workers have shown that anionophore mediated flux of chloride into cells is accompanied by sodium influx through endogenous sodium channels with the resulting higher salt levels resulting in apoptosis *via* caspase activation.^11^ A. P. Davis and co-workers have reported a highly preorganised bis-ureidodecalin that was shown to transport chloride across cell membranes in a chloride/iodide antiport process.^12^ A number of anionophores have shown cytotoxicity towards cancer cells such as the natural product prodigiosin.^13^,^14^ In many cases, toxicity has been attributed to perturbation of intracellular pH gradients between the cytosol and acidic organelles mediated by Cl^–^/H^+^ symport or functionally equivalent Cl^–^/OH^–^ antiport.^15^,^16^ An important class of synthetic anionophore are urea or thiourea-based anion receptors. These compounds have been shown to perturb pH gradients in cancer cells.^7^,^17^ Recently we have demonstrated that most simple anionophores are capable of dissipating pH gradients functioning either as weak acid protonophores^18^ or by transporting hydroxide.^19^ This may be one reason why many anionophores are promisingly toxic towards cancer cells.

In spite of these advances, there are still many questions about how anionophores act in cells. Primarily, in this study we wished to investigate where in the cell these compounds localise and how localisation may be related to potential cytotoxicity. We therefore synthesised fluorescent urea and thiourea anion transporters and used fluorescence microscopy to monitor the location of these compounds within cells. We chose the 1,8-naphthalimide fluorophore because its fluorescence properties have been well characterised,^20^,^21^ with this moiety having previously been used in anion sensors,^22^–^24^ anticancer agents^25^–^27^ (primarily *via* intercalation with DNA^28^,^29^) and in cellular imaging agents.^30^,^31^ In this study we directly appended the naphthalimide fluorophore to either anion-binding urea or thiourea groups *via* the 4-position of the naphthalimide (compounds **1–6**) to avoid potential photoinduced electron transfer (PET) quenching effects on fluorescence that may have occurred had the anion binding site been separated from the fluorophore by an aliphatic/aryl linker.^32^
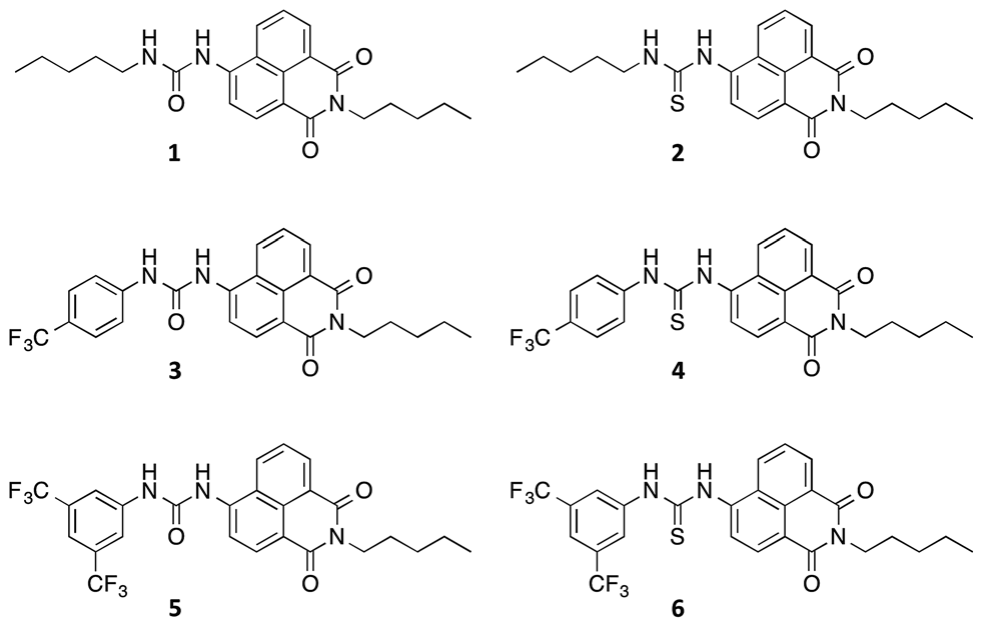


## Results and discussion

### Synthesis and characterisation

Compounds **1–6** were synthesised *via* four reaction steps. Briefly, condensation of commercially available 4-nitro-1,8-naphthalic anhydride with pentylamine gave the corresponding imide, followed by reduction of the nitro group to obtain the intermediate 4-amino-1,8-naphthalimide.^32^ Subsequently, the amine was converted to either isocyanate or isothiocyanate using triphosgene or thiophosgene respectively, and finally reaction with the relevant amine or aniline afforded compounds **1–6** in varying overall yields from 10–60%. Full synthetic details and characterisation data are provided in the ESI.†

**Fig. 1:**
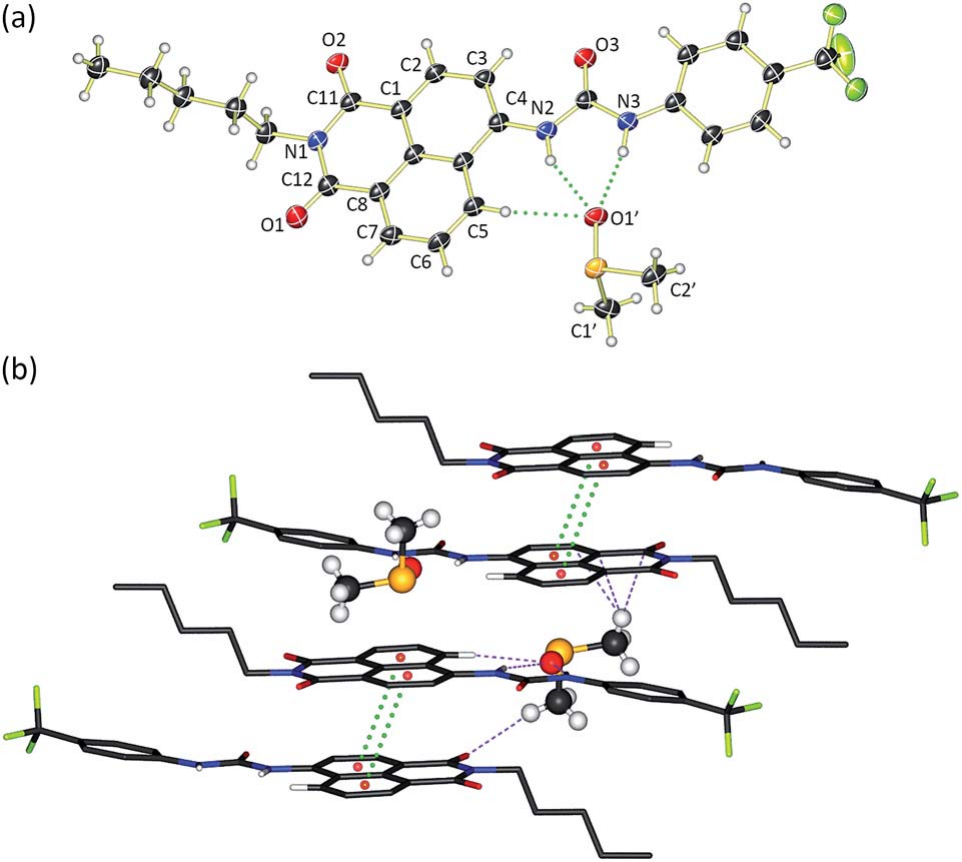
X-Ray crystal structure of **3**·DMSO. (a) ORTEP diagram showing 50% probability anisotropic displacement ellipsoids at 100 K. (b) Packing diagram viewed down the *b* axis showing π–π interactions (green dots) and intermolecular hydrogen-bonding interactions between bridging DMSO molecules (purple dots). All hydrogens in the packing diagram have been omitted for clarity, except those involved in hydrogen bonding interactions. Selected hydrogen bond distances (Å) and bond angles (°): C5···O1′ 3.406, N2···O1′ 2.920, N3···O1′ 2.807; C5–H···O1′ 171.3, N2–H···O1′ 151.5, N3–H···O1′ 166.6. π–π interactions 3.721 Å.

Crystals of compound **3** suitable for single crystal X-ray diffraction were obtained by slow evaporation of a DMSO/0.5% water solution of the compound in the presence of tetraethylammonium bicarbonate (15 molar equivalents). The structure was elucidated (Fig. 1a) and revealed that the compound crystallised as the DMSO solvate. Compound **3** was found to form a 1 : 1 solvate with DMSO with the solvent coordinated by three hydrogen bonds – two from the urea NHs (N···O′ distances of 2.920 and 2.807 Å and N–H···O′ bond angles of 151.5° and 166.6°) and a third aromatic CH hydrogen bond from the C5 position on the naphthalimide group (C···O′ distance of 3.406 Å, C–H···O′ angle of 171.3°). In addition, **3** was found to stack *via* π–π interactions between the naphthalimide groups (centroid–centroid distance of 3.721 Å) of adjacent molecules in an anti-parallel arrangement.^33^ As shown in Fig. 1b, the molecular crystal packing of compound **3** was found to alternate between anti-parallel π-stacked pairs and anti-parallel pairs linked by a solvent-bridge between a hydrogen bonded DMSO on one molecule and C2′H–π van der Waals interactions with the neighbouring molecule.

### Anion binding in solution

The affinity of compounds **1–6** for anions in solution was assessed using ^1^H NMR titration studies in DMSO-*d*_6_/0.5% water. Titrations were performed using biologically relevant anions added as either tetrabutylammonium (TBA) or tetraethylammonium (TEA) salts. A global fitting analysis^34^,^35^ using a 1 : 1 binding model was used to obtain association constants from the downfield shift of both urea/thiourea NH resonances and the aromatic CH proton resonance at the naphthalimide 5-position. Association constants for **1–6** binding to Cl^–^ are shown in Table 1 with stack plots and fitted curves for the ^1^H NMR titrations with all other anions provided in the ESI.†

**Table 1:** Summary of chloride association constants (*K*_a_) and anion transport parameters (EC_50_ and *n*) for receptors **1–6**. Calculated lipophilicity values (clog *P*) are also shown

	*K*_a_ Cl^–^^*a*^ (M^–1^)	EC_50_^*b*^ (mol%)	*n* ^*c*^	clog *P*^*d*^
**1**	31	—^*g*^	—^*g*^	4.70
**2**	41^*e*^	0.42	0.90	5.10
**3**	187	—^*g*^	—^*g*^	5.33
**4**	49	0.45	1.72	5.66
**5**	175	—^*g*^	—^*g*^	6.12
**6**	53^*f*^	1.35	1.13	6.44

^*a*^Association constant (M^–1^) calculated by fitting the change in chemical shifts upon addition of tetrabutylammonium (TBA) chloride of both urea/thiourea NH resonances and the naphthalimide CH resonance to a 1 : 1 global fitting binding model. ^1^H NMR (400 MHz) titrations were carried out in DMSO-*d*_6_/0.5% water at 298 K.

^*b*^Effective concentration – concentration in mol% of carrier with respect to lipid needed to obtain 50% chloride efflux at 270 s from POPC vesicles containing NaCl suspended in NaNO_3_.

^*c*^Hill coefficient from Hill analysis.

^*d*^Calculated log partition coefficient, average calculation from VCClabs.^36^

^*e*^Association constant calculated by fitting only naphthalimide NH proton signal due to overlapping peaks.

^*f*^Association constant calculated by fitting both urea NH proton signals only.

^*g*^Hill analysis not performed due to low transport activity and/or compound solubility.

Compounds **1–6** exhibited weak to moderate binding of chloride, with stronger complexes formed by compounds **3–6** that contain electron-withdrawing CF_3_-substituents than the alkyl analogues **1** and **2**. This is presumably due to the more acidic nature of the urea/thiourea NH protons in compounds **3–6**. Interestingly the aromatic proton resonance of naphthalimide 5-position also shifted significantly during the titrations, providing evidence that this hydrogen was also involved in the complexation of chloride. Hydrogen bond formation with DMSO was also observed from this CH in the X-ray crystal structure of **3** (see Fig. 1a).

With the exception of the alkyl-substituted naphthalimides **1** and **2**, it is evident that the urea-based receptors bind to chloride more strongly than their thiourea analogues. Generally thioureas are more acidic than ureas,^37^ however, we have previously observed stronger complex formation with ureas than thioureas.^17^,^38^,^39^

Titration studies with tetrabutylammonium nitrate were also conducted and negligible changes in chemical shifts of the (thio)urea NH groups observed, indicative of weak/no binding. Strong binding was observed for **1** with dihydrogen phosphate (*K*_a_ = 429 M^–1^) however, significant peak broadening was noted for the other compounds and therefore stability constants could not be obtained. In the titration studies with tetraethylammonium bicarbonate, significant peak broadening was observed for all compounds along with an associated colour change from yellow to pink/red. This could be indicative of either strong binding or deprotonation and was subsequently investigated further by UV-Vis titration.

### Spectroscopic investigations in DMSO and buffer at varying pH

**Fig. 2:**
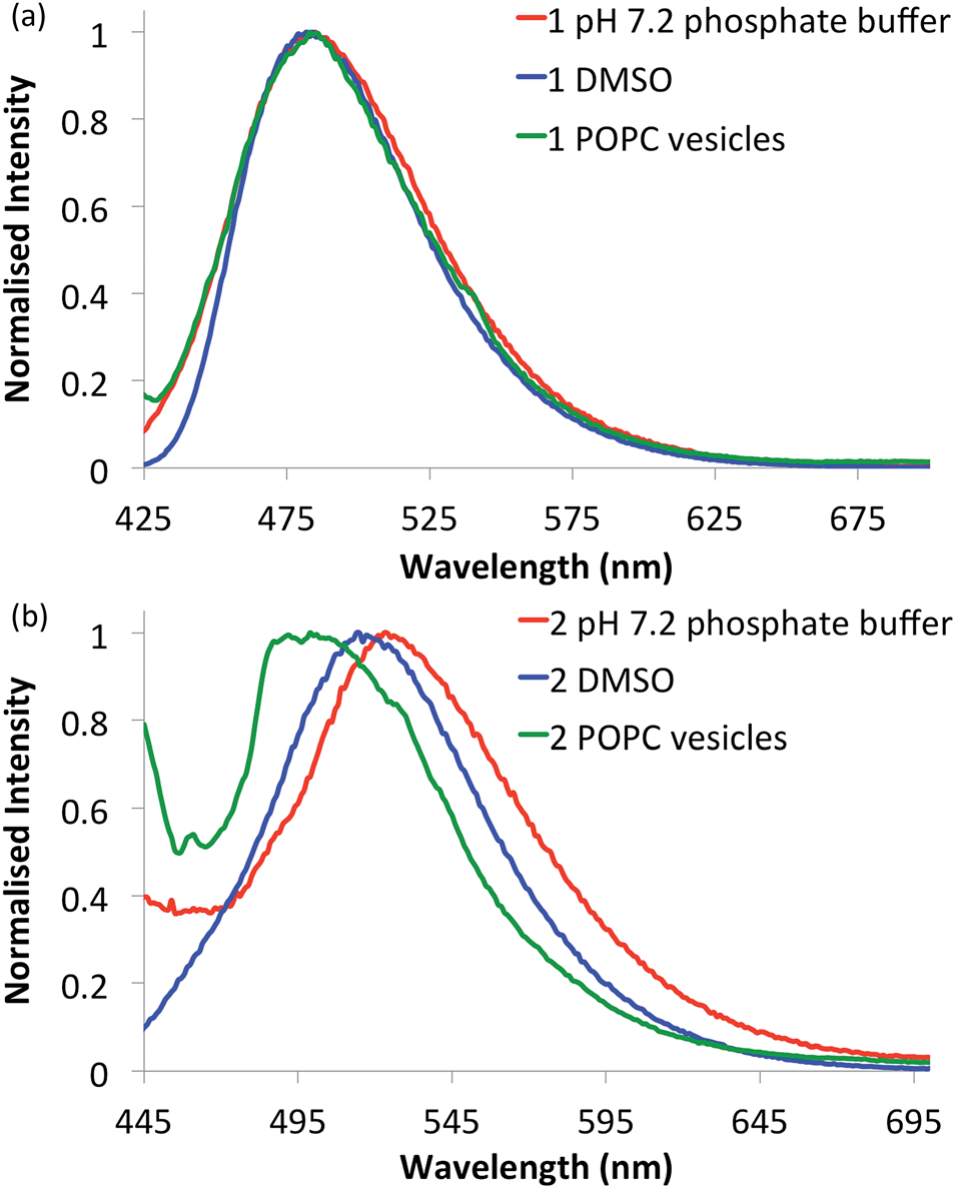
Normalised fluorescence emission spectra of selected naphthalimides. (a) Urea **1** (10 μM) and (b) thiourea **2** (10 μM) in various environments: 0.5% DMSO in pH 7.2 aqueous phosphate buffer (5 mM phosphate salts) (red curves), DMSO alone (blue curves) and in 1-palmitoyl-2-oleoyl-*sn*-glycero-3-phosphocholine (POPC) lipid vesicles (1 mM suspended in phosphate buffer) (green curves). See ESI† for respective emission spectra of all compounds.

Compounds **1–6** in DMSO exhibit broad absorption spectra with peak maxima ranging between 380 and 400 nm (see ESI Fig. S21–S26 for spectra†), and an emission spectrum with peaks ranging between 470 and 510 nm (see Fig. 2 and ESI Fig. S27–S32 for spectra†), due to internal charge transfer (ICT) of the excited state.

Due to the significant visible colour change observed upon addition of tetraethylammonium bicarbonate during the NMR binding studies, similar titration studies were conducted using UV-Vis spectroscopy. Initially, the absorption at 380–400 nm diminished progressively during the titration of bicarbonate and new absorbance bands emerged at 500–530 nm with distinct isosbestic points (see ESI Fig. S63–S68†). This is indicative of the formation of two species for all compounds and can be attributed to the deprotonation of the urea/thiourea NH as has been reported previously for naphthalimide–(thio)urea compounds.^40^–^42^ Subsequent titration with further aliquots of bicarbonate for thioureas **4** and **6** resulted in the formation of a third species, with a second isosbestic point coupled with a distinctive inflection of the absorbance band at 290 nm, indicative of deprotonation of the remaining thiourea NH (see ESI Fig. S66 and S68†). This is also supported by the NMR titration data in which the deprotonation process resulted in the downfield shift of the proximal aromatic CH resonance due to the through-space polarisation effect by the negatively charged deprotonated nitrogen, and the upfield shift of the distal aromatic protons from the inductive shielding effect (see ESI Fig. S39–S44†).^37^,^43^,^44^

In addition, to further investigate deprotonation of naphthalimides **1–6**, UV-Vis titrations were conducted in DMSO : water (9 : 1 ratio) containing 100 mM tetrabutylammonium hexafluorophosphate at a variety of different pH values. Under acidic conditions, no changes in the UV-Vis spectra were observed for all compounds. Furthermore all compounds were neutral at approximately pH 7 and therefore will be likely to bind/transport anions at physiological pH. The resulting acid/base equilibria from the absorption bands at ∼400 nm and ∼500 nm suggest approximate p*K*_a_ values are higher than physiological pH of 7.2 (see ESI Fig. S69–S74†).

With the intention of using **1–6** as a new class of fluorescent transmembrane anion transporters with potential applications in cells, the emission profiles were studied in various relevant environments. As shown in Fig. 2, there are no significant quenching effects of the emission band at ∼500 nm for urea **1** in DMSO and aqueous environments in the absence or presence of lipid vesicles; this was also observed with urea analogues **3** and **5**. The emission spectra of thiourea **2** are notably different, exhibiting a blue shift in the lipid environment and red shift in the more polar aqueous environment (also observed with thioureas **4** and **6**). Nonetheless, emission spectra are still obtainable in all environments tested rendering these compounds suitable for cellular applications. Furthermore, the emission spectra recorded in various acidic pH buffer solutions showed no significant quenching effects (see ESI Fig. S75–S80†).

### Transport studies

**Fig. 3:**
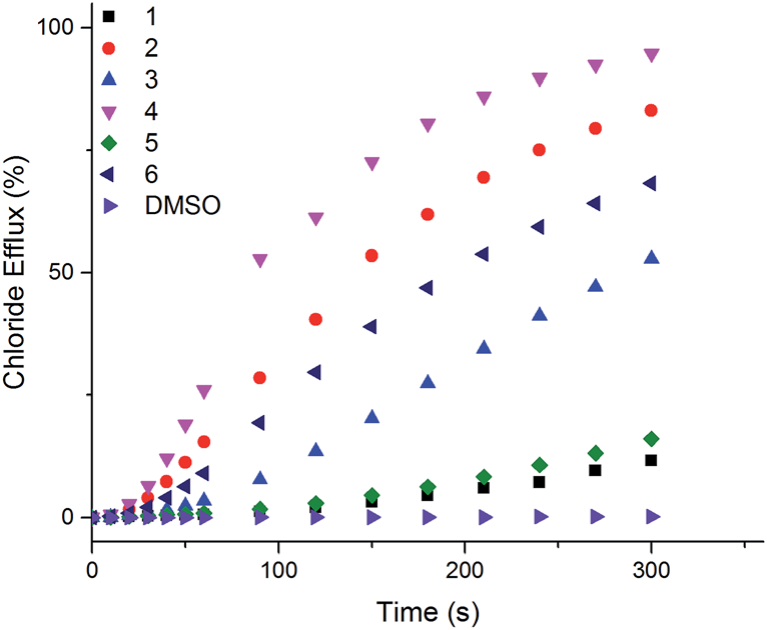
Chloride efflux as a function of time promoted by addition of 2 mol% (with respect to lipid concentration) of naphthalimides **1–6** from unilamellar POPC vesicles containing 489 mM NaCl buffered to pH 7.2 with 5 mM sodium phosphate salts suspended in 489 mM NaNO_3_ buffered to pH 7.2 with 5 mM sodium phosphate salts. The receptors were added as a DMSO solution. At 300 s (the end point of the experiment), the vesicles were lysed with Triton X-100 to calibrate the electrode to 100% chloride efflux. Each point is an average of 3 repeated runs. See ESI† for a version of this graph with error bars.

The ability of naphthalimides **1–6** to transport anions across phospholipid bilayers was determined using vesicle based techniques employing a chloride ion-selective electrode (ISE). In a typical assay, unilamellar 1-palmitoyl-2-oleoyl-*sn*-glycero-3-phosphocholine (POPC) vesicles (200 nm diameter) containing NaCl (489 mM, buffered to pH 7.2 with 5 mM phosphate salts) were suspended in an external solution of NaNO_3_ (489 mM, buffered to pH 7.2 with 5 mM phosphate salts). Anion transport was initiated by adding **1–6** at an appropriate concentration with respect to lipid concentration in 10 μL DMSO at *t* = 0 s and the resulting chloride efflux monitored by the ISE. At the end point of the experiment (300 s), the vesicles were lysed with Triton X-100 in order to calibrate the electrode to 100% chloride release. Fig. 3 shows the results of Cl^–^/NO_3_^–^ antiport experiments for **1–6**.

Thiourea-based naphthalimides **2**, **4** and **6** are significantly more active transporters than their urea analogues **1**, **3** and **5**. This trend has been observed in other systems, including simple aromatic thioureas^45^ and may be attributed to the reduced hydrophilicity of thioureas as compared to analogous ureas, thereby enhancing their ability to partition into the phospholipid bilayers. It is interesting to note here that urea–naphthalimides **1** and **5** are particularly inactive when compared to their respective thiourea analogues **2** and **6**, as such illustrating that binding affinity in this case, as in others, is not always an overriding determining factor of transport ability.^7^

In order to further quantify the transport properties and examine the transport mechanism of these naphthalimide transporters we performed Hill analyses^46^ on this series of compounds. Briefly, plots of receptor concentration *versus* chloride efflux 270 s after receptor addition were fitted to the Hill equation using Origin 9.1. Hill analysis provides two key parameters including the Hill coefficient (*n*), which is a measure of the stoichiometry of the complex formed in the lipid bilayer. Hill coefficients may therefore provide some insight into the transport mechanism (membrane spanning channel or mobile carrier). Hill analysis also provides a measure of transport efficiency (the EC_50_ value).^47^ This is the concentration of receptor needed to induce 50% chloride efflux from the vesicles during the time course of the experiment. Table 1 shows the results of Hill analyses performed for transporters **1–6**.

Hill analysis could not be accurately performed for the three urea-based compounds due to low transport activity/solubility; at high receptor loadings, a precipitate was observed when these compounds were added to the vesicle solution and therefore accurate transport measurements were not possible. Naphthalimides **2** and **4** are the more active transmembrane anion transporters whereas **6** is slightly less active. The difference in activity is possibly due to the lipophilicity of these compounds as all three receptors have a similar affinity for chloride. Table 1 also shows calculated octanol : water partition coefficient (clog *P*) values for **1–6**. clog *P* values are a calculated measure of lipophilicity and two recent studies have shown that the correct balance of lipophilicity about the anion binding site^48^ and a suitable overall lipophilicity range^49^ are important in designing efficient anion transporters. It is possible that the energy barrier for **6** to diffuse through the polar head group region of the lipid bilayer is higher than that for the other compounds, and therefore the rate of transport for this compound is slightly lower.

Hill coefficients are related to the stoichiometry of the transported species.^50^ Here, Hill coefficients between 0.9 and 1.7 are evidence to suggest that the most likely transport mechanism facilitated by these compounds is a 1 : 1 mobile carrier process. This is supported by the anion complexation data and transport studies on other structurally similar ureas and thioureas.^17^,^51^

To rule out the possibility that M^+^/Cl^–^ symport was occurring, cation exchange experiments were performed in which the standard Cl^–^/NO_3_^–^ assay was repeated with POPC vesicles encapsulating CsCl instead of NaCl were suspended in NaNO_3_ external solution. The results showed negligible differences between transport from NaCl vesicles and CsCl vesicles, (see ESI Fig. S95†). Further, when we replaced the external nitrate solution for a dianionic sulphate solution (see ESI Fig. S94†) negligible transport was observed, suggesting M^+^/Cl^–^ symport is not occurring and the observed efflux is Cl^–^/NO_3_^–^ antiport.

**Fig. 4:**
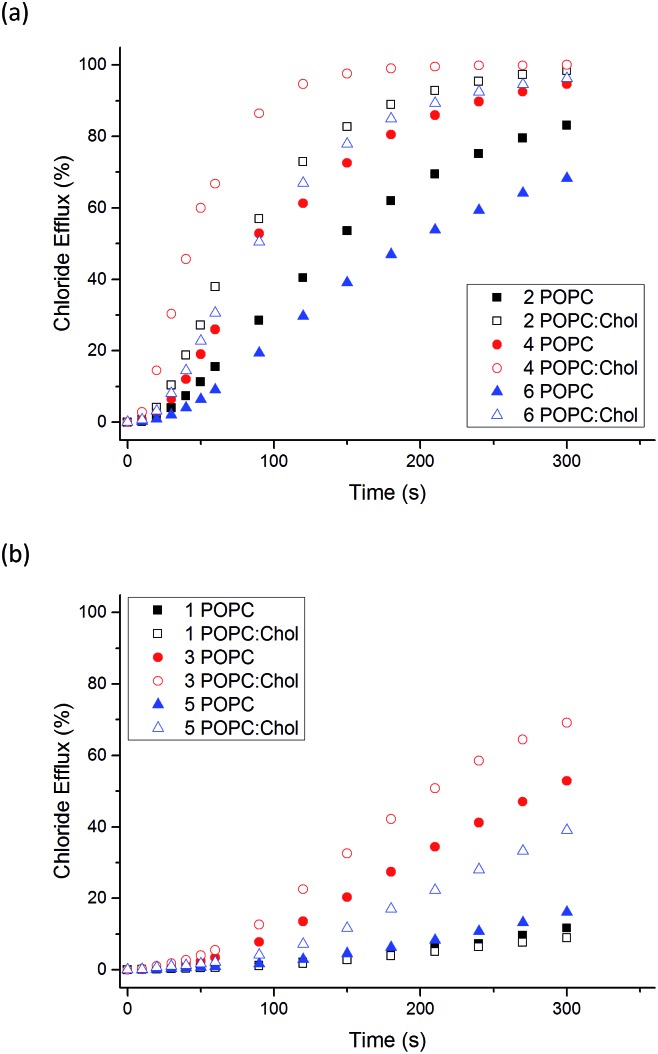
Comparison of Cl^–^/NO_3_^–^ antiport initiated by **1–6** (2 mol% receptor loading with respect to total lipid concentration) from vesicles composed of POPC (filled symbols) or POPC : cholesterol 7 : 3 (POPC : Chol), (hollow symbols). Vesicles contained 489 mM NaCl buffered to pH 7.2 with 5 mM sodium phosphate salts and were suspended in 489 mM NaNO_3_ buffered to pH 7.2 with 5 mM sodium phosphate salts. The receptors were added as a DMSO solution. At 300 s (the end point of the experiment), the vesicles were lysed with Triton X-100 to calibrate the electrode to 100% chloride efflux. Each point is an average of 3 repeated runs. Comparison graphs show (a) thioureas **2**, **4**, **6** and (b) ureas **1**, **3**, **5**.

We also examined the transport ability of **1–6** in vesicles composed of POPC : cholesterol (7 : 3 molar ratio). Cholesterol is found in high concentrations in living cells (typically 20–30%) and its ordering effects on lipid bilayer membranes have been described previously.^52^ Cholesterol doped vesicles have been extensively used in transport studies to examine how transporters move through a more viscous membrane and furthermore, these vesicles can provide a closer model of a cell membrane and therefore are of interest. Fig. 4 shows a comparison of transport efficiency between vesicles composed entirely of POPC and POPC : cholesterol (7 : 3 molar ratio).

For all naphthalimides except the least active compound **1**, enhanced transport efficiency was observed in POPC : cholesterol vesicles than in POPC vesicles. This suggests that the rate-determining step of transport is not diffusion of the carrier or complex through the hydrophobic tail region of the bilayer leaflet. Pleasingly it shows that transport is more efficient in vesicles that more closely resemble cells, in particular **3** and **5** facilitate moderate Cl^–^ efflux under these conditions. Increased transport rates in POPC : cholesterol vesicles as opposed to pure POPC vesicles has been reported previously.^45^,^53^

### Studies of fluorescent anion transporters with cells

There is growing evidence to suggest that modulation of cellular ion transport may be used to trigger apoptosis.^54^,^55^ Because of this, the transport of ions into cells has been previously proposed as a strategy for potential treatment of cancer. The cytotoxicity of transporters **1–6** towards two cancerous cell lines (human lung carcinoma, A549 and human breast adenocarcinoma, MCF-7) were investigated by MTT cell viability assay (Table 2).

**Table 2:** IC_50_ values of compounds **1–6** on human lung carcinoma (A549) and human breast adenocarcinoma (MCF-7) cell lines

Compound	IC_50_ (μM) A549	IC_50_ (μM) MCF-7
**1**	>50	>50
**2**	>50	>50
**3**	22.6 ± 7.5	40.3 ± 3.0
**4**	17.1 ± 3.0	12.7 ± 2.1
**5**	7.7 ± 4.1	31.2 ± 3.2
**6**	7.6 ± 1.1	12.3 ± 1.5

Interestingly, the alkyl-substituted transporters **1** and **2** were found to be non-toxic towards both cancer cell lines. The remaining transporters were all potent to both cell lines to varying degrees of cytotoxicity. In MCF-7 cells, the transport active thioureas **4** and **6** are more toxic than their urea counterparts (**3** and **5** respectively), however, this trend is less defined in the A549 cell line where the most lipophilic compounds **5** and **6** have similar toxicity values. All toxic compounds were found to be more toxic against A549 cells than MCF-7 cells and in A549 cells, toxicity increased with lipophilicity.

In order to investigate the mechanism of toxicity, we performed an annexin-V assay with the most potent compound **6** to evaluate if this compound could induce apoptosis in a similar fashion to previously reported ionophores.^11^ This assay showed that at high doses, **6** induced late apoptosis in cells (see ESI Fig. S98† for details and brief discussion).

**Fig. 5:**
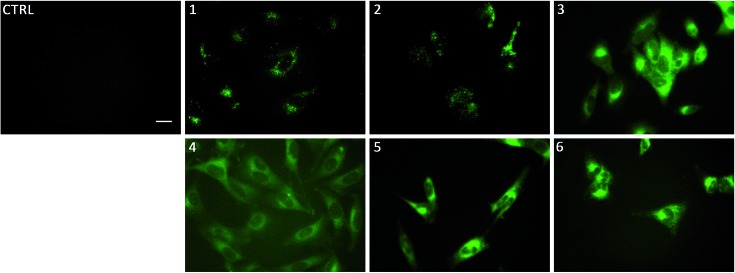
Fluorescence micrographs of A549 cells after incubation with **1–6** for 24 hours. **1**, **3** and **5** were incubated at 1 μM, **2**, **4**, and **6** at 5 μM. Control cells were incubated with 0.5% DMSO/DMEM solution (the carrier used in these experiments). Scale bar = 20 μm. DMEM: Dulbecco's Modified Eagle Medium.

To gain insight on the localisation of transporters **1–6** in cells, fluorescence micrographs were collected after treatment of lung cancer cells (A549) with **1–6** for 24 hours (Fig. 5). It was apparent that there were two distinct localisation pathways. The aromatic substituted transporters (**3–6**) all appear to localise homogenously throughout the cytoplasm whereas the less lipophilic alkyl substituted transporters (**1–2**) appear to be localised in more specific structures within the cell. To the best of our knowledge, this is the first example of a transmembrane anion transporter being shown to localise in live cells.

To further investigate how transporters **1–6** were interacting with the biological membrane, we performed ‘washing out’ experiments whereby we examined how easy it was to wash these compounds out of the cells. These experiments were performed as follows: A549 cells were treated with **1–6** for 1 hour, then subsequently washed twice with PBS (phosphate buffered saline), the media replaced and fluorescence micrographs taken. Following this, the cells were placed back in the incubator at 37 °C for 1 hour, washed twice with PBS and imaged again (2nd wash). The cells were then incubated again for a final hour, washed twice and imaged a final time (3rd wash). All images were corrected for contrast to the brightest image (1st wash) so as to be comparable and are provided in the ESI.† The ‘washing out’ experiments showed that these compounds were easily washed out of the cells (after first or second wash), demonstrating the reversible shuttling of the transporter over the cells plasma membrane down concentration gradients, much in the same way we presume these compounds cross POPC bilayers in the transport studies.

We initially hypothesised these compounds may localise within the cell membrane as they were designed as membrane transport agents however, it is clear that all compounds pass through the plasma membrane and fluoresce within the cells cytoplasmic contents. We have previously shown anion transporters are able to deacidify lysosomes, which then in turn induces apoptosis.^7^,^8^,^17^ As such, it seems logical that these compounds localise within the interior of the cell and not within the plasma membrane in order to induce toxicity *via* perturbation of pH gradients of acidic organelles.

**Fig. 6:**
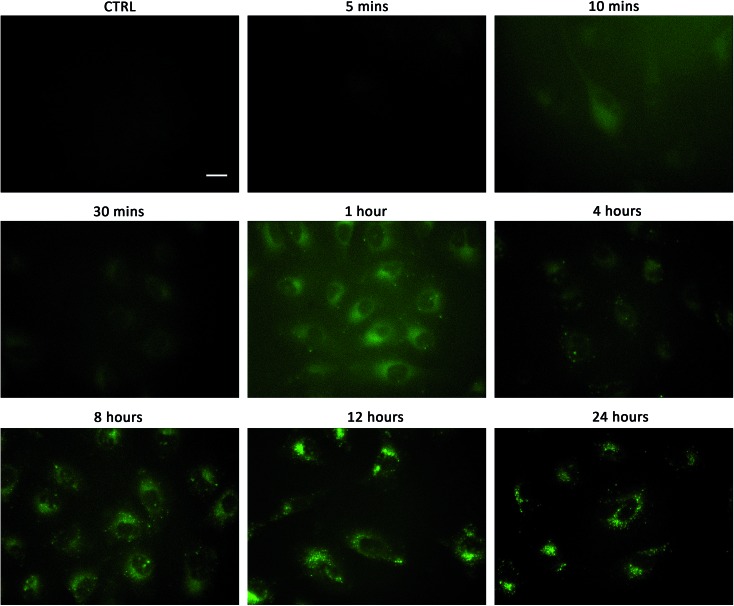
Fluorescent micrographs showing the localisation of compound **1** over time in A549 cells. **1** (1 μM) was incubated with A549 cells for the stated time, washed twice with PBS before images taken. Control cells were incubated with 0.5% DMSO/DMEM solution (the carrier used in these experiments). Scale bar = 20 μm.

Linking cellular localisation to toxicity, it is significant that **1** and **2** are essentially non-toxic to the cell lines studied. This may be because they are localised over specific intracellular compartments and are unable to act over the whole cell unlike the more toxic **3–6**, which are distributed homogeneously within each cell. In order to investigate this further, we performed imaging studies with **1–6** at different incubation times ranging from 5 minutes to 24 hours (Fig. 6 and ESI Fig. S99–104†). These imaging studies showed that the aromatic naphthalimides (**3–6**) localised in the cells after approximately 1 hour and there was subsequently no change in fluorescent signal from this time point onwards. It should be noted that the time scale for localisation in cells is longer than in our liposome transport studies, possibly due to differences in membrane composition compared to model POPC vesicles.

Interestingly, the alkyl substituted **1** and **2** interacted with cells over time differently than the remaining transporters **3–6** (Fig. 6 for **1**, ESI Fig. S100† for **2**). These compounds appeared to initially localise homogenously throughout the cytoplasm (after 1 hour) and then, subsequently, localisation was more evident in specific spherical organelles which progressed over the incubation time. Therefore, there may be some kind of cellular uptake mechanism whereby the cells are activated over time to expel these compounds *via* exocytosis. It is possible then, that this is the reason why these compounds are non-toxic to the cells despite **2** being an active ionophore from the POPC liposome assay. Co-staining experiments with LysoTracker Red and MitoTracker Deep Red suggested that **1** and **2** do not localise in lysosomes or mitochondria (see ESI Fig. S111 and 112†).

It may be significant that the most lipophilic compounds show the highest potency towards cancer cells whereas the least lipophilic transporters are non-toxic and show specific localisation patterns. More lipophilic transporters are more likely to interact within intracellular bilayer membranes and are presumably therefore better suited to perturb ionic gradients within lipophilic components of the cell. We have previously demonstrated highly lipophilic transporters being highly toxic towards cancer cells.^8^,^56^ Although transport efficiency in vesicles may be most effective within a specific lipophilicity range,^49^ if ionophores are to be optimised for future use as antineoplastic agents, it is critical that these compounds are interacting within the interior of the cell and (hence are active in mediating ion transport) as in this case cytotoxicity appears to increase with lipophilicity.

## Conclusions

We have synthesised a series of fluorescent urea and thiourea naphthalimide-based transmembrane anion transporters. The compounds show similar anion complexation and transport properties to previous generations of (thio)urea based transporters with the thiourea compounds proving to be more effective transporters than their urea analogues. The aromatic substituted compounds all induce cytotoxicity in cancer cell lines, with **6** inducing apoptosis in A549 cells, again in line with previous results. Most significantly, we have shown for the first time that these transporters localise within the cytoplasm of cells and not solely in the plasma membrane. Furthermore, we have shown two distinct localisation modes within cells; whereas the aromatic substituted transporters localise within the cytoplasm, the less lipophilic alkyl substituted transporters are localised in specific vesicles over time and notably, these compounds are not toxic towards cancer cells. This work therefore provides new insights into how certain ionophores interact with cells and may aid the design of future ionophore antineoplastic agents; it suggests the toxic effects are due to changes in ionic/pH gradients across intracellular membranes and not the plasma membrane.

## Supplementary Material

Supplementary information

Crystal structure data
