# Supplementary material for: Fluorescent transmembrane anion transporters: shedding light on anionophoric activity in cells
Source: Chem Sci. 2016 Apr 26;7(8):5069–77. doi: 10.1039/c6sc01643j (PMC6018715; doi:10.1039/c6sc01643j)
Supplement: Supplementary file 1 [file SC-007-C6SC01643J-s001.pdf]

Electronic Supplementary Material (ESI) for Chemical Science.
This journal is © The Royal Society of Chemistry 2016

Supporting Information

Fluorescent transmembrane anion transporters: shedding light on anionophoric activity in cells

Stuart N. Berry,[a,c] Vanessa Soto-Cerrato,[b] Ethan N. W. Howe,[a] Harriet J. Clarke,[a] Ishna Mistry,[a] Ali Tavassoli,[a] Ricardo Pérez-Tomás,[b] Young-Tae Chang,[c,d] and Philip A. Gale[a*]

[a] Chemistry, University of Southampton, Southampton, SO17 1BJ, UK; Tel: +44 (0)23 8059 3332;
[b] Department of Pathology and Experimental Therapeutics, Cancer Cell Biology Research Group, University of Barcelona, Barcelona, Spain;
[c] Singapore Bioimaging Consortium, Agency for Science, Technology and Research (A*STAR), Singapore 138667, Singapore
[d] Department of Chemistry and MedChem Program of Life Sciences Institute, National University of Singapore, Singapore 117543, Singapore

E-mail: philip.gale@soton.ac.uk

Table of Contents

Table of Contents 2

General Remarks 3

1 – Overview of Compounds 4

2 – Synthesis 5

3 – Characterisation 13

3.1 NMR Spectra 13

3.2 High Resolution Mass Spectrometry (HRMS) 16

3.3 UV-Vis Spectra 21

3.4 Fluorescence Spectra 24

3.5 Single Crystal X-Ray Diffraction 26

4 – NMR Binding Studies 26

4.1 Overview and Procedure 26

4.2 Interaction with TBACl 28

4.3 Interaction with $TEAHCO_3$ 34

4.4 Interaction with TBAOH 36

4.5 Interaction with $TBAH_2PO_4$ 39

4.6 Interaction with $TBANO_3$ 42

5 – UV-Vis Studies 45

5.1 UV-Vis Studies with $TEAHCO_3$ 45

5.2 UV-Vis studies in varying pH 50

6 – Additional Fluorescence Studies 57

6.1 Fluorescence spectra in varying pH 57

6.2 Fluorescence spectra in POPC Vesicles 61

7 – Transport Studies 64

7.1 Overview and Procedure 64

7.2 Cl^-/NO_3^- Transport Studies 64

7.3 Hill Plots and Analysis 67

7.4 Cl^-/HCO_3^- Transport Studies 69

7.5 Evidence for Antiport 70

8 – In Vivo Assays 71

8.1 Cell viability assay (MTT) 71

8.2 Annexin-V Assay 73

8.3 Fluorescence Imaging of Transporters in Cells 75

8.3.1 Time dependent cellular localisation studies 76

8.3.2 'Washing-Out' Imaging Experiments 81
8.3.3 Co-Localisation Experiments 84
10 – References 85

General Remarks

All starting materials and solvents were purchased from commercial sources and used without further purification unless stated otherwise. All NMR data were recorded on Bruker AVII400 or Bruker AVIIHD400 FT-NMR spectrometers and references to the indicated solvent at 298 K. Chemical shifts reported on the delta scale and abbreviations used for spink multiplicity of peaks include: s = singlet, d = doublet, t = triplet, q = quartet, quin = quintet, m = multiplet, br = broad.

All receptor samples were submitted to the University of Southampton Institute for Applied Mass Spectrometry (**UoSIAMS**) for HRMS (ES). Samples were analysed using a MaXis (Bruker Daltonics, Bremen, Germany) mass spectrometer equipped with a Time of Flight (TOF) analyser. The sample was introduced via a Diones Ultimate 3000 autosampler and uHPLC pump. Gradient 20% acetonitrile (0.2% formic acid) to 100% acetonitrile (0.2% formic acid) in five minutes at a flow rate of 0.6 mL/min. Column, Acquity UPLC BEH C18 (waters) 1.7 micron 50 x 2.1mm high. High resolution spectra was recorded using positive/negative electrospray ionization. Melting point (Mp) analyses were carried out using a Barnstead Electrothermal IA9100 melting point machine.

Fluorescence spectra were recorded on a Cary Eclipse fluorescence spectrophotometer at 298 K and UV-Vis spectra were recorded on a Cary 100 UV-Vis spectrophotometer.

1 – Overview of Compounds

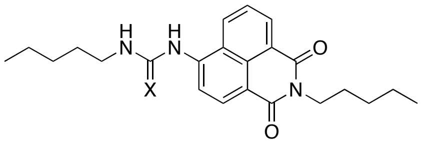

1 - X = O
2 - X = S

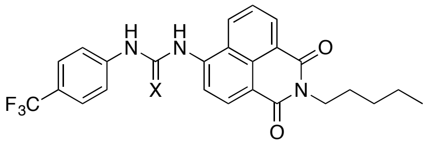

3 - X = O
4 - X = S

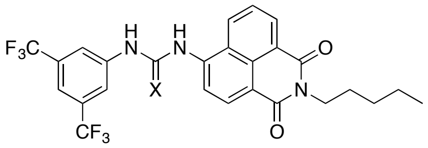

5 - X = O
6 - X = S

2 – Synthesis

Synthesis of 7 – (6-nitro-2-pentyl-1H-benzo[de]isoquinoline-1,3(2H)-dione)

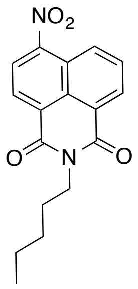

7, 90%

The synthesis of **7** was carried out using an adapted method form Gunnlaugsson *et al*.[1] The commercially available 4-nitro-1,8-naphthalic anhydride (1 g, 4.1 mmol) was suspended in ethanol in an anhydrous environment. Added dropwise to this was pentylamine (0.5 mL, 4.2 mmol) and the reaction was heated at reflux for 15 hours during which time dissolution occurred. The solvent was then removed *in vacuo* and the resultant solid re-dissolved in dichloromethane (125 ml). This was washed with saturated $NaHCO_3$ solution (2 x 50 mL), then H_2O (2 x 50 mL) and finally brine (50 ml). After drying over $MgSO_4$, the solvent was removed *in vacuo* to give **7** in 90% yield (1.20 g, 3.8 mmol).

^{1}H NMR (400 MHz, $CDCl_3$) δ: 8.82 (d, 1H, aromatic H, J = 8.68 Hz), 8.70 (d, 1H, aromatic H, J = 10.88 Hz), 8.68 (d, 1H, aromatic H, J = 8.05 Hz), 8.39 (d, 1H, aromatic H, J = 7.92 Hz), 7.98 (dd, 1H, aromatic H, J_1 = 8.68 Hz, J_2 = 7.32 Hz), 4.18 (t, 2H, aliphatic CH_2, J = 7.56 Hz), 1.74 (m, 2H, aliphatic CH_2), 1.41 (m, 4H, aliphatic CH_2 x 2), 0.92 (t, 3H, aliphatic CH_3, J = 6.96 Hz). ^{13}C {^{1}H} NMR (101 MHz, $CDCl_3$) δ: 163.2, 162.4, 149.5, 132.4, 129.8, 129.7, 129.2, 129.1, 123.9, 123.6, 123.1, 40.8, 29.2, 27.6, 22.4, 13.9. LRMS (ESI$^+$): *m/z* = 313.1 $[M+H]^+$. HRMS (ESI$^+$) for $C_{17}H_{16}N_2O_4$ $[M+H]^+$: *m/z* = 313.1183 (calcd), 313.1178 (found). Mp: 86-90 °C

Synthesis of 8 – (6-amino-2-pentyl-1H-benzo[de]isoquinoline-1,3(2H)-dione)

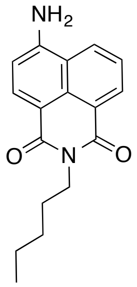

8, 100%

The synthesis of **8** was carried out using an adapted method from Gunnlaugsson *et al.*[1] Compound **7** (1.20 g, 3.8 mmol) was dissolved in ethanol (250 mL) and the solution de-gassed under nitrogen. Palladium (10% wt) on activated carbon (50 mg) was added and the solution stirred under hydrogen atmosphere for 15 hours at room temperature and pressure. The solution was then filtered through a celite pad and the solvent removed *in vacuo* affording compound **8** in quantitative yield.

^{1}H NMR (400 MHz, $CDCl_3$) δ: 8.60 (d, 1H, aromatic H, J = 7.21 Hz), 8.42 (d, 1H, aromatic H, J = 8.19 Hz), 8.11 (d, 1H, aromatic H, J = 8.31 Hz), 7.66 (t, 1H, aromatic H, J = 7.89 Hz), 6.89 (d, 1H, aromatic H, J = 8.19 Hz), 4.97 (br s, 2H, amine NH_2), 4.15 (t, 2H aliphatic CH_2, J = 7.60 Hz), 1.74 (m, 2H, aliphatic CH_2), 1.40 (m, 4H, aliphatic CH_2 x 2), 0.91 (t, 3H, aliphatic CH_3, J = 6.96 Hz). ^{13}C{^{1}H} NMR (101 MHz, $CDCl_3$) δ: 164.6, 162.0, 148.9, 133.7, 131.4, 129.8, 126.7, 125.0, 123.2, 120.1, 112.4, 109.6, 40.2, 29.3, 27.5, 22.3, 14.0. LRMS (ESI$^+$): *m/z* = 283.2 $[M+H]^+$. HRMS (ESI$^+$) for $C_{17}H_{18}N_2O_2$ $[M+H]^+$: *m/z* = 283.1441 (calcd), 283.1439 (found). 138-144 °C

Synthesis of 1 – (1-(1,3-dioxo-2-pentyl-2,3-dihydro-1H-benzo[de]isoquinolin-6-yl)-3-pentylurea)

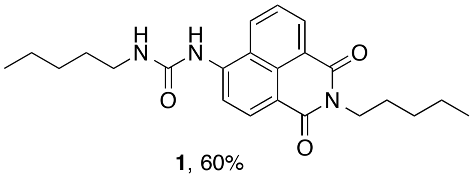

1, 60%

Compound **8** (170 mg, 0.5 mmol, 1eq) was dissolved in dichloromethane (50 mL). Saturated $NaHCO_3$ aqueous solution (40 mL) was added to this solution and the biphasic mixture stirred. Triphosgene (148 mg, 0.5 mmol 3 eq) was dissolved in dichloromethane (5 mL) and added dropwise to the stirring solution and the reaction stirred at room temperature for 15 hours. The biphasic mixture was separated and the organic phase was washed with H_2O (2 x 50 mL) and dried with $MgSO_4$. The organic solution was separated from the $MgSO_4$ and pentylamine (0.1 mL, 0.65 mmol, 1.3 eq) was added dropwise to the stirred solution and the reaction stirred at room temperature for two days. The resultant precipitate was filtered and washed with cold dichloromethane (2 x 50 mL) before drying under vacuum gave **1** as an off-white solid in 60% yield (117 mg, 0.296 mmol).

^{1}H NMR (400 MHz, DMSO-d_6) δ: 9.19 (s, 1H, urea NH), 8.59 (d, 1H, aromatic CH, J = 8.44 Hz), 8.53 (m, 2H, aromatic CH x 2), 8.40 (d, 1H, aromatic CH, J = 8.40 Hz), 7.88 (t, 1H, aromatic CH, J = 7.44 Hz), 6.92 (t, 1H, aliphatic urea NH, J = 5.4 Hz), 4.02 (t, 2H, aliphatic CH_2, J = 7.32), 3.18 (m, 2H, aliphatic CH_2), 1.64 (m, 2H, aliphatic CH_2), 1.52 (m, 2H, aliphatic CH_2), 1.33 (m, 2H, aliphatic CH_2 x 4), 0.89 (m, 2H, aliphatic CH_3 x 2). ^{13}C{^{1}H} NMR (101 MHz, DMSO-d_6) δ: 163.9, 163.3, 154.8, 143.0, 132.6, 131.1, 128.9, 128.1, 126.3, 122.8, 122.2, 114.6, 114.3, 29.6, 29.2, 29.1, 27.7, 22.3, 14.4, 14.3. LRMS ESI$^+$ (m/z): 396.0 [M+H]$^+$. HRMS (ESI$^+$) for $C_{23}H_{30}N_3O_3$ [M+H]$^+$: *m/z* = 396.2282 (calcd), 396.2289 (found). Mp: 220-223 °C

Synthesis of 2 – (1-(1,3-dioxo-2-pentyl-2,3-dihydro-1H-benzo[de]isoquinolin-6-yl)-3-pentylthiourea)

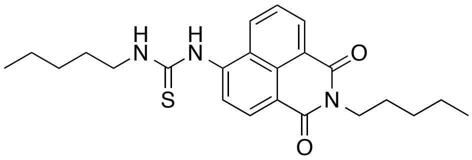

2, 58%

The intermediate isothiocyanate was synthesised from an adapted method of Anzenbacher Jr *et al.*[2] Compound **8** (0.5 g, 1.77 mmol, 1 equiv) was dissolved in acetone (70 mL). Thiophosgene (0.28 mL, 3.72 mmol, 2.1 equiv) was dissolved in acetone (25 mL) and added dropwise to the amine solution at 0 °C. The solution was allowed to warm to room temperature and stirred at room temperature for 2 days. The solvent was removed under vacuum and the resultant crude isothiocyanate (200 mg, 0.62 mmol, 1 equiv) was dissolved in $CHCl_3$ (10 mL). Pentylamine (0.678 mL, 0.68 mmol, 1.1 equiv) was dissolved in $CHCl_3$ (5 mL) and added dropwise to the stirring isothiocyanate solution. The solution was stirred at reflux overnight and upon cooling the solvent removed. The product was re-crystallised from dichloromethane/hexane and the resultant filtrate placed down an SCX-2 column (1:9 ethyl acetate/hexane eluent) giving **2** as a yellow solid in 58% yield (145 mg, 0.35 mmol).

^{1}H NMR (400 MHz, DMSO-d_6) δ: 10.04 (s, 1H, thiourea NH), 8.51 (d, 1H, aromatic CH, J = 7.21 Hz), 8.46 (d, 1H, aromatic CH, J = 8.07 Hz), 8.41 (d, 1H, aromatic CH, J = 8.31 Hz), 8.20 (br t, 1H, thiourea NH), 8.09 (br d, 1H, aromatic CH, J = 7.34 Hz), 7.87 (br t, 1H, aromatic CH, J = 7.76), 4.04 (br t, 2H, aliphatic CH_2, J = 7.21), 3.49 (br d, 2H, aliphatic CH_2, J = 5.62), 1.63 (m, 2H, aliphatic CH_2), 1.59 (m, 2H, aliphatic CH_2), 1.32 (br s, 8H, aliphatic CH_2 x 4), 0.88 (m, 6H, aliphatic CH_3 x 2). ^{13}C{^{1}H} NMR (101 MHz, DMSO-d_6) δ: 181.3, 163.4, 162.9, 141.7, 131.2, 130.9, 129.3, 128.7, 126.8, 122.9, 122.3, 118.2, 44.2, 28.6, 28.2, 27.2, 21.9, 21.8, 13.9, 13.8. LRMS ESI$^+$ (m/z): 412.1 $[M+H]^+$. HRMS (ESI$^+$) for $C_{23}H_{30}N_3O_2S$ $[M+H]^+$: *m/z* = 412.2053 (calcd), 412.2051 (found). Mp: 151-154 °C

Synthesis of 3 - (1-(1,3-dioxo-2-pentyl-2,3-dihydro-1H-benzo[de]isoquinolin-6-yl)-3-(4-(trifluoromethyl)phenyl)urea)

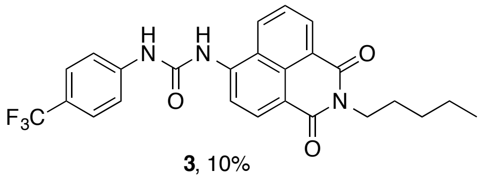

3, 10%

Compound **8** (400 mg, 1.46 mmol, 1eq) was dissolved in dichloromethane (100 mL). Saturated $NaHCO_3$ aqueous solution (100 mL) was added to this and the biphasic mixture stirred. Triphosgene (424 mg, 1.46 mmol 3 eq) was dissolved in dichloromethane (15 mL) and added dropwise to the stirring solution and the reaction stirred at room temperature for 15 hours. The biphasic mixture was separated and the organic phase was washed with H_2O (2x50 mL) and dried with $MgSO_4$. The organic solution was separated from the $MgSO_4$ and concentrated to *ca.* 50 mL. 4-(Trifluoromethyl)aniline (0.18 mL, 1.42 mmol, 1 eq) was added dropwise to the stirred solution and the reaction stirred at room temperature for two days. The resultant precipitate was filtered and washed with cold dichloromethane (2x50 mL) before re-crystallisation from methanol produced **3** as an off-white solid in 10% yield (50 mgs, 0.11 mmol).

^{1}H NMR (400 MHz, DMSO-d_6) δ: 9.67 (s, 1H, thiourea NH), 9.51 (s, 1H, thiourea NH), 8.61 (d, 1H, aromatic CH, J = 8.65 Hz), 8.51 (m, 3H, aromatic CH x 3), 7.95 (t, 1H, J = 8.28 Hz), 7.72 (m, 4H, aromatic CH x 4), 4.03 (br t, 2H, aliphatic CH_2 J = 7.36 Hz), 1.65 (br t, 2H, aliphatic CH_2), 1.33 (m, 4H, aliphatic CH_2 x 2), 0.87 (br t, 3H, aliphatic CH_3, J = 6.96 Hz). ^{13}C (126 MHz, DMSO-d_6) δ: 163.5, 162.9, 151.9, 142.8, 141.2, 132.2, 130.8, 128.4, 126.4, 126.3, 126.2, 122.6, 122.5 118.2, 115.7, 115.5, 28.7, 27.2, 21.8, 13.8. LRMS ESI$^+$ (m/z): 470.32 [M+H]$^+$. HRMS (ESI$^+$) for $C_{22}H_{22}F_3N_3O_3$ [M+H]$^+$: *m/z* = 470.1686 (calcd), 470.1682 (found). Mp: 261-263 °C

Structure was also confirmed by X-Ray crystallography.

Synthesis of 4 – (1-(1,3-dioxo-2-pentyl-2,3-dihydro-1H-benzo[de]isoquinolin-6-yl)-3-(4-(trifluoromethyl)phenyl)thiourea)

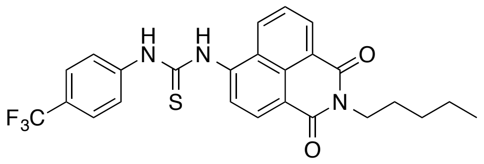

4, 43%

The intermediate isothiocyanate was synthesised from an adapted method of Anzenbacher Jr *et al.*[2] Compound **8** (0.5 g, 1.77 mmol, 1 equiv) was dissolved in acetone (70 mL). Thiophosgene (0.28 mL, 3.72 mmol, 2.1 equiv) was dissolved in acetone (25 mL) and added dropwise to the amine solution at 0 °C. The solution was allowed to warm to room temperature and stirred at room temperature for 2 days. The solvent was removed *in vacuo* and the resultant crude isothiocyanate (250 mg, 0.77 mmol, 1 equiv) was dissolved in $CHCl_3$ (10 mL). 4-(Trifluoromethyl)aniline (0.11 mL, 1 mmol, 1.2 equiv) was dissolved in $CHCl_3$ (5 mL) and added dropwise to the stirring isothiocyanate solution. The solution was stirred at reflux overnight and upon cooling the solvent removed. The crude solid was triturated in dichloromethane for 30 mins and the resultant solid filtered and dried under vacuum giving **4** as a yellow solid in 43% yield (160 mg, 0.33 mmol).

^{1}H NMR (400 MHz, DMSO-d_6) δ: 10.59 (s, 1H, urea NH), 10.53 (s, 1H, urea NH), 8.51 (m, 3H, aromatic CH x 3), 7.99 (d, 1H, aromatic CH, J = 7.95 Hz), 7.90 (t, 1H, J = 7.89 Hz), 7.83 (br d, 2H, aromatic CH x 2, J = 8.44 Hz), 7.71 (br d, 2H, aromatic CH x2, J = 8.44 Hz), 4.05 (br t, 2H, aliphatic CH_2 J = 7.34 Hz), 1.64 (br t, 2H, aliphatic CH_2), 1.33 (m, 4H, aliphatic CH_2 x 2), 0.87 (br t, 3H, aliphatic CH_3). ^{13}C{^{1}H} NMR (101 MHz, DMSO-d_6) δ: 181.1, 163.4, 162.9, 143.1, 141.6, 131.0, 129.6, 127.0, 125.7, 125.4, 123.3, 122.4, 119.5, 36.3, 28.4, 26.9, 21.6, 13.6. LRMS ESI$^+$ (m/z): 485.81 [M+H]$^+$. HRMS (ESI$^+$) for $C_{22}H_{22}F_3N_3O_2S$ [M+H]$^+$: *m/z* = 486.1458 (calcd), 486.1470 (found). Mp: 148-150 °C

Synthesis of 5 – (1-(3,5-bis(trifluoromethyl)phenyl)-3-(1,3-dioxo-2-pentyl-2,3-dihydro-1H-benzo[de]isoquinolin-6-yl)urea)

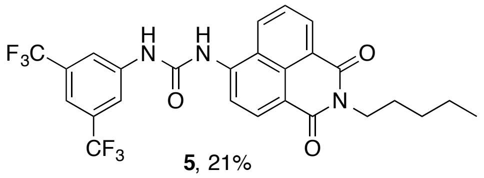

5, 21%

Compound **8** (400 mg, 1.46 mmol, 1eq) was dissolved in dichloromethane (100 mL). A saturated $NaHCO_3$ aqueous solution (100 mL) was added to this and the biphasic mixture stirred. Triphosgene (424 mg, 1.46 mmol, 3 eq) was dissolved in dichloromethane (15 mL) and added dropwise to the stirring solution and the reaction stirred at room temperature for 15 hours. The biphasic mixture was separated and the organic phase washed with H_2O (2 x 50 mL) and dried over $MgSO_4$. The organic solution filtered and concentrated to *ca.* 50 mL. 3,5 Bis-(trifluoromethyl) aniline (0.22 mL, 1.43 mmol, 1 eq) was added dropwise to the stirred solution and the reaction stirred for two days. The resultant precipitate was filtered and washed with cold dichloromethane (2 x 50 mL) and re-crystallised from dichloromethane affording compound **5**. The filtrate was reduced *in vacuo* and was re-crystallised a further time from boiling methanol. The resultant solid was filtered and dried under vacuum and was also found to be **5**. The combined solids gave **5** as a yellow solid in 21% yield (162 mg, 0.30 mmol).

^{1}H NMR (400 MHz, DMSO-d_6) δ: 9.84 (br s, 1H, urea NH), 9.50 (br s, 1H, urea NH), 8.48 (br t, 2H, aromatic CH x 2, J = 8.74 Hz), 8.40 (m, 2H, aromatic CH x 2), 8.12 (br t, 2H, aromatic CH x 2), 7.89 (br t, 1H, aromatic CH, J = 7.89 Hz), 7.68 (s, 1H, aromatic CH), 3.98 (br t, 2H, aliphatic CH_2, J = 7.27 Hz), 1.61 (br t, 2H aliphatic CH_2), 1.32 (m, 4H, aliphatic CH_2 x 2), 0.87 (br t, 6H, aliphatic CH_3 x 2, J = 6.30 Hz). ^{13}C{^{1}H} NMR (101 MHz, DMSO-d_6): 163.4, 162.8, 152.1, 141.1, 140.7, 132.0, 131, 130.8, 128.3, 127.7, 126.4, 122.8, 122.4, 121.9, 118.2, 116.2, 116.1, 115.1, 39.9, 28.7, 2701, 21.8, 13.8. LRMS ESI$^+$ (m/z): 538.20 [M+H]$^+$. HRMS (ESI$^+$) for $C_{26}H_{21}F_6N_3O_3$ [M+H]$^+$: *m/z* = 538.1560 (calcd), 538.1573 (found). Mp: > 260 °C (decomposition).

Synthesis of 6 – (1-(3,5-bis(trifluoromethyl)phenyl)-3-(1,3-dioxo-2-pentyl-2,3-dihydro-1H-benzo[de]isoquinolin-6-yl)thiourea)

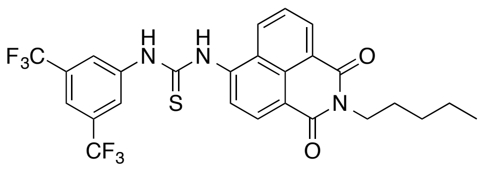

6, 60%

The intermediate isothiocyanate was synthesised from an adapted method of Anzenbacher Jr *et al.*[2] Compound **8** (0.5 g, 1.77 mmol, 1 equiv) was dissolved in acetone (70 mL). Thiophosgene (0.28 mL, 3.72 mmol, 2.1 equiv) was dissolved in acetone (25 mL) and added dropwise to the amine solution at 0 °C. The solution was allowed to warm to room temperature and stirred for 2 days. The solvent was removed under *in vacuo* and the resultant crude isothiocyanate was dissolved in $CHCl_3$ (10 mL). 3,5 Bis-(trifluoromethyl) aniline (0.28 mL, 1 mmol, 1 equiv) was dissolved in $CHCl_3$ (5 mL) and added dropwise to the stirring isothiocyanate solution. The solution was stirred at room temperature for 2 days and the solvent reduced *in vacuo*. Compound **6** was purified by column chromatography on silica gel using 1% MeOH/dichloromethane giving affording compound **6** as an orange solid in 60% yield (0.580 g, 1.05 mmol).

^{1}H NMR (400 MHz, DMSO-*d*$_6$) δ: 10.80 (s, 1H, thiourea NH), 10.52 (s, 1H, thiourea NH), 8.50 (m, 3H, aromatic CH x 3), 8.30 (s, 2H, aromatic CH x 2), 7.97 (d, 1H, aromatic CH, J = 7.95 Hz), 7.92 (t, 1H, aromatic CH, J = 7.89 Hz), 7.84 (s, 1H, aromatic CH), 4.05 (br t, 2H, aliphatic CH_2, J = 7.21 Hz), 1.64 (br t, 2H, aliphatic CH_2), 1.33 (m, 4H, aliphatic CH_2 x 2), 0.87 (br t, 3H, aliphatic CH_3, J = 6.42 Hz). ^{13}C{^{1}H} NMR (101 MHz, DMSO-*d*$_6$) δ: 181.5, 163.3, 162.9, 141.5, 141.1, 131.1, 130 129.9, 128.4, 127.4, 127.2, 125.1, 123.8, 122.4, 119.9, 117.5, 117.4,39.3, 28.6, 27.1, 21.8, 13.8. LRMS ESI$^+$ (m/z): 554.08 [M+H]$^+$. HRMS (ESI$^+$) for $C_{26}H_{21}F_6N_3O_2S$ [M+H]$^+$: *m/z* = 554.1331 (calcd), 554.1327 (found). Mp: 141-145 °C.

3 – Characterisation

3.1 NMR Spectra

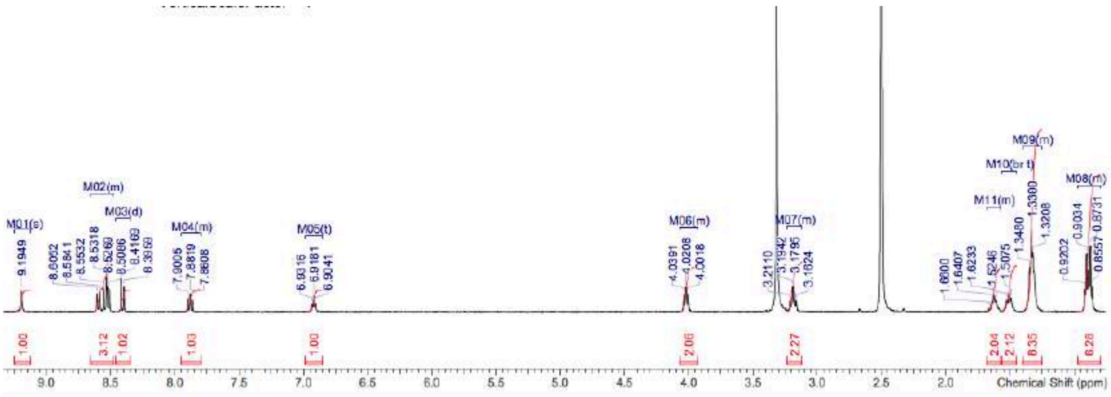

Figure S1: ^{1}H NMR spectrum of compound **1** in DMSO-d_6 at 298 K.

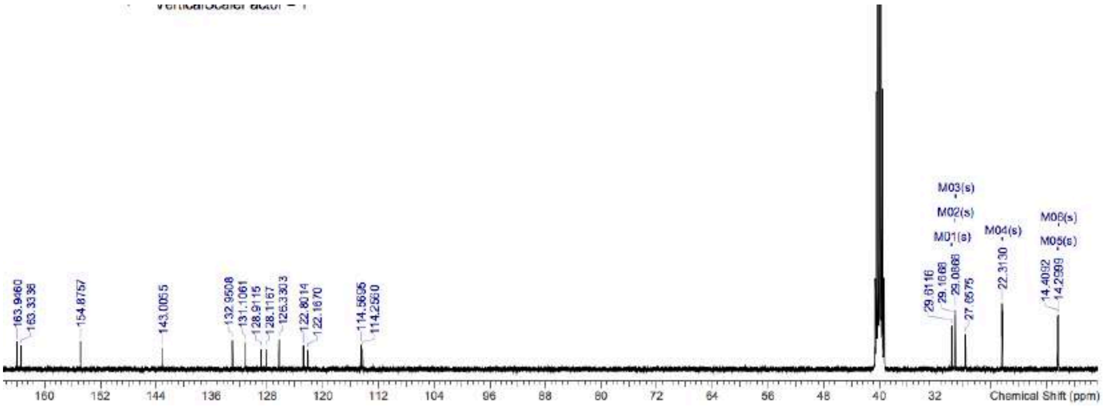

Figure S2: ^{13}C NMR spectrum of compound **1** in DMSO-d_6 at 298 K.

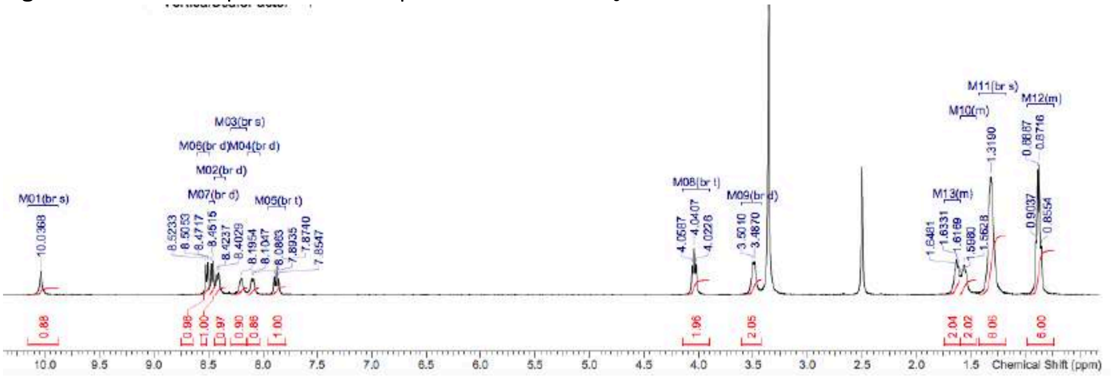

Figure S3: ^{1}H NMR spectrum of compound **2** in DMSO-d_6 at 298 K.

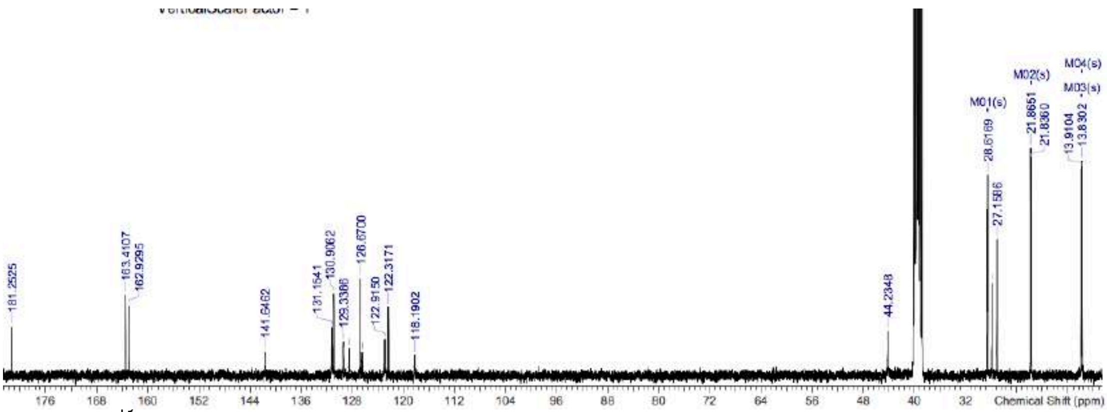

Figure S4: ^{13}C NMR spectrum of compound **2** in DMSO-d_6 at 298 K.

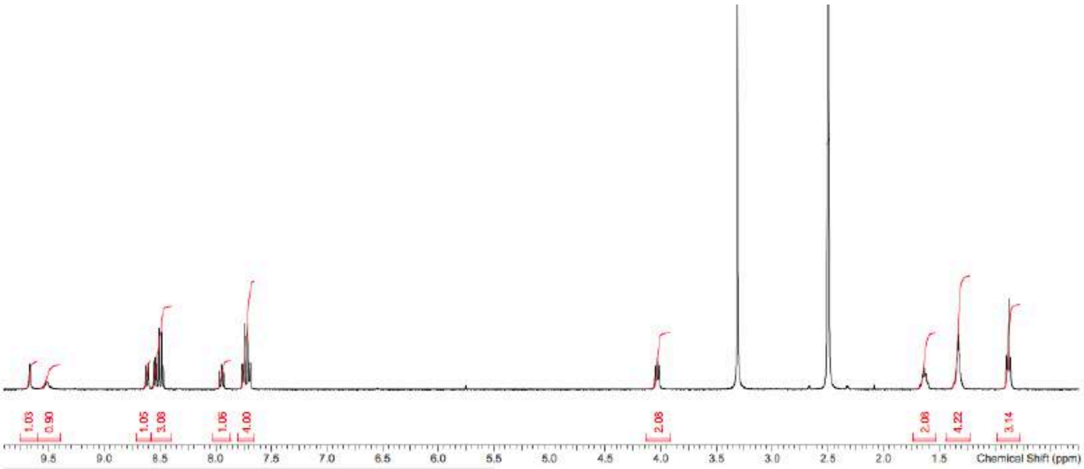

Figure S5: ^{1}H NMR spectrum of compound **3** in DMSO-d_6 at 298 K.

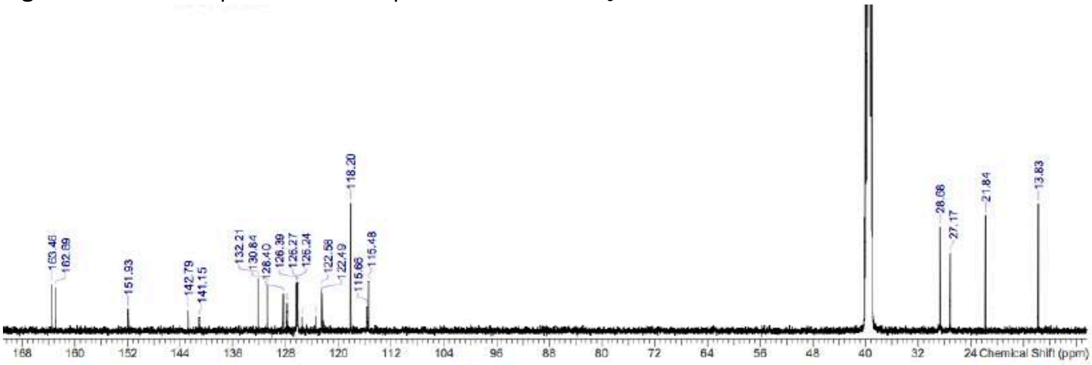

Figure S6: ^{13}C NMR spectrum of compound **3** in DMSO-d_6 at 298 K.

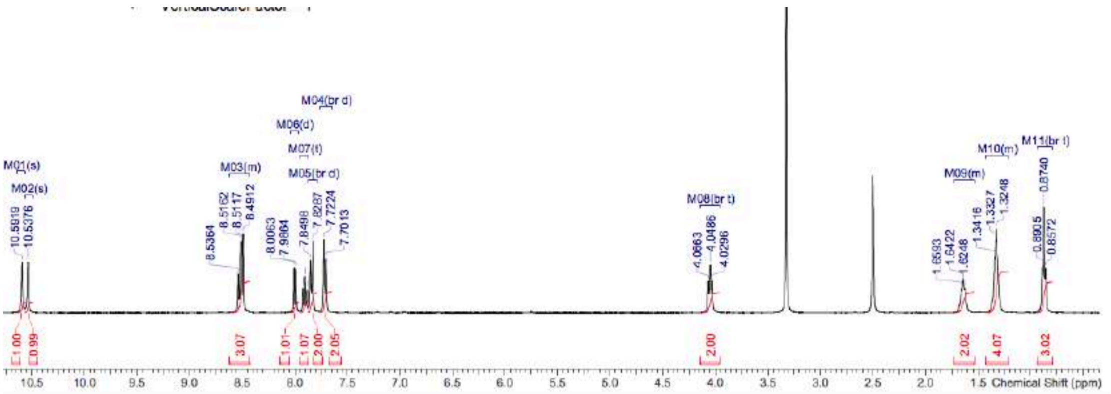

Figure S7: ^{1}H NMR spectrum of compound **4** in DMSO-d_6 at 298 K.

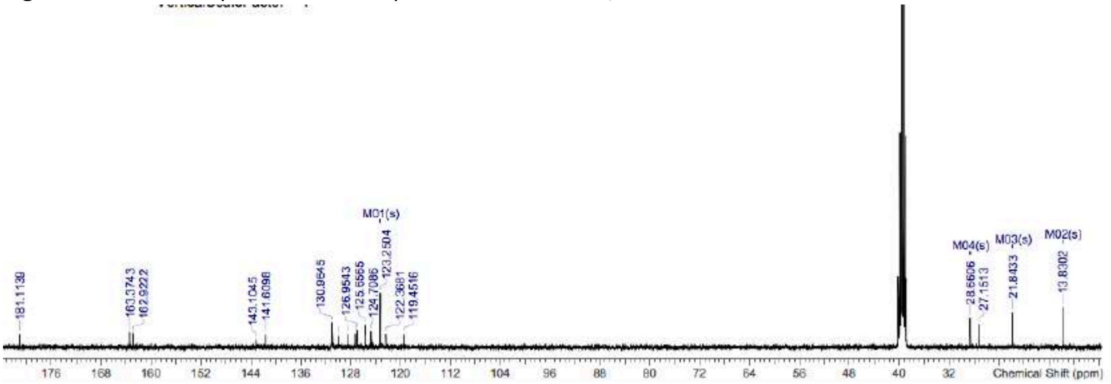

Figure S8: ^{13}C NMR spectrum of compound **4** in DMSO-d_6 at 298 K.

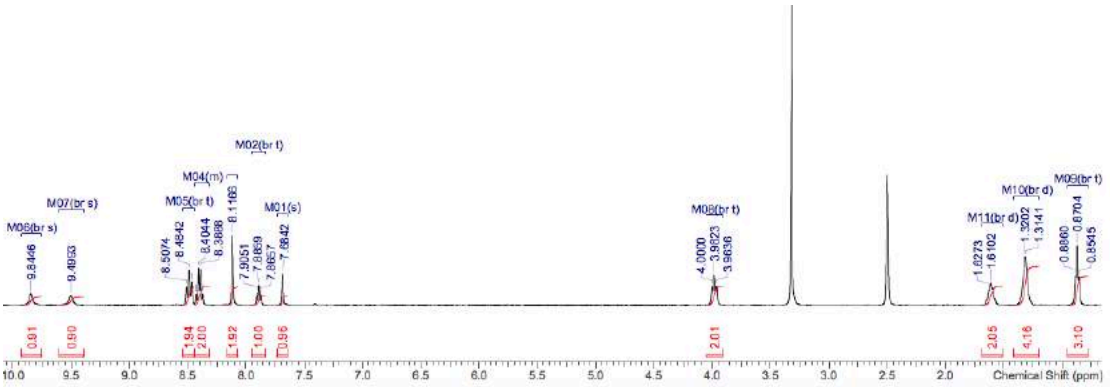

Figure S9: ^{1}H NMR spectrum of compound **5** in DMSO-d_6 at 298 K.

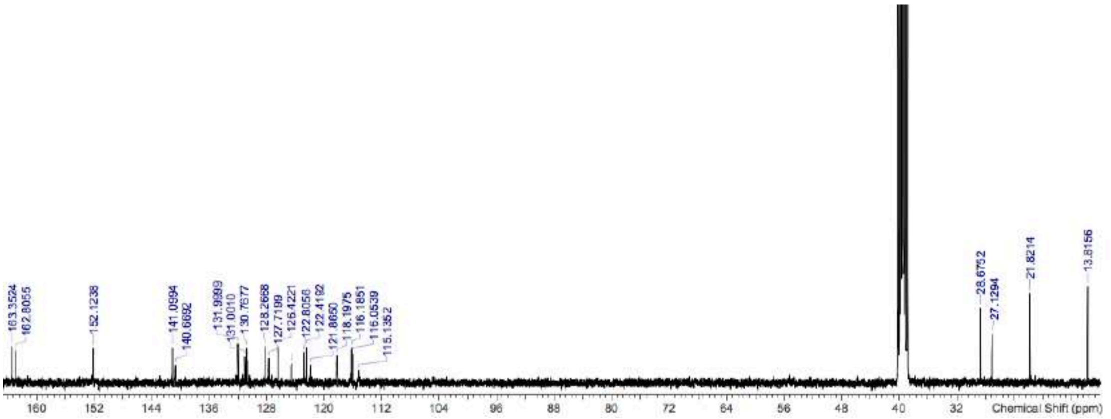

Figure S10: ^{13}C NMR spectrum of compound **5** in DMSO-d_6 at 298 K.

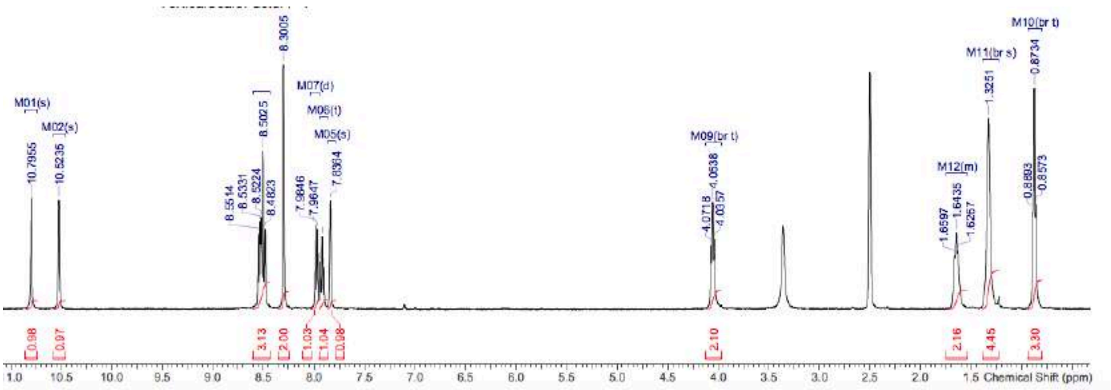

Figure S11: ^{1}H NMR spectrum of compound **6** in DMSO-d_6 at 298 K.

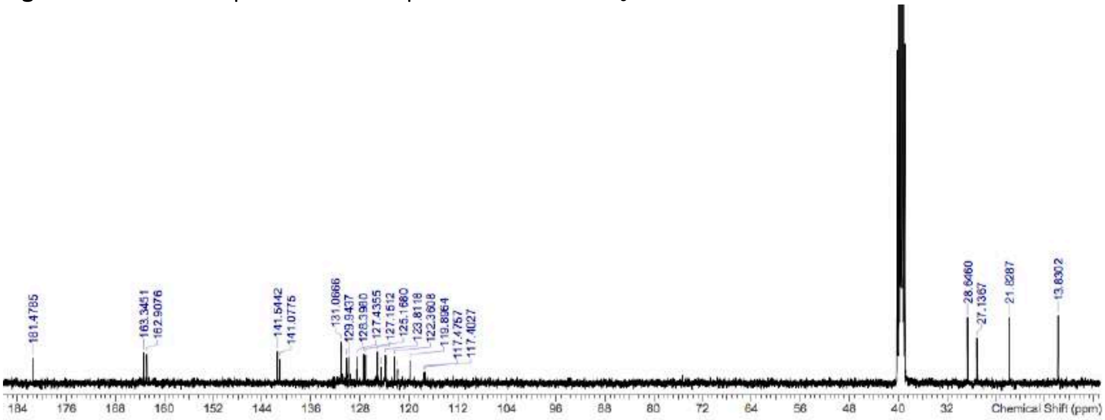

Figure S12: ^{13}C NMR spectrum of compound **6** in DMSO-d_6 at 298 K.

3.2 High Resolution Mass Spectrometry (HRMS)

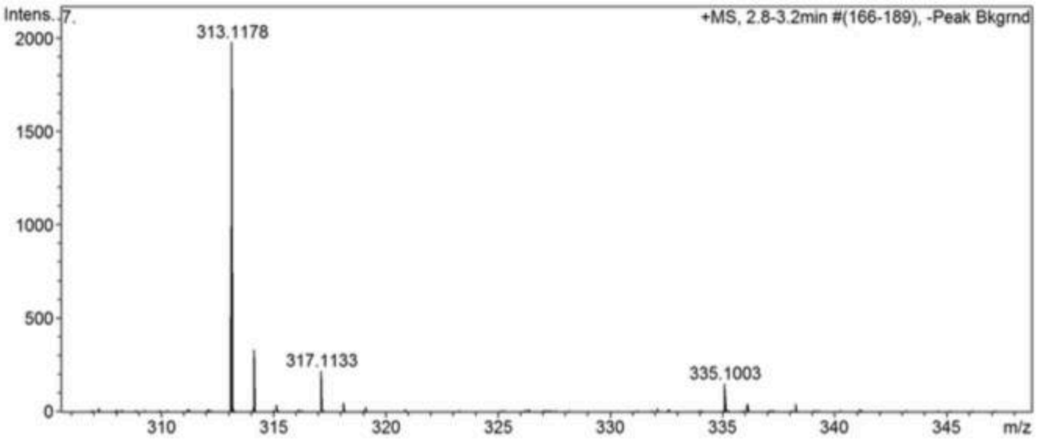

Meas. m/z	Formula	m/z	err [ppm]	err [mDa]	# Sigma	mSigma	rdb	e⁻ Conf	N-Rule
313.1178	C 15 H 15 N 5 O 3	313.1169	-2.6	-0.8	1	14.5	11.0	odd	ok
	C 16 H 14 N 6 Na	313.1172	-1.7	-0.5	2	18.9	12.5	even	ok
	C 17 H 17 N 2 O 4	313.1183	1.7	0.5	3	20.0	10.5	even	ok
	C 18 H 16 N 3 Na O	313.1186	2.6	0.8	4	24.8	12.0	odd	ok

Figure S13: HRMS of **7**.

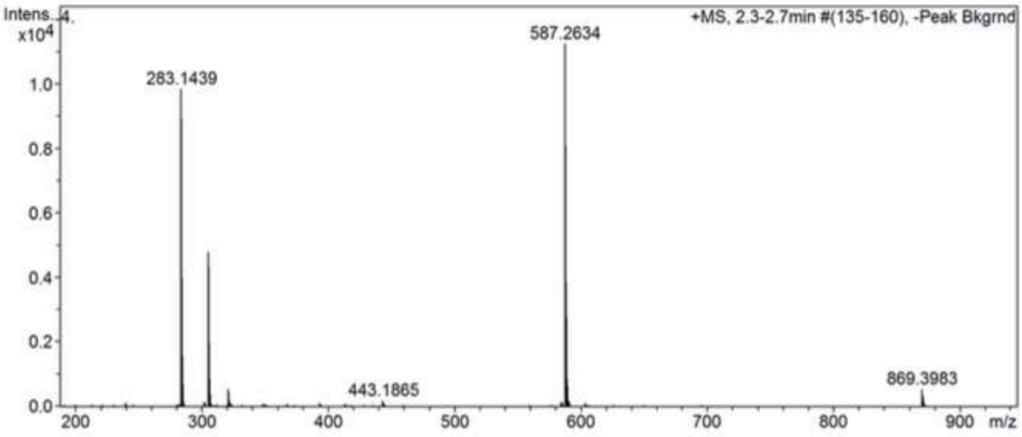

Meas. m/z	Formula	m/z	err [ppm]	err [mDa]	# Sigma	mSigma	rdb	e⁻ Conf	N-Rule
283.1439	C 17 H 19 N 2 O 2	283.1441	0.8	0.2	1	9.7	9.5	even	ok
305.1260	C 15 H 13 N 8	305.1258	-0.7	-0.2	1	10.2	13.5	even	ok
	C 17 H 18 N 2 Na O 2	305.1260	0.2	0.1	2	12.7	9.5	even	ok
	C 16 H 19 N O 5	305.1258	-0.7	-0.2	3	18.3	8.0	odd	ok
587.2634	C 33 H 37 N 3 O 7	587.2626	-1.4	-0.8	1	15.9	17.0	odd	ok
	C 35 H 39 O 8	587.2639	0.9	0.5	2	21.9	16.5	even	ok
	C 32 H 31 N 10 O 2	587.2626	-1.4	-0.8	3	22.7	22.5	even	ok
	C 34 H 36 N 4 Na O 4	587.2629	-0.9	-0.6	4	22.9	18.5	even	ok
	C 34 H 33 N 7 O 3	587.2639	0.9	0.5	5	28.3	22.0	odd	ok
	C 36 H 38 N Na O 5	587.2642	1.3	0.8	6	28.7	18.0	odd	ok
	C 35 H 32 N 8 Na	587.2642	1.3	0.8	7	35.4	23.5	even	ok

Figure S14: HRMS of **8**.

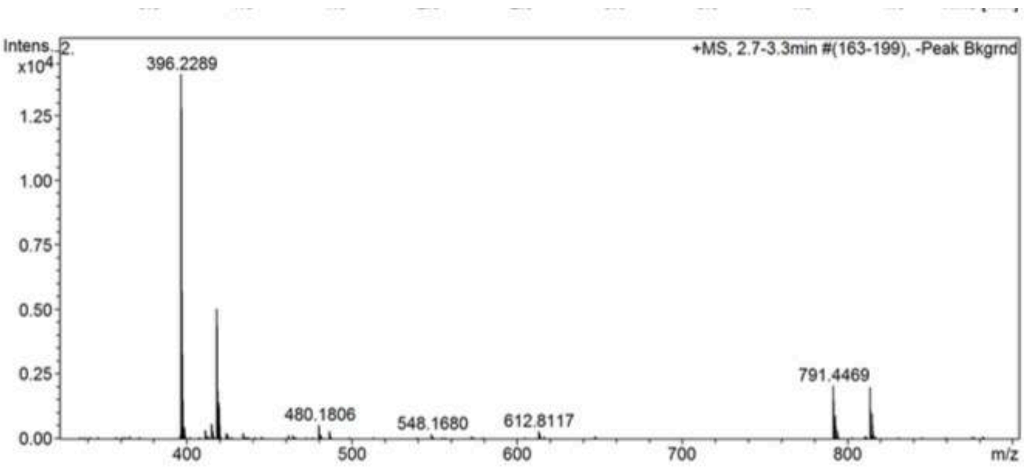

Meas. m/z	Formula	m/z	err [ppm]	err [mDa]	# Sigma	mSigma	rdb	e⁻ Conf	N-Rule
396.2289	C 23 H 30 N 3 O 3	396.2282	-1.9	-0.8	1	4.0	10.5	even	ok
	C 26 H 31 N Na O	396.2298	2.1	0.8	2	18.6	11.5	even	ok

Figure S15: HRMS of **1**.

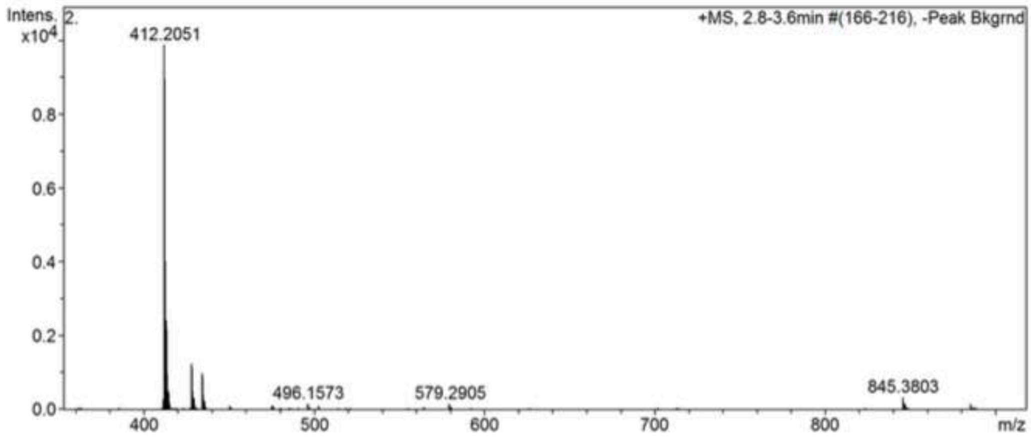

Meas. m/z	Formula	m/z	err [ppm]	err [mDa]	# Sigma	mSigma	rdb	e⁻ Conf	N-Rule
412.2051	C 23 H 30 N 3 O 2 S	412.2053	0.6	0.2	1	20.2	10.5	even	ok
	C 17 H 31 N 3 Na O 7	412.2054	0.8	0.3	2	28.2	3.5	even	ok

Figure S16: HRMS of **2**.

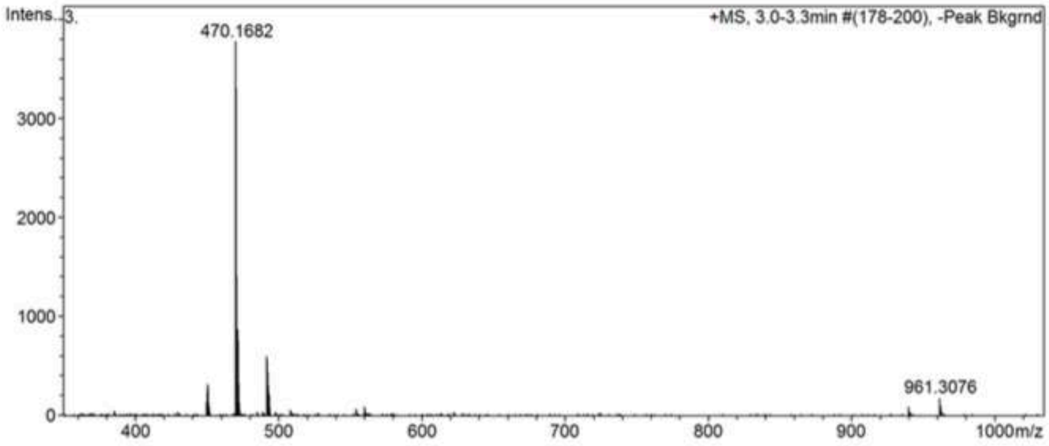

Meas. m/z	Formula	m/z	err [ppm]	err [mDa]	# Sigma	mSigma	rdb	e⁻ Conf	N-Rule
470.1682	C 19 H 25 F N 5 O 8	470.1682	0.0	0.0	1	3.5	9.5	even	ok
	C 19 H 18 F N 13 Na	470.1684	0.6	0.3	2	15.1	16.5	even	ok
	C 15 H 23 F 2 N 9 Na O 5	470.1682	0.2	0.1	3	21.3	7.5	even	ok
	C 15 H 30 F 2 N O 13	470.1680	-0.4	-0.2	4	29.6	0.5	even	ok
	C 25 H 23 F 3 N 3 O 3	470.1686	1.0	0.4	5	29.8	14.5	even	ok
	C 25 H 25 N 3 Na O 5	470.1686	1.0	0.5	6	30.0	14.5	even	ok
	C 23 H 20 N 9 O 3	470.1684	0.4	0.2	7	30.1	18.5	even	ok
	C 28 H 22 F 2 N 3 O 2	470.1675	-1.5	-0.7	8	42.4	18.5	even	ok
	C 31 H 23 F 2 N Na	470.1691	2.0	0.9	9	55.1	19.5	even	ok

Figure S17: HRMS of **3**.

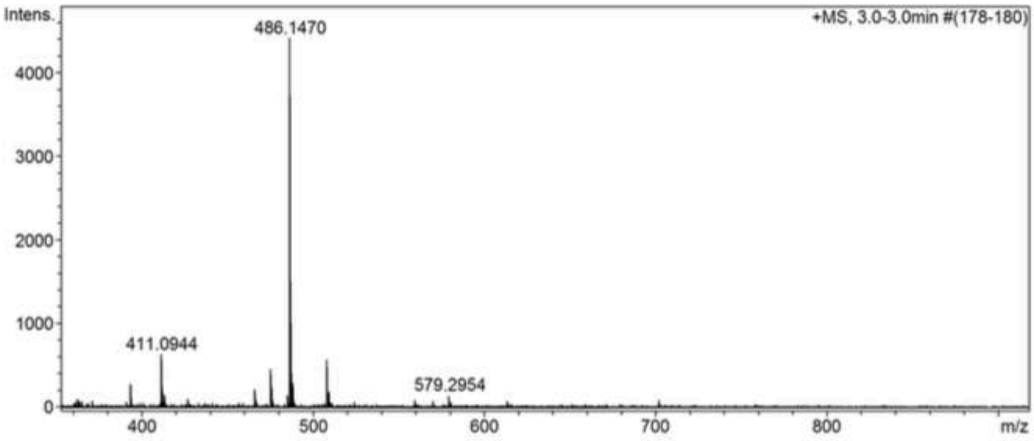

Meas. m/z	Formula	m/z	err [ppm]	err [mDa]	# Sigma	mSigma	rdb	e⁻ Conf	N-Rule
486.1470	C 24 H 22 F 2 N 3 O 6	486.1471	0.1	0.1	1	9.7	14.5	even	ok
	C 20 H 21 F N 9 O 3 S	486.1467	-0.8	-0.4	2	13.2	14.5	even	ok
	C 23 H 22 F N 7 Na O S	486.1483	2.5	1.2	3	13.2	15.5	even	ok
	C 23 H 19 F 2 N 7 Na O 2	486.1461	-2.1	-1.0	4	14.1	16.5	even	ok
	C 22 H 26 F N 3 Na O 5 S	486.1469	-0.2	-0.1	5	15.1	10.5	even	ok
	C 21 H 14 F 2 N 13	486.1458	-2.6	-1.3	6	16.8	20.5	even	ok
	C 27 H 21 F N 3 O 5	486.1460	-2.2	-1.1	7	17.5	18.5	even	ok
	C 25 H 23 F 3 N 3 O 2 S	486.1458	-2.7	-1.3	8	17.5	14.5	even	ok
	C 25 H 18 F 2 N 7 O 2	486.1485	2.9	1.4	9	18.2	19.5	even	ok
	C 21 H 25 N 3 Na O 9	486.1483	2.6	1.3	10	18.7	10.5	even	ok
	C 25 H 25 N 3 Na O 4 S	486.1458	-2.6	-1.2	11	19.3	14.5	even	ok
	C 19 H 20 N 9 O 7	486.1480	2.0	1.0	12	22.6	14.5	even	ok
	C 21 H 23 F 3 N 3 O 7	486.1483	2.5	1.2	13	22.7	10.5	even	ok
	C 20 H 20 F 3 N 7 Na O 3	486.1472	0.3	0.1	14	23.9	12.5	even	ok
	C 18 H 17 N 13 Na O 3	486.1470	-0.2	-0.1	15	23.9	16.5	even	ok
	C 19 H 27 F 2 N 3 Na O 6 S	486.1481	2.1	1.0	16	25.3	6.5	even	ok
	C 18 H 15 F 3 N 13 O	486.1469	-0.3	-0.1	17	26.6	16.5	even	ok
	C 26 H 21 N 7 Na S	486.1471	0.2	0.1	18	27.7	19.5	even	ok
	C 28 H 24 F 3 N Na S	486.1474	0.7	0.3	19	28.0	15.5	even	ok
	C 18 H 24 N 5 O 11	486.1467	-0.8	-0.4	20	28.3	9.5	even	ok
	C 27 H 24 N 3 O 4 S	486.1482	2.4	1.2	21	29.1	17.5	even	ok
	C 30 H 22 F N Na O 3	486.1476	1.1	0.5	22	29.3	19.5	even	ok
	C 28 H 17 F N 7 O	486.1473	0.5	0.3	23	29.9	23.5	even	ok
	C 19 H 24 F 3 N 3 Na O 7	486.1459	-2.5	-1.2	24	34.9	7.5	even	ok
	C 33 H 19 F 3 N	486.1464	-1.3	-0.6	25	45.3	23.5	even	ok
	C 33 H 21 N Na O 2	486.1465	-1.2	-0.6	26	45.4	23.5	even	ok
	C 31 H 16 N 7	486.1462	-1.8	-0.9	27	45.6	27.5	even	ok

Figure S18: HRMS of **4**.

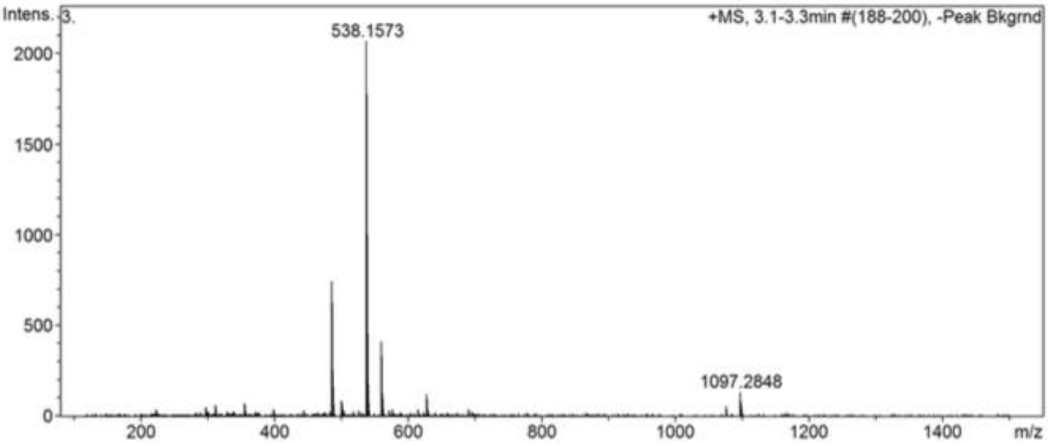

Meas. m/z	Formula	m/z	err [ppm]	err [mDa]	# Sigma	mSigma	rdb	e⁻ Conf	N-Rule
538.1573	C 20 H 17 F 4 N 13 Na	538.1558	-2.7	-1.4	1	19.8	16.5	even	ok
	C 22 H 16 F 4 N 13	538.1582	1.8	1.0	2	23.1	19.5	even	ok
	C 21 H 20 F 4 N 9 O 4	538.1569	-0.7	-0.4	3	24.8	14.5	even	ok
	C 24 H 21 F 4 N 7 Na O 2	538.1585	2.3	1.2	4	25.6	15.5	even	ok
	C 23 H 25 F 4 N 3 Na O 6	538.1572	-0.2	-0.1	5	27.3	10.5	even	ok
	C 24 H 19 F 3 N 9 O 3	538.1557	-2.8	-1.5	6	29.0	18.5	even	ok
	C 26 H 22 F 6 N 3 O 3	538.1560	-2.4	-1.3	7	29.1	14.5	even	ok
	C 26 H 24 F 3 N 3 Na O 5	538.1560	-2.3	-1.2	8	31.3	14.5	even	ok
	C 29 H 23 F 6 N Na O	538.1576	0.6	0.3	9	32.3	15.5	even	ok
	C 27 H 20 F 3 N 7 Na O	538.1574	0.2	0.1	10	32.5	19.5	even	ok
	C 18 H 21 F 5 N 9 O 5	538.1580	1.4	0.8	11	33.8	10.5	even	ok
	C 28 H 23 F 3 N 3 O 5	538.1584	2.2	1.2	12	35.1	17.5	even	ok
	C 20 H 26 F 5 N 3 Na O 7	538.1583	1.9	1.0	13	35.6	6.5	even	ok
	C 32 H 22 F 5 N Na	538.1565	-1.5	-0.8	14	47.8	19.5	even	ok
	C 34 H 21 F 5 N	538.1589	3.0	1.6	15	58.8	22.5	even	ok

Figure S19: HRMS of **5**.

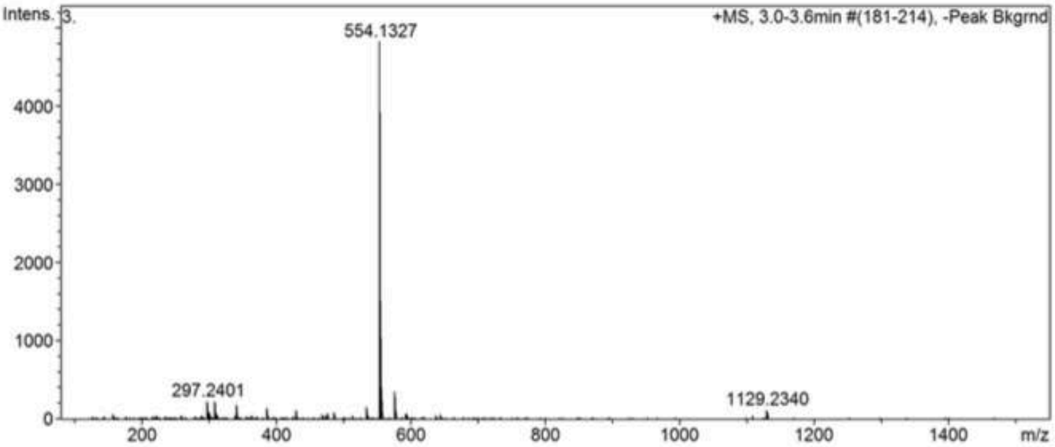

Meas. m/z	Formula	m/z	err [ppm]	err [mDa]	# Sigma	mSigma	rdb	e⁻ Conf	N-Rule
554.1327	C 23 H 22 F 5 N 3 Na O 6	554.1321	-1.0	-0.6	1	9.3	11.5	even	ok
	C 21 H 17 F 5 N 9 O 4	554.1318	-1.5	-0.9	2	11.6	15.5	even	ok
	C 24 H 18 F 5 N 7 Na O 2	554.1334	1.4	0.8	3	15.0	16.5	even	ok
	C 19 H 21 F 4 N 9 Na O 3 S	554.1316	-1.9	-1.0	4	15.3	11.5	even	ok
	C 22 H 13 F 5 N 13	554.1332	0.9	0.5	5	17.1	20.5	even	ok
	C 20 H 24 F 4 N 5 O 7 S	554.1327	0.1	0.0	6	19.4	9.5	even	ok
	C 23 H 23 F 3 N 5 O 6 S	554.1316	-2.0	-1.1	7	19.5	13.5	even	ok
	C 28 H 20 F 4 N 3 O 5	554.1334	1.2	0.7	8	24.5	18.5	even	ok
	C 18 H 20 F 3 N 9 Na O 7	554.1330	0.6	0.3	9	24.9	11.5	even	ok
	C 20 H 23 F 6 N 3 Na O 7	554.1332	1.0	0.6	10	25.1	7.5	even	ok
	C 26 H 22 F 6 N 3 O 2 S	554.1331	0.9	0.5	11	25.1	14.5	even	ok
	C 24 H 19 F 3 N 9 O 2 S	554.1329	0.4	0.2	12	25.4	18.5	even	ok
	C 18 H 18 F 6 N 9 O 5	554.1330	0.5	0.3	13	26.1	11.5	even	ok
	C 26 H 24 F 3 N 3 Na O 4 S	554.1332	0.9	0.5	14	26.7	14.5	even	ok
	C 27 H 17 F 4 N 7 Na O	554.1323	-0.7	-0.4	15	26.9	20.5	even	ok
	C 18 H 27 F 3 N O 15	554.1327	0.1	0.1	16	31.2	4.5	even	ok
	C 29 H 21 F 5 N 3 O S	554.1320	-1.2	-0.7	17	40.1	18.5	even	ok
	C 31 H 19 F 3 N 3 O 4	554.1322	-0.8	-0.5	18	40.8	22.5	even	ok
	C 32 H 15 F 3 N 7	554.1336	1.6	0.9	19	53.5	27.5	even	ok

Figure S20: HRMS of **6**.

3.3 UV-Vis Spectra

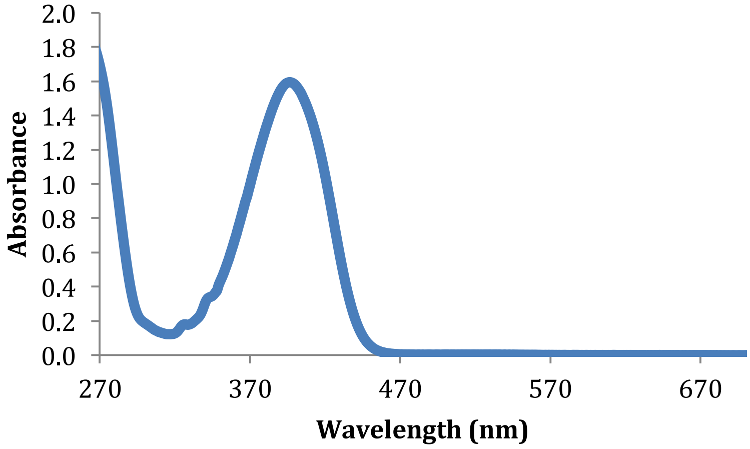

Figure S21: UV-Vis spectrum of compound **1** (0.1 mM in DMSO). ε = 15800 $M^{-1}cm^{-3}$.

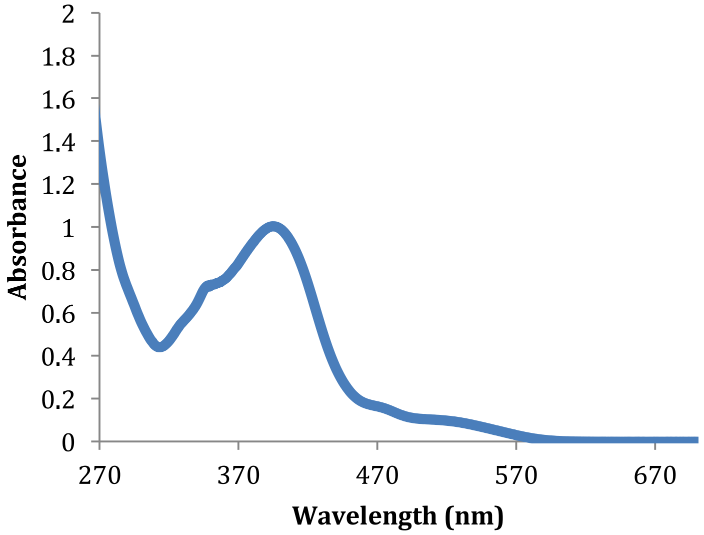

Figure S22: UV-Vis spectrum of compound **2** (0.1 mM in DMSO). ε = 9900 $M^{-1}cm^{-3}$.

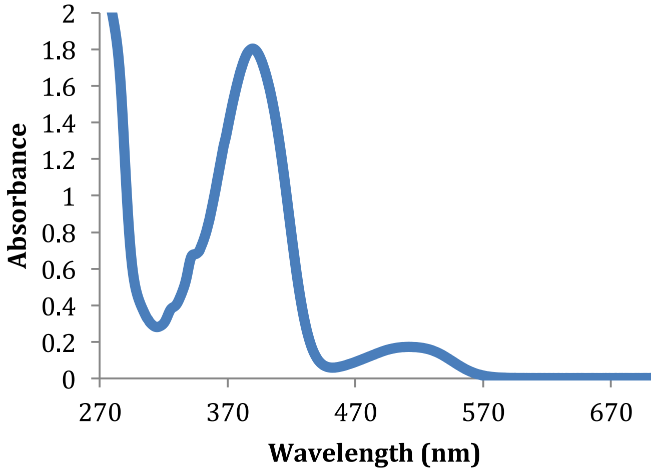

Figure S23: UV-Vis spectrum of compound **3** (0.1 mM in DMSO). ε = 17500 $M^{-1}cm^{-3}$.

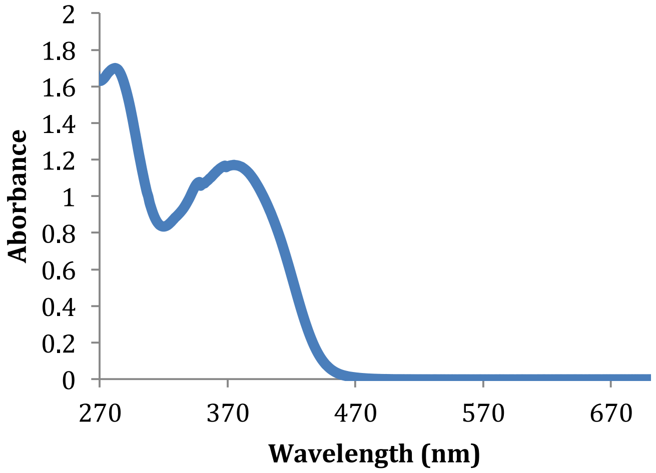

Figure S24: UV-Vis spectrum of compound **4** (0.1 mM in DMSO). ε = 14400 $M^{-1}cm^{-3}$.

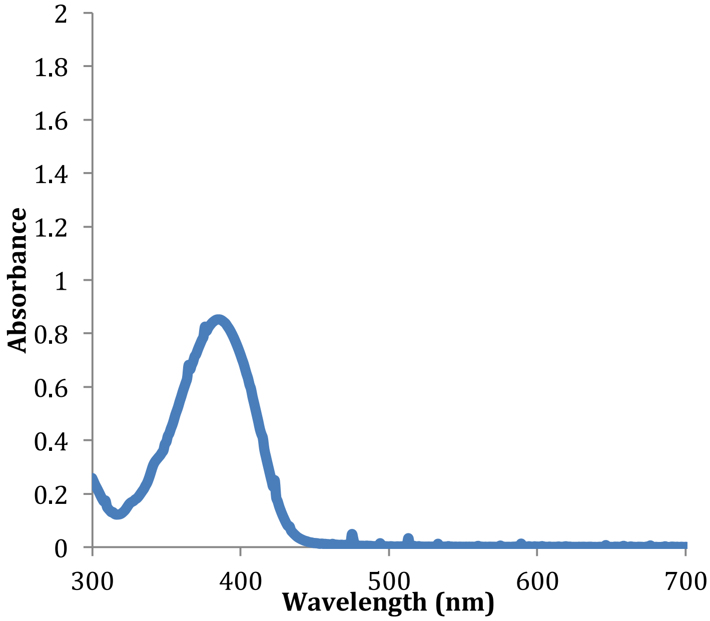

Figure S25: UV-Vis spectrum of compound **5** (0.05 mM in DMSO). ε = 16220 $M^{-1}cm^{-3}$.

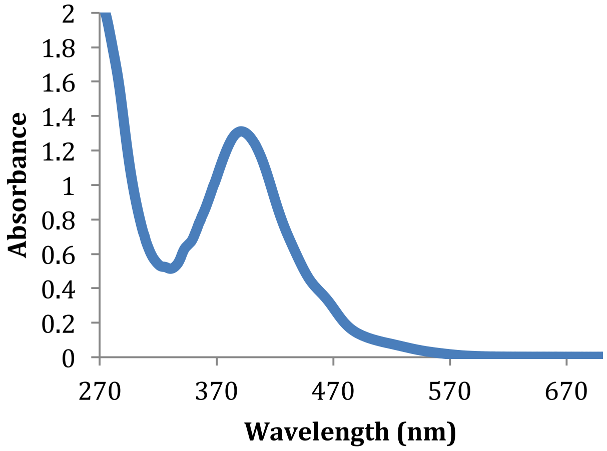

Figure S26: UV-Vis spectrum of compound **6** (0.1 mM in DMSO). ε = 12660 $M^{-1}cm^{-3}$.

3.4 Fluorescence Spectra

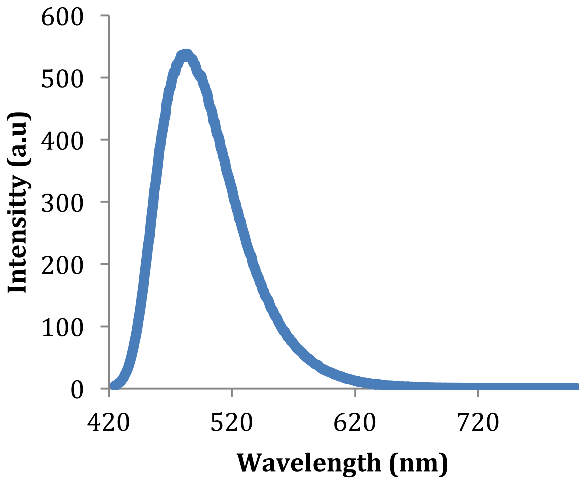

Figure S27: Fluorescence emission spectrum of compound **1** (1 μM in DMSO).

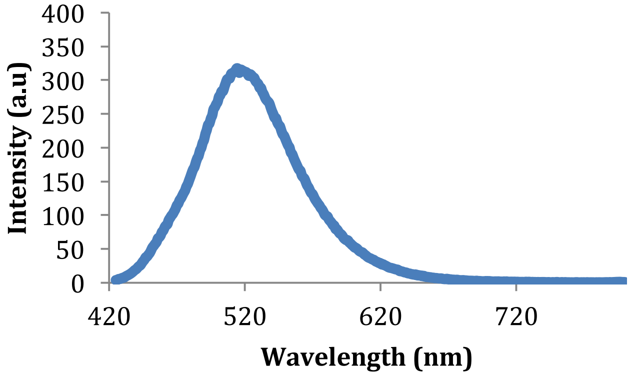

Figure S28: Fluorescence emission spectrum of compound **2** (1 μM in DMSO).

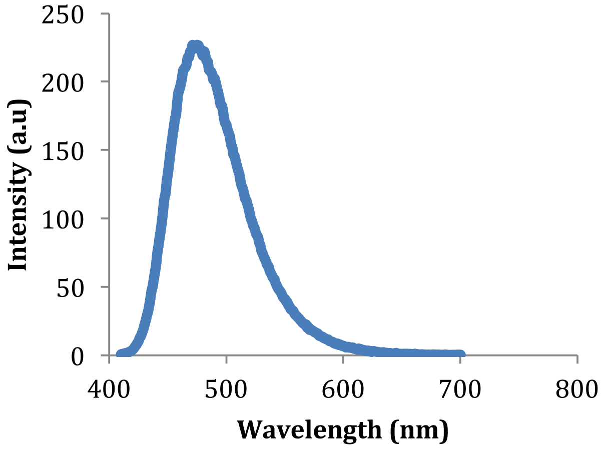

Figure S29: Fluorescence emission spectrum of compound **3** (1 μM in DMSO).

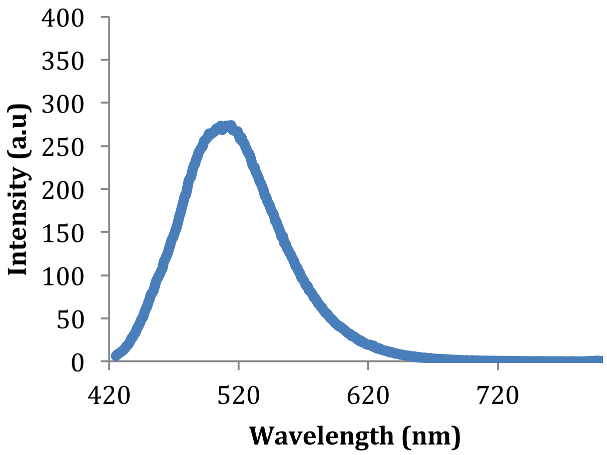

Figure S30: Fluorescence emission spectrum of compound **4** (1 μM in DMSO).

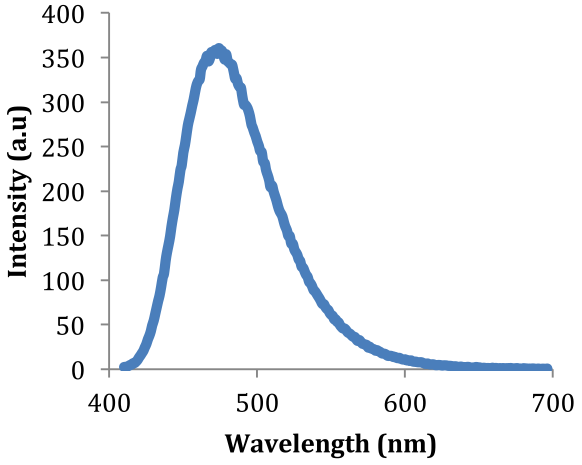

Figure S31: Fluorescence emission spectrum of compound **5** (1 μM in DMSO).

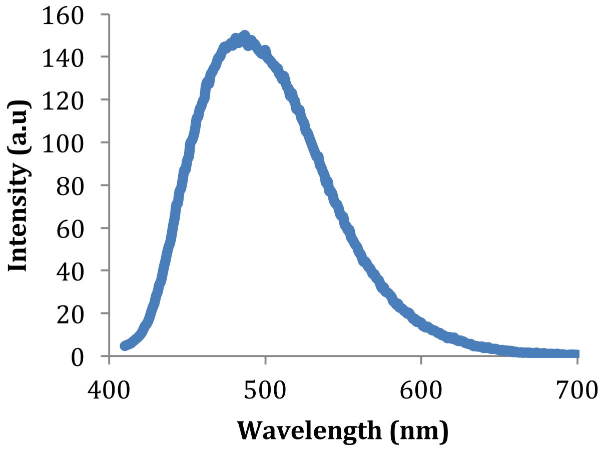

Figure S32: Fluorescence emission spectrum of compound **6** (1 μM in DMSO).

3.5 Single Crystal X-Ray Diffraction

Data was collected at the University of Southampton on a Rigaku AFC12 goniometer equipped with an enhanced sensitivity (HG) Saturn724+ detector mounted at the window of an FR-E+ SuperBright molybdenum rotating anode generator with HF Varimax optics (100 µm focus). Data collection: CrystalClear-SM Expert 3.1 b27 (Rigaku, 2013). Data reduction and cell refinement: CrysAlisPro, Agilent Technologies, Version 1.171.37.31. The structures were solved using ShelXT using direct methods and refined on *F2* by the full-matrix least-squares technique using the ShelXL program. Graphics were generated using Mercury 3.0 and Pov-Ray. The CIF has been deposited with the Cambridge Crystallographic Database Centre (CCDC 1424345).

4 – NMR Binding Studies

4.1 Overview and Procedure

Proton NMR titrations were performed as follows: A 1.5 mL 0.01M DMSO-d_6/0.5% H_2O solution of the receptor was prepared. Of this solution, 0.5 mL was added to an airtight sealed NMR tube. The remaining 1 mL of receptor solution was used to make a 0.15 M solution of a guest anion, which was added as the tetrabutylammonium salt (tetraethylammonium salt in the case of HCO_3^-). The receptor/salt solution was titrated into the NMR tube in small aliquots and a ^1H NMR spectrum taken after each addition. This ensures the overall concentration of receptor stays constant whilst the concentration of titrated anion changes. Chemical shifts are reported in ppm and were referenced to residual solvent peaks. A global analytical fitting using a 1:1 binding model was used to fit the data and obtain association constants (K_a).[3,4] A summary of NMR titrations performed is provided in Table S1.

Table S1: Overview of ^{1}H NMR (400 MHz) titration studies for receptors **1**-**6.** Association constants (M^{-1}) calculated by fitting the change in chemical shifts of both urea/thiourea NH signals and the naphthalimide CH signal to a 1:1 global fitting model. Chloride, nitrate and dihydrogen phosphate were added as tetrabutylammonium (TBA) salts and bicarbonate was added as tetraethylammonium (TEA) salt. Titrations were carried out in DMSO-d_6/0.5% water at 298 K.

	K_a Cl^- (M^{-1})	K_a NO_3^- (M^{-1})	K_a HCO_3^- (M^{-1})	K_a $H_2PO_4^-$ (M^{-1})
**1**	31	-[c]	-[d]	429
**2**	41[a]	-[c]	-[d]	-[d]
**3**	187	-[c]	-[d]	-[d]
**4**	49	-[c]	-[d]	-[d]
**5**	175	-[c]	-[d]	-[d]
**6**	53[b]	-[c]	-[d]	-[d]

[a] Stability constant calculated by fitting only naphthalimide NH proton signal due to overlapping peaks. [b] Association constant calculated by fitting both urea NH proton signals only. [c] No significant change in chemical shift observed. [d] Broadening and/or possible deprotonation of urea/thiourea NH proton signals observed and hence no fitting was carried out.

4.2 Interaction with TBACl

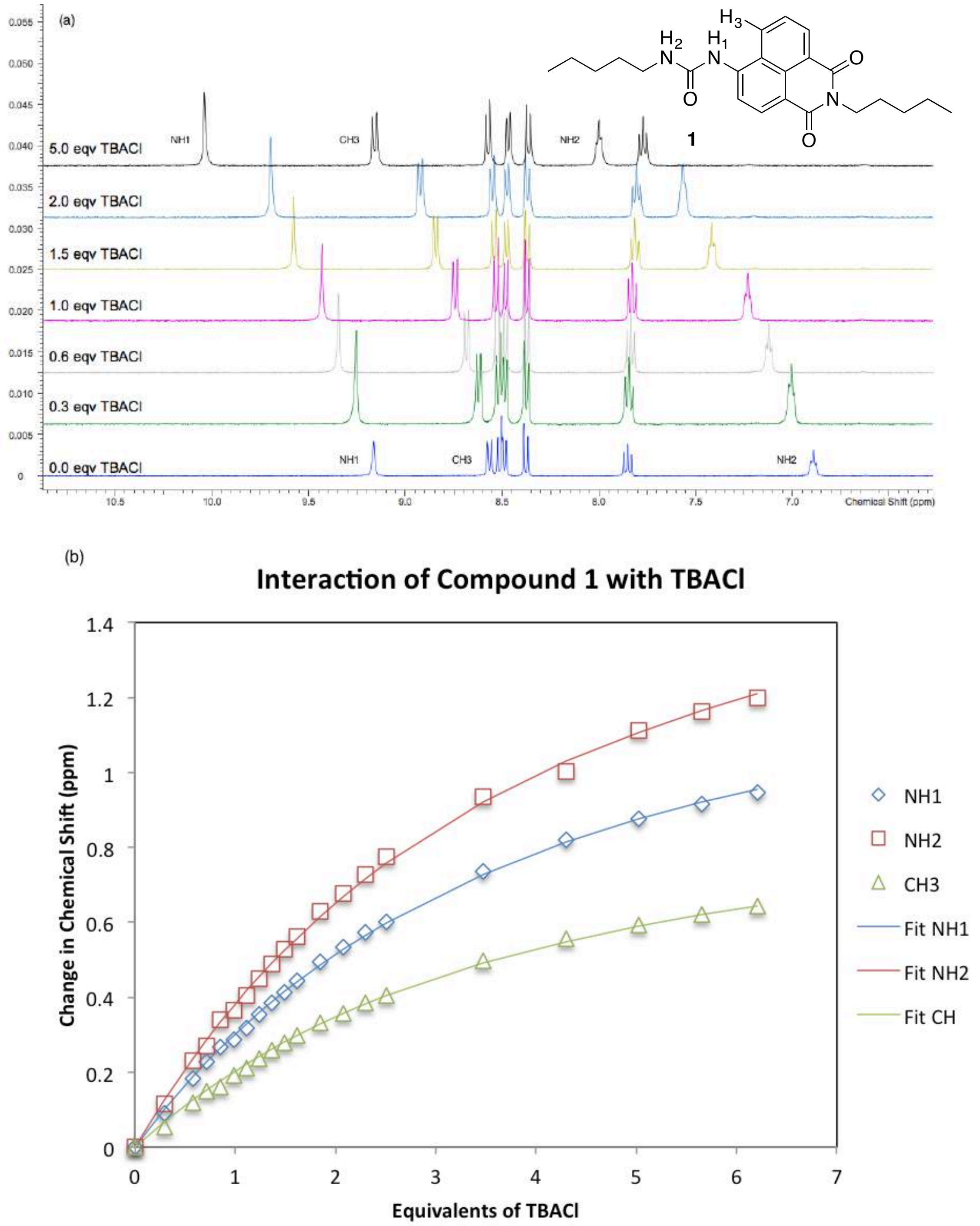

Figure S33: ^{1}H NMR titration of compound **1** with TBACl in DMSO-d_6 with 0.5% H_2O (400 MHz, 298 K). (a) Stack plot showing the aromatic region of **1**. (b) Change in chemical shifts of NH1, NH2 and CH3 during the course of the titration. Also shown are the fitted binding isotherms. K_a = 31.4 M^{-1}, covariance of the fit = 0.00867.

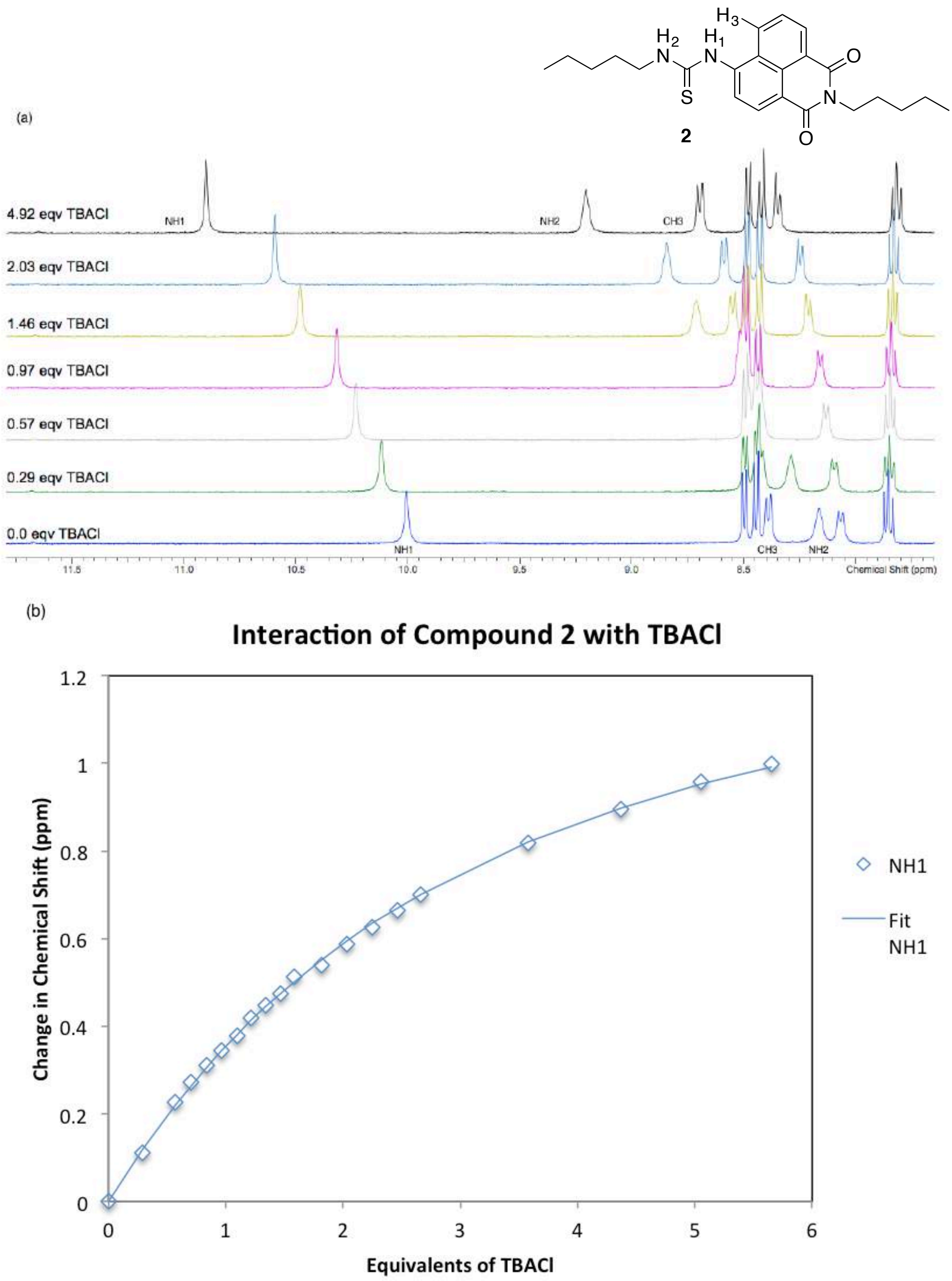

Figure S34: ^{1}H NMR titration of compound **2** with TBACl in DMSO-d_6 with 0.5% H_2O (400 MHz, 298 K). (a) Stack plot showing the aromatic region of **2**. (b) Change in chemical shifts of NH1 during the course of the titration. NH2 and CH3 could not be accurately fitted to a binding isotherm due to peak overlapping. Also shown are the fitted binding isotherms. K_a = 41 M^{-1}, covariance of the fit = 0.006135.

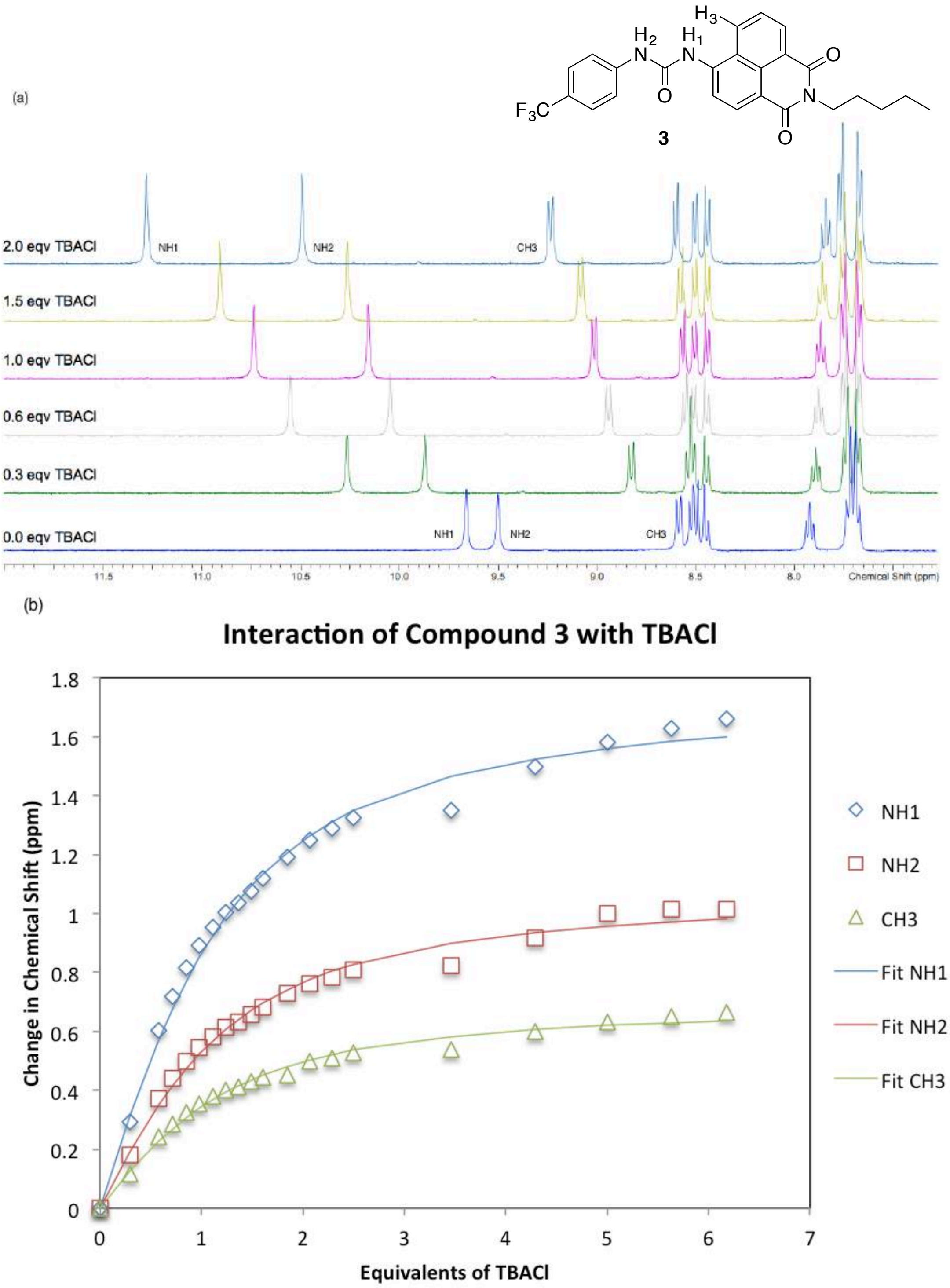

Figure S35: ^1H NMR titration of compound **3** with TBACl in DMSO-d_6 with 0.5% H_2O (400 MHz, 298 K). (a) Stack plot showing the aromatic region of **3**. (b) Change in chemical shifts of NH1, NH2 and CH3 during the course of the titration. Also shown are the fitted binding isotherms. K_a = 187 M^{-1}, covariance of the fit = 0.005025.

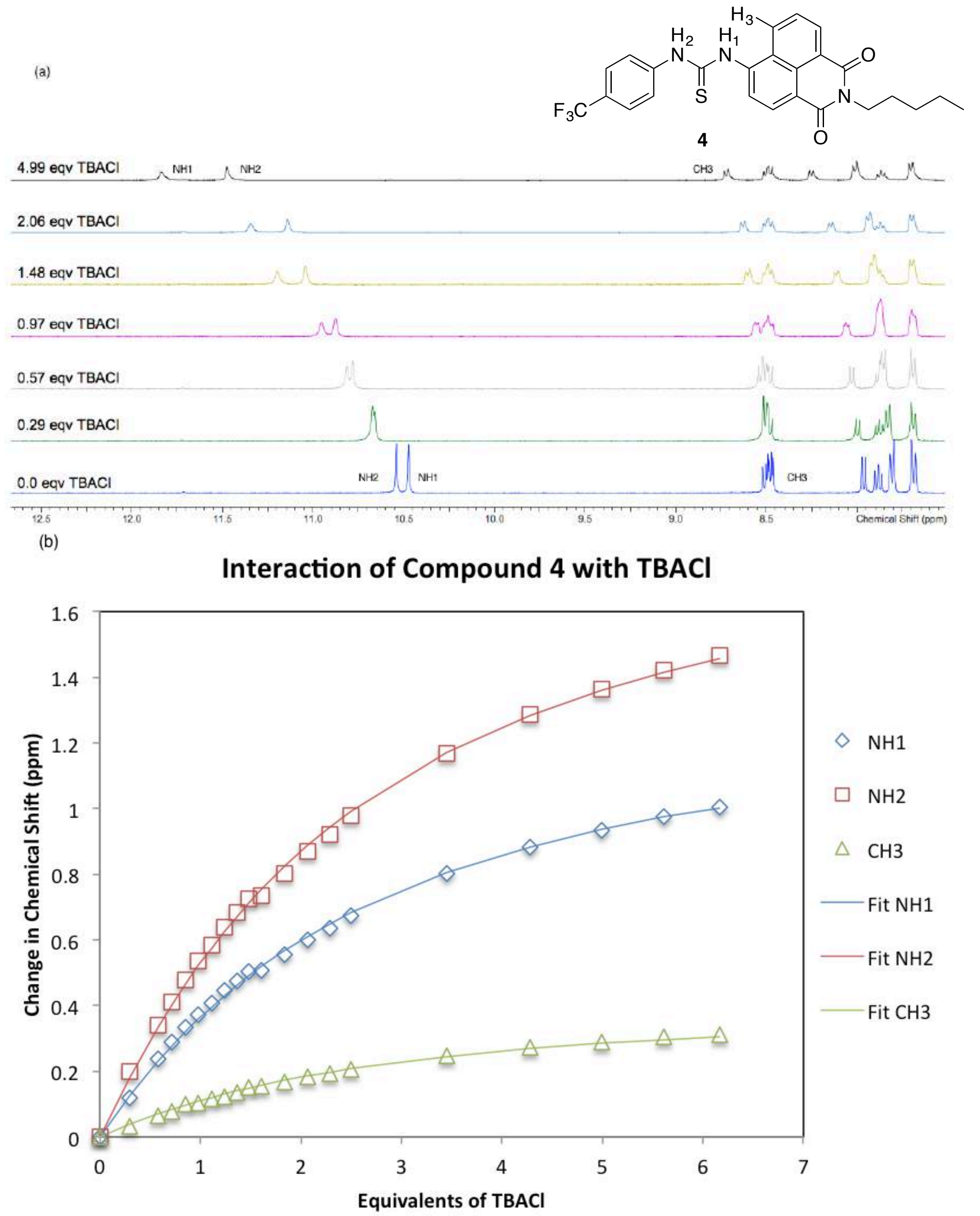

Figure S36: ^{1}H NMR titration of compound **4** with TBACl in DMSO-d_6 with 0.5% H_2O (400 MHz, 298 K). (a) Stack plot showing the aromatic region of **4**. (b) Change in chemical shifts of NH1, NH2 and CH3 during the course of the titration. Also shown are the fitted binding isotherms. K_a = 49.1 M^{-1}, covariance of the fit = 0.000652.

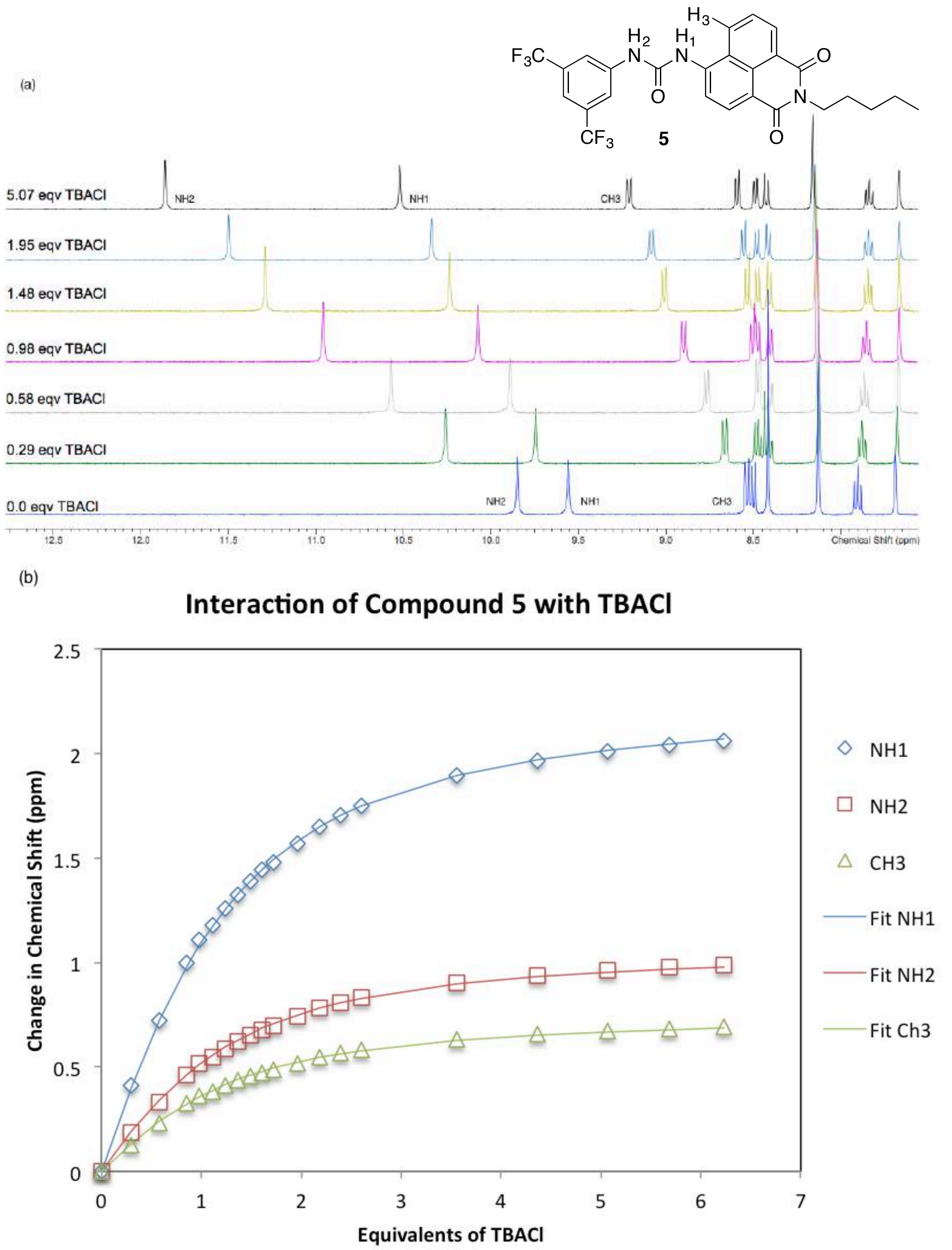

Figure S37: ^{1}H NMR titration of compound **5** with TBACl in DMSO-d_6 with 0.5% H_2O (400 MHz, 298 K). (a) Stack plot showing the aromatic region of **5**. (b) Change in chemical shifts of NH1, NH2 and CH3 during the course of the titration. Also shown are the fitted binding isotherms. K_a = 176 M^{-1}, covariance of the fit = 0.000189.

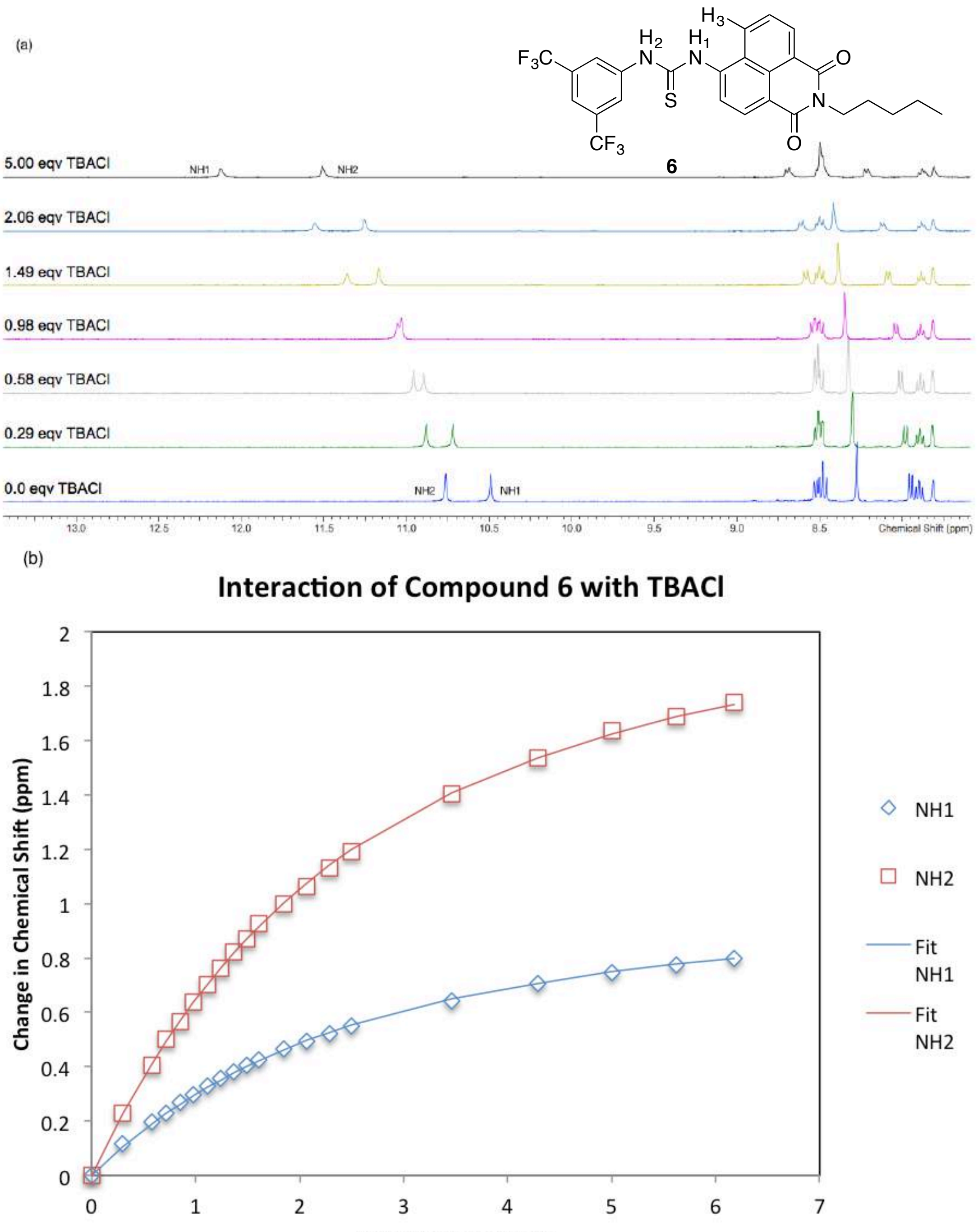

Figure S38: ^{1}H NMR titration of compound **6** with TBACl in DMSO-d_6 with 0.5% H_2O (400 MHz, 298 K). (a) Stack plot showing the aromatic region of **6**. (b) Change in chemical shifts of NH1 and NH2 during the course of the titration. Also shown are the fitted binding isotherms. K_a = 53.1 M^{-1}, covariance of the fit = 0.000155.

4.3 Interaction with $TEAHCO_3$

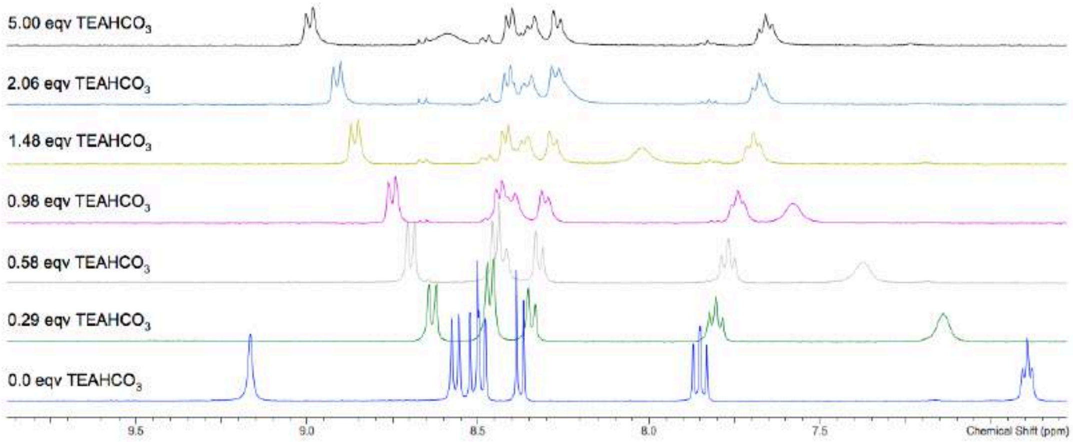

Figure S39: Stack plot showing the aromatic region of the ^{1}H NMR spectrum of **1** during the ^{1}H NMR titration with $TEAHCO_3$ in DMSO-d_6 with 0.5% H_2O (400 MHz, 298 K).

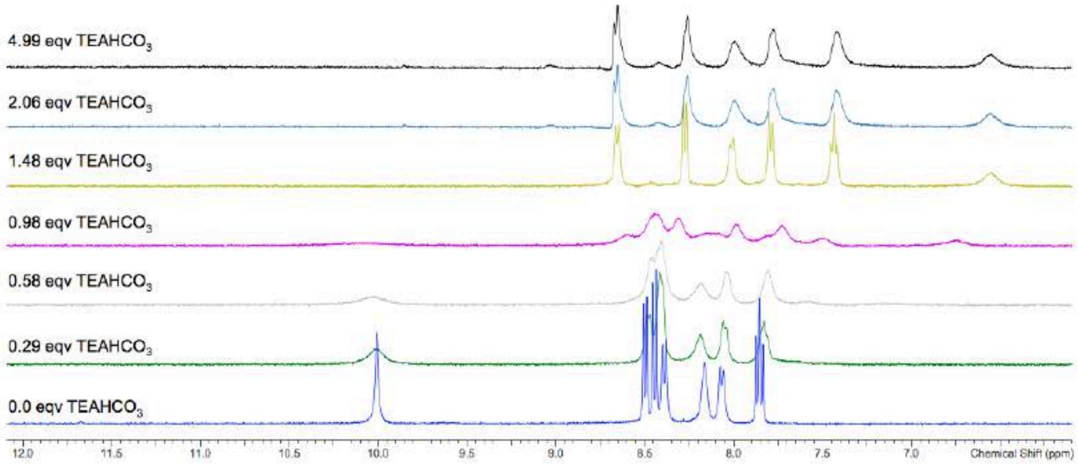

Figure S40: Stack plot showing the aromatic region of the ^{1}H NMR spectrum of **2** during the ^{1}H NMR titration with $TEAHCO_3$ in DMSO-d_6 with 0.5% H_2O (400 MHz, 298 K).

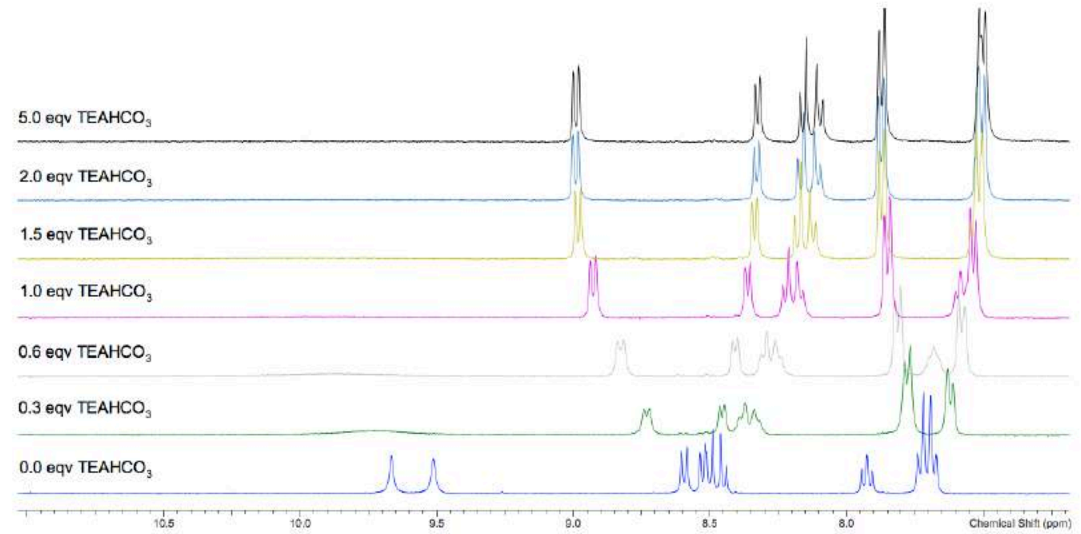

Figure S41: Stack plot showing the aromatic region of the ^{1}H NMR spectrum of **3** during the ^{1}H NMR titration with $TEAHCO_3$ in DMSO-d_6 with 0.5% H_2O (400 MHz, 298 K).

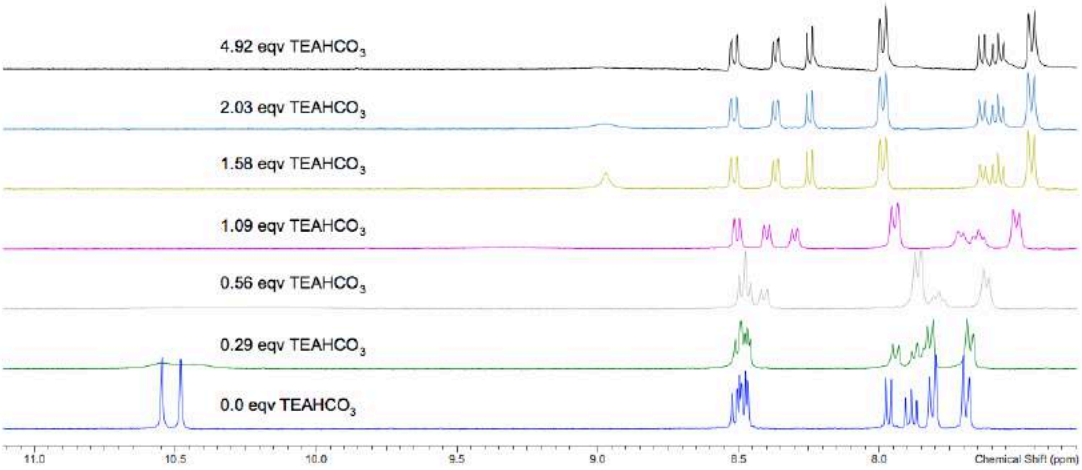

Figure S42: Stack plot showing the aromatic region of the ^{1}H NMR spectrum of **4** during the ^{1}H NMR titration with $TEAHCO_3$ in DMSO-d_6 with 0.5% H_2O (400 MHz, 298 K).

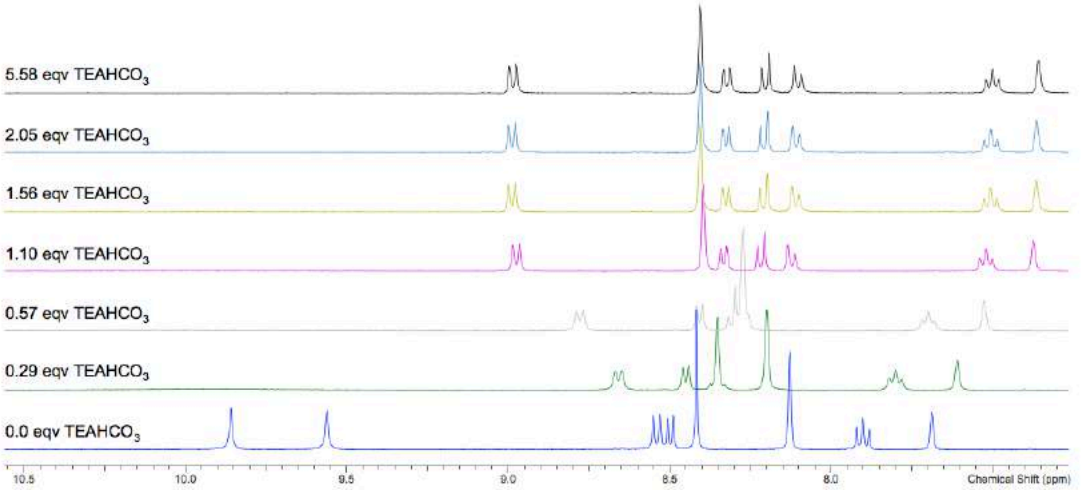

Figure S43: Stack plot showing the aromatic region of the ^{1}H NMR spectrum of **5** during the ^{1}H NMR titration with $TEAHCO_3$ in DMSO-d_6 with 0.5% H_2O (400 MHz, 298 K).

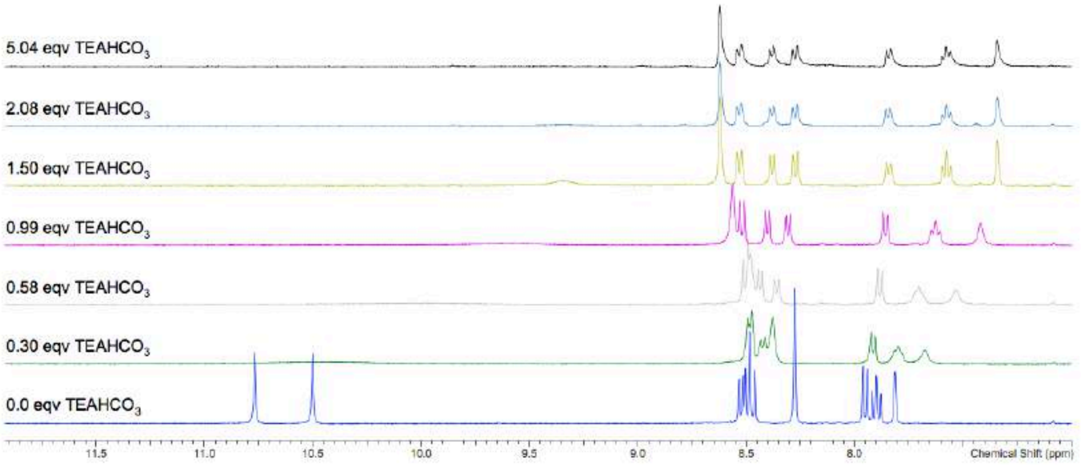

Figure S44: Stack plot showing the aromatic region of the ^{1}H NMR spectrum of **6** during the ^{1}H NMR titration with $TEAHCO_3$ in DMSO-d_6 with 0.5% H_2O (400 MHz, 298 K).

4.4 Interaction with TBAOH

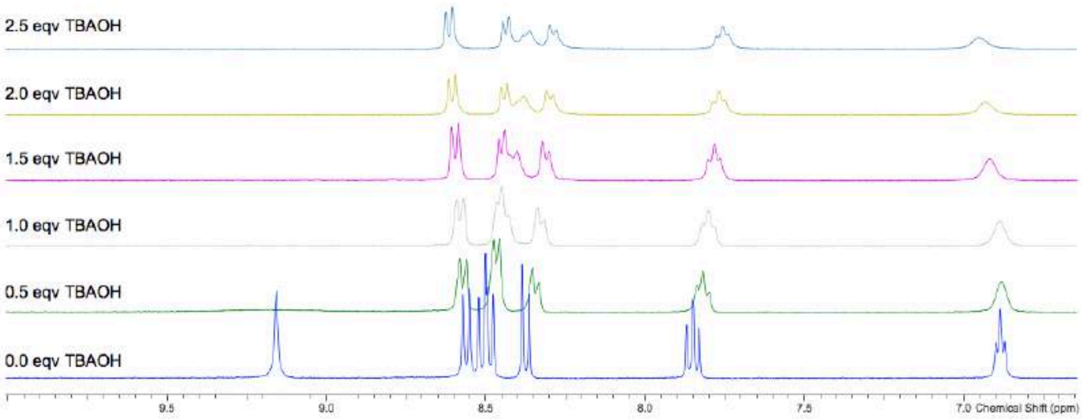

Figure S45: Stack plot showing the aromatic region of the ^{1}H NMR spectrum of **1** during the ^{1}H NMR titration with TBAOH in DMSO-d_6 with 0.5% H_2O (400 MHz, 298 K).

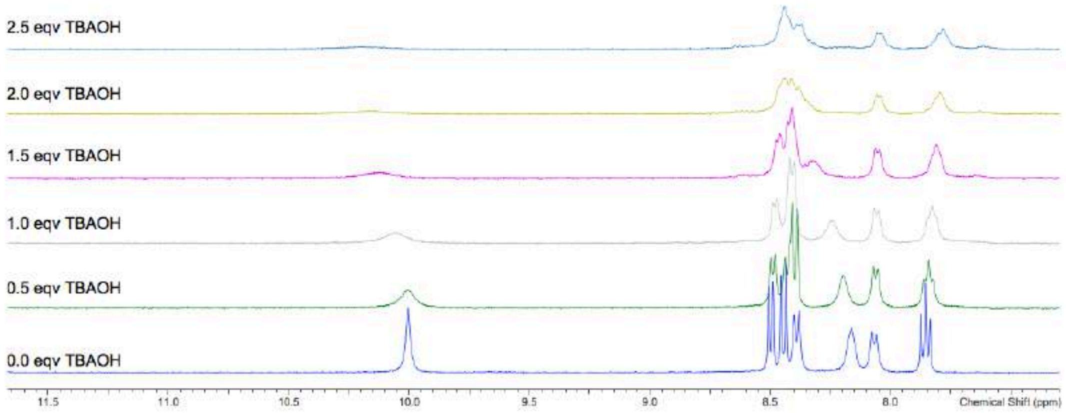

Figure S46: Stack plot showing the aromatic region of the ^{1}H NMR spectrum of **2** during the ^{1}H NMR titration with TBAOH in DMSO-d_6 with 0.5% H_2O (400 MHz, 298 K).

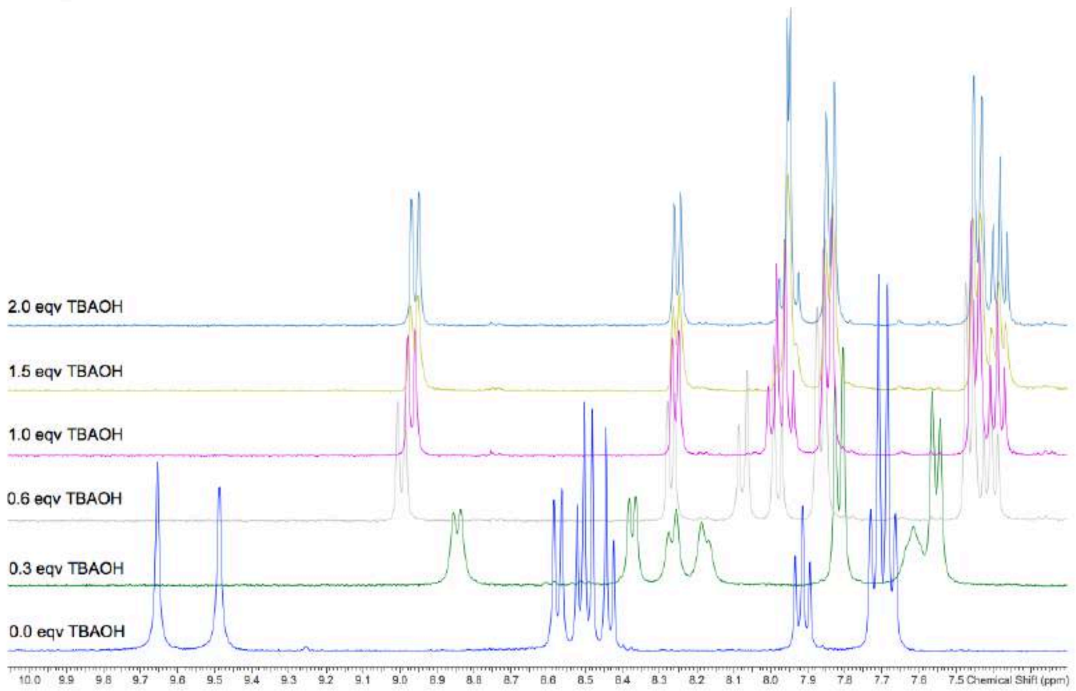

Figure S47: Stack plot showing the aromatic region of the ^{1}H NMR spectrum of **3** during the ^{1}H NMR titration with TBAOH in DMSO-d_6 with 0.5% H_2O (400 MHz, 298 K).

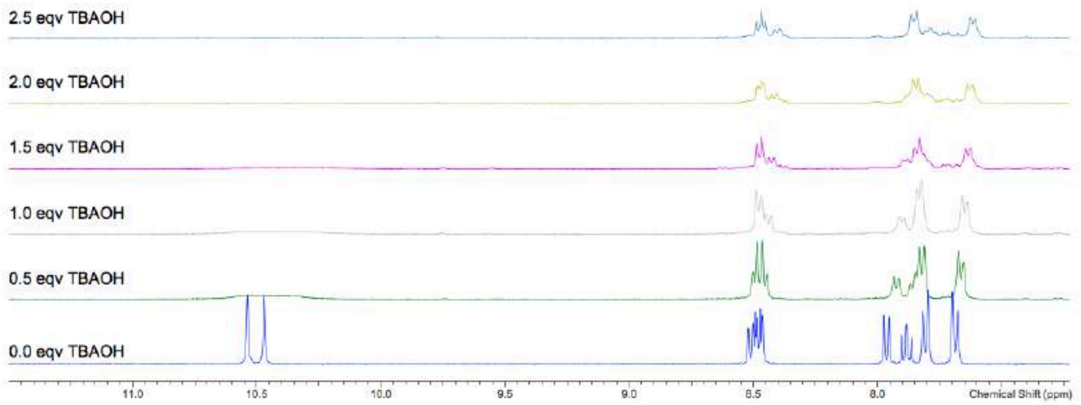

Figure S48: Stack plot showing the aromatic region of the ^{1}H NMR spectrum of **4** during the ^{1}H NMR titration with TBAOH in DMSO-d_6 with 0.5% H_2O (400 MHz, 298 K).

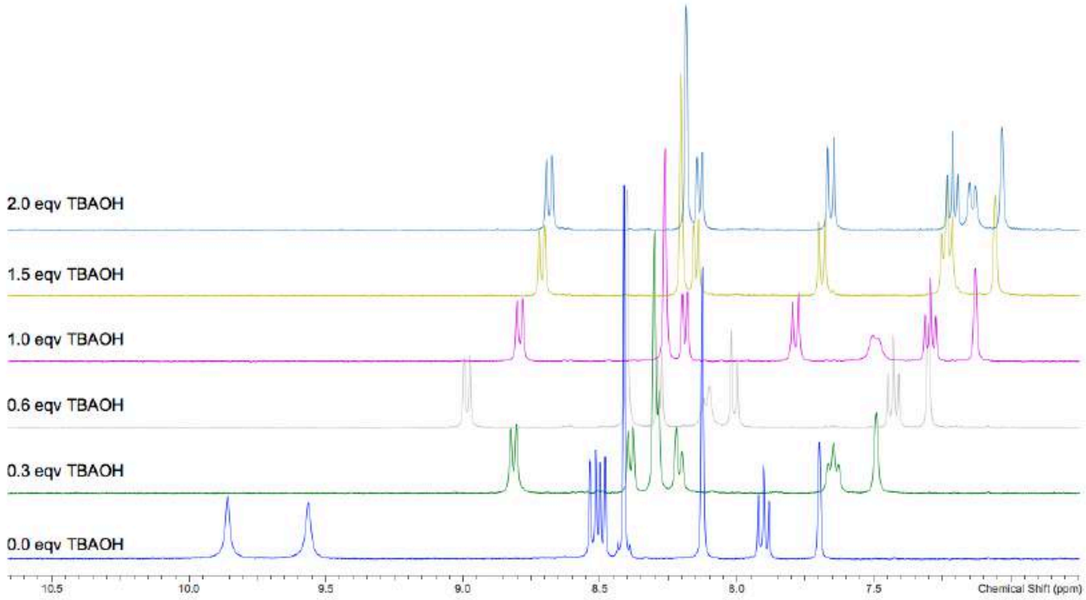

Figure S49: Stack plot showing the aromatic region of the ^{1}H NMR spectrum of **5** during the ^{1}H NMR titration with TBAOH in DMSO-d_6 with 0.5% H_2O (400 MHz, 298 K).

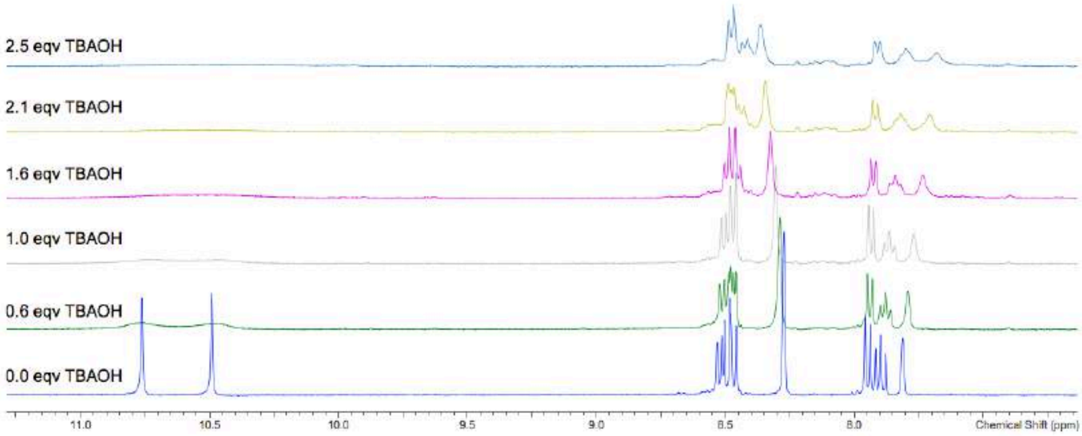

Figure S50: Stack plot showing the aromatic region of the ^{1}H NMR spectrum of **6** during the ^{1}H NMR titration with TBAOH in DMSO-d_6 with 0.5% H_2O (400 MHz, 298 K).

4.5 Interaction with $TBAH_2PO_4$

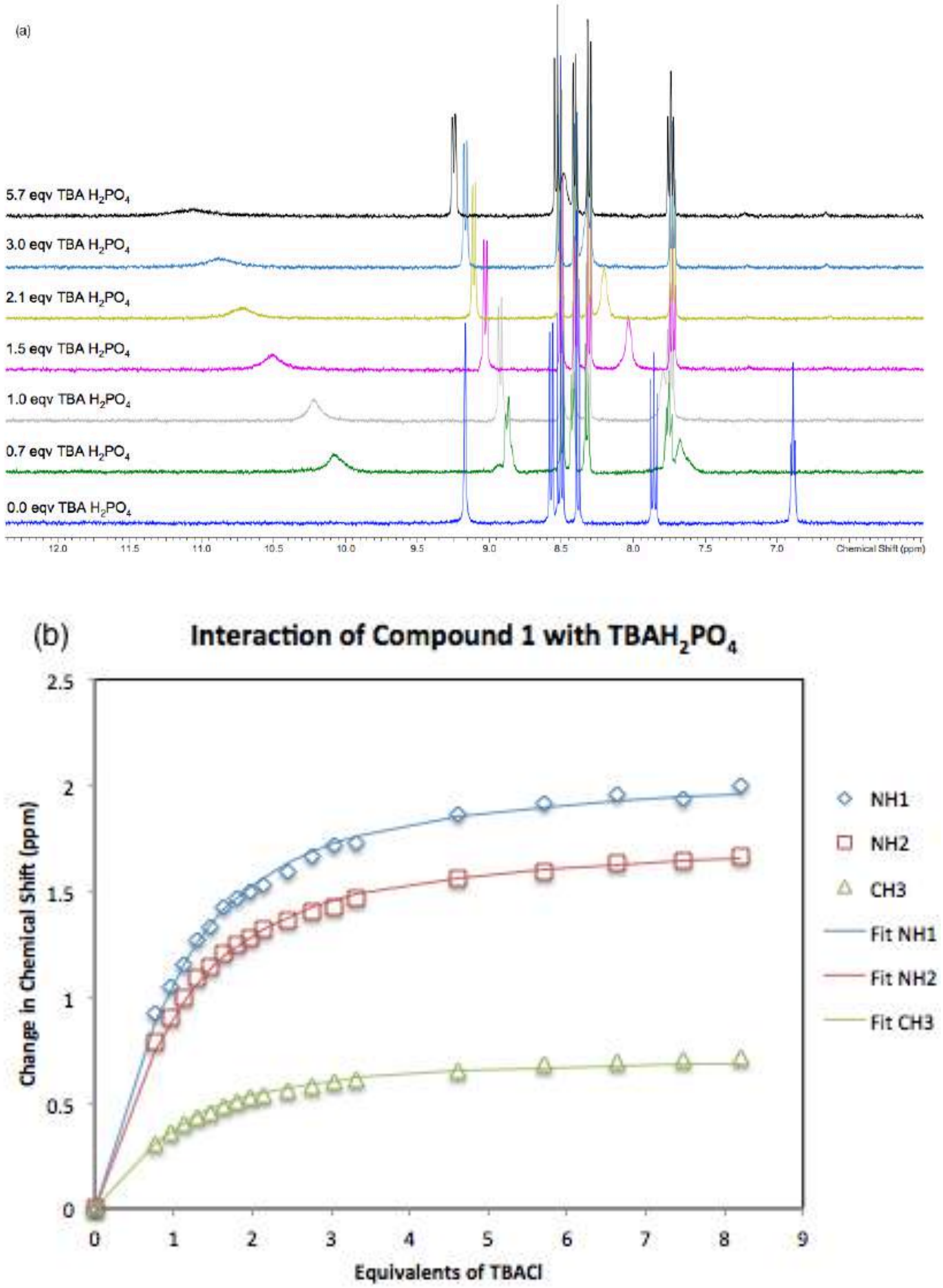

Figure S51: ^{1}H NMR titration of compound **1** with $TBAH_2PO_4$ in DMSO-d_6 with 0.5% H_2O (400 MHz, 298 K). (a) Stack plot showing the aromatic region of **1**. (b) Change in chemical shifts of NH1, NH2 and CH3 during the course of the titration. Also shown are the fitted binding isotherms. K_a = 429 M^{-1}, covariance of the fit = 0.001123.

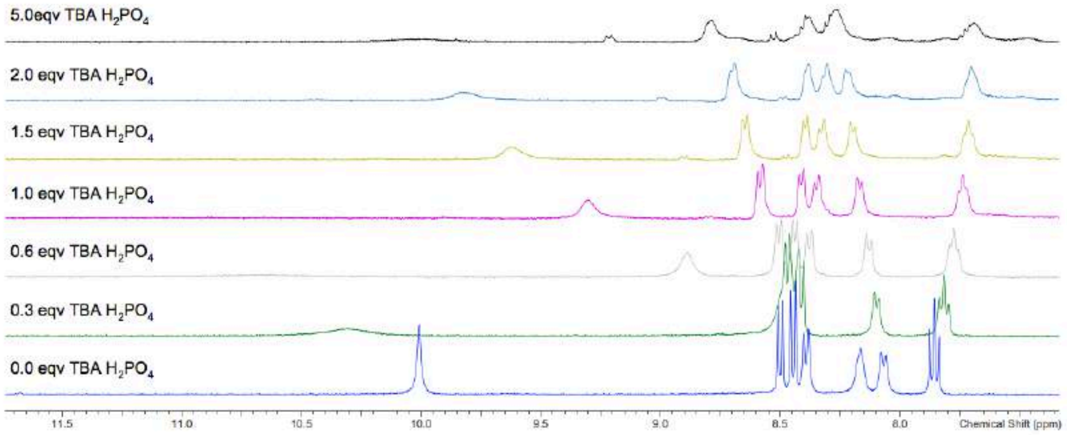

Figure S52: Stack plot showing the aromatic region of the ^{1}H NMR spectrum of **2** during the ^{1}H NMR titration with $TBAH_2PO_4$ in DMSO-d_6 with 0.5% H_2O (400 MHz, 298 K).

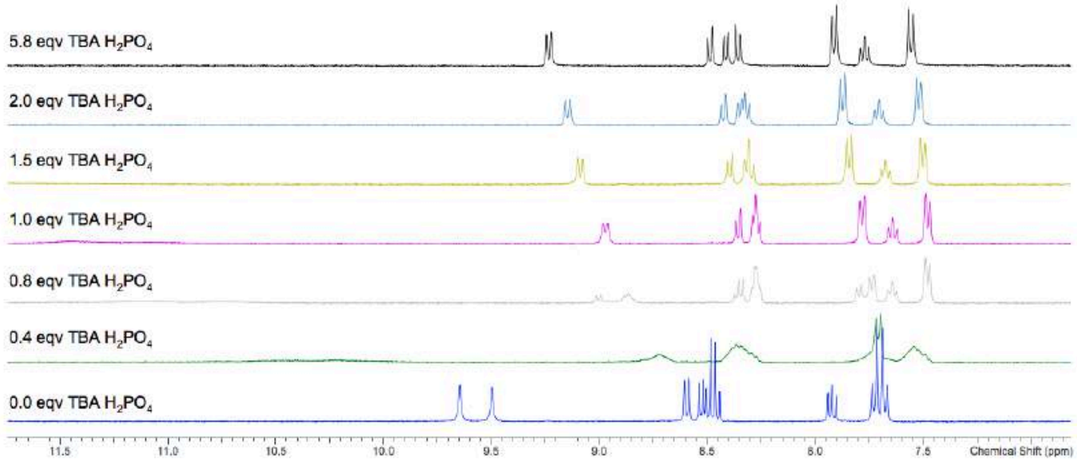

Figure S53: Stack plot showing the aromatic region of the ^{1}H NMR spectrum of **3** during the ^{1}H NMR titration with $TBAH_2PO_4$ in DMSO-d_6 with 0.5% H_2O (400 MHz, 298 K).

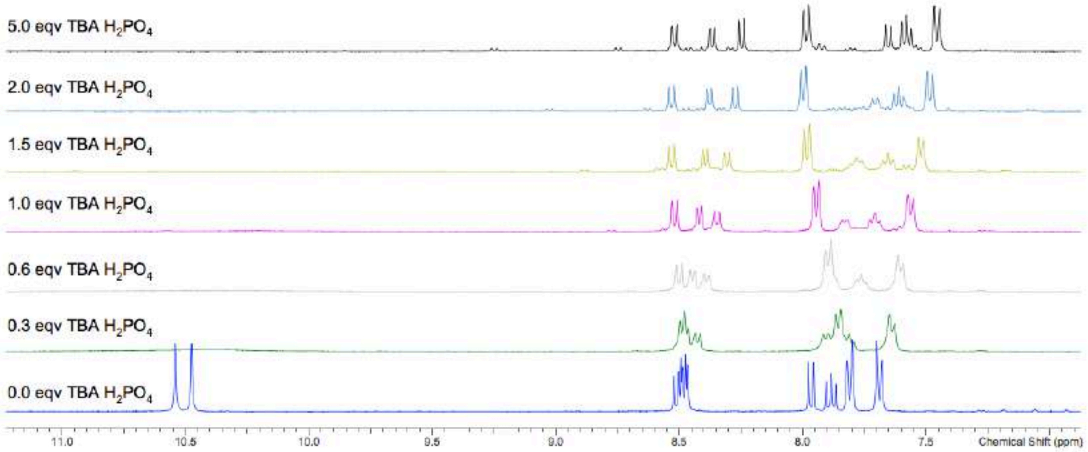

Figure S54: Stack plot showing the aromatic region of the ^{1}H NMR spectrum of **4** during the ^{1}H NMR titration with $TBAH_2PO_4$ in DMSO-d_6 with 0.5% H_2O (400 MHz, 298 K).

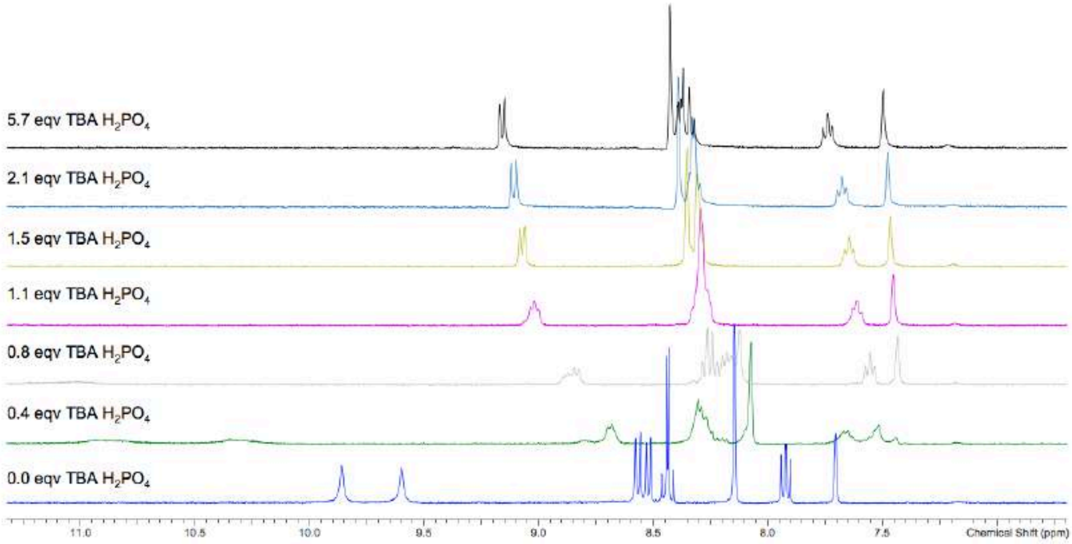

Figure S55: Stack plot showing the aromatic region of the ^{1}H NMR spectrum of **5** during the ^{1}H NMR titration with $TBAH_2PO_4$ in DMSO-d_6 with 0.5% H_2O (400 MHz, 298 K).

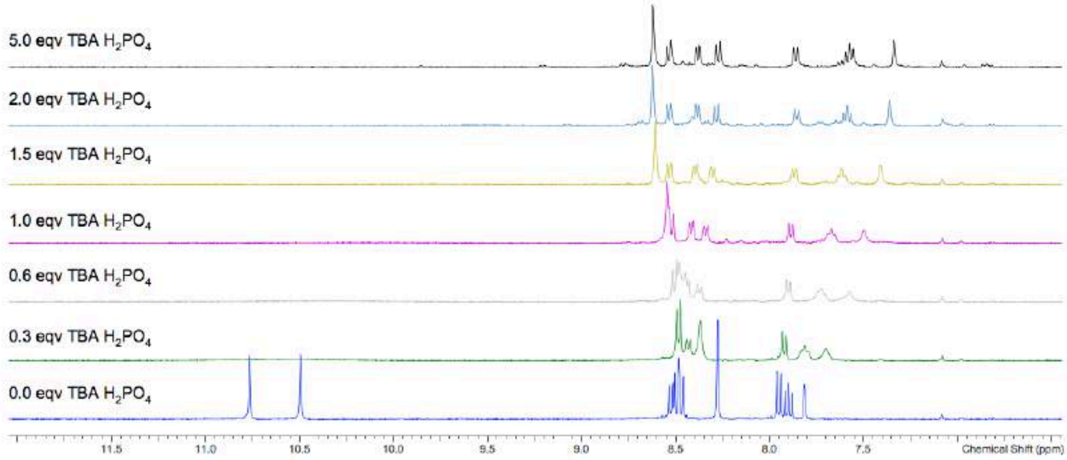

Figure S56: Stack plot showing the aromatic region of the ^{1}H NMR spectrum of **6** during the ^{1}H NMR titration with $TBAH_2PO_4$ in DMSO-d_6 with 0.5% H_2O (400 MHz, 298 K).

4.6 Interaction with $TBANO_3$

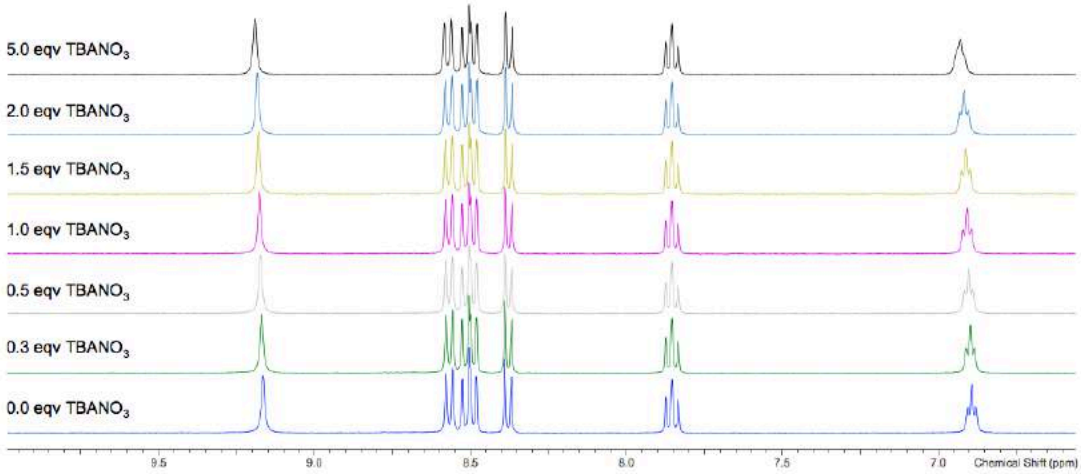

Figure S57: Stack plot showing the aromatic region of the ^{1}H NMR spectrum of **1** during the ^{1}H NMR titration with $TBANO_3$ in DMSO-d_6 with 0.5% H_2O (400 MHz, 298 K).

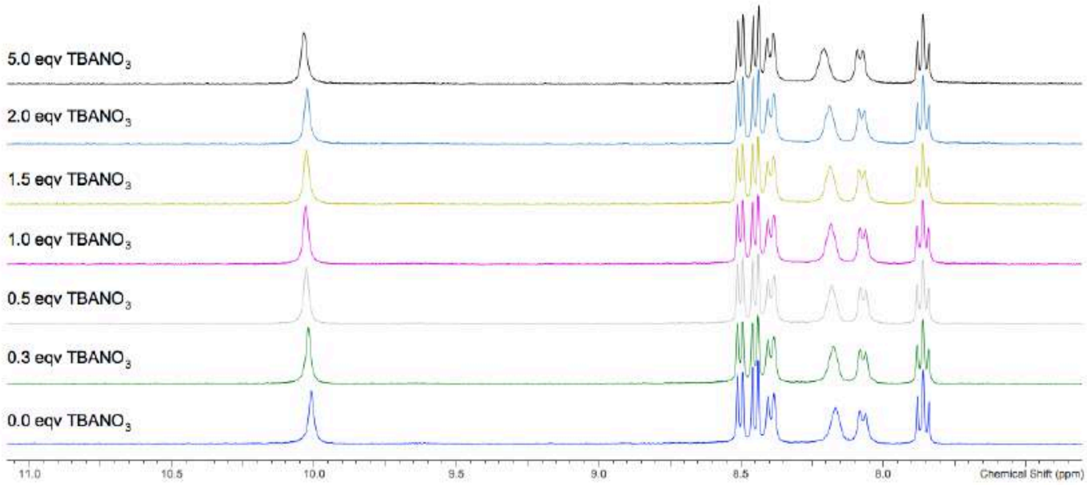

Figure S58: Stack plot showing the aromatic region of the ^{1}H NMR spectrum of **2** during the ^{1}H NMR titration with $TBANO_3$ in DMSO-d_6 with 0.5% H_2O (400 MHz, 298 K).

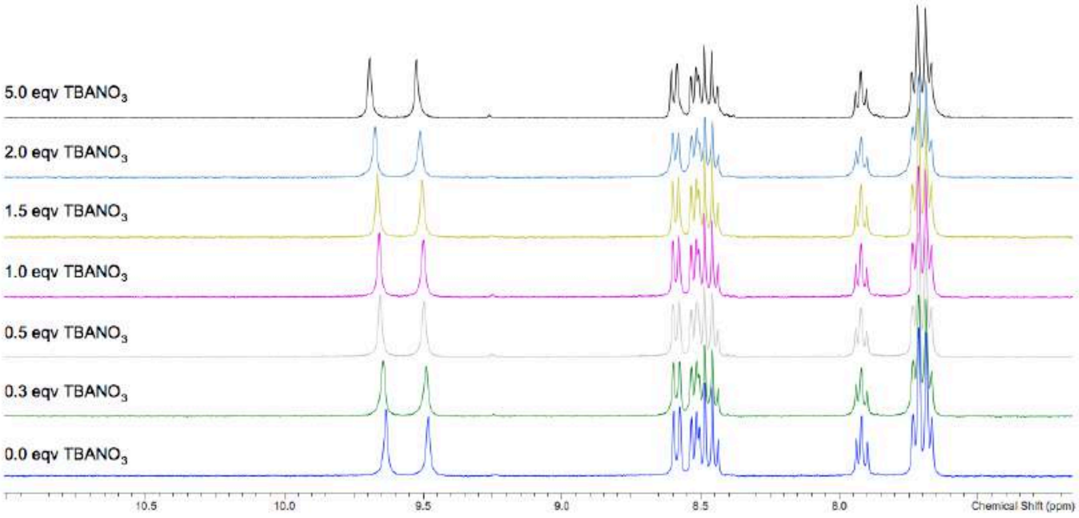

Figure S59: Stack plot showing the aromatic region of the ^{1}H NMR spectrum of **3** during the ^{1}H NMR titration with $TBANO_3$ in DMSO-d_6 with 0.5% H_2O (400 MHz, 298 K).

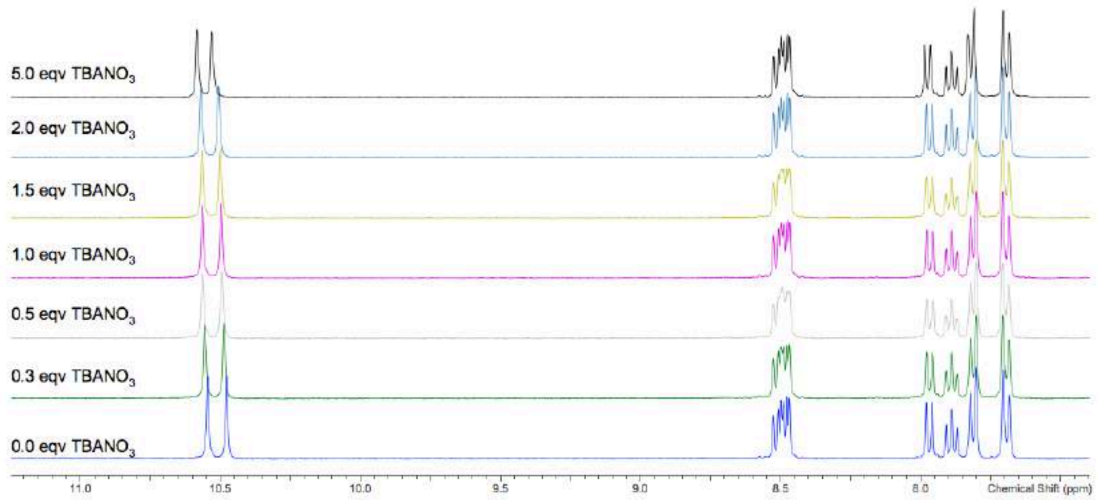

Figure S60: Stack plot showing the aromatic region of the ^{1}H NMR spectrum of **4** during the ^{1}H NMR titration with $TBANO_3$ in DMSO-d_6 with 0.5% H_2O (400 MHz, 298 K).

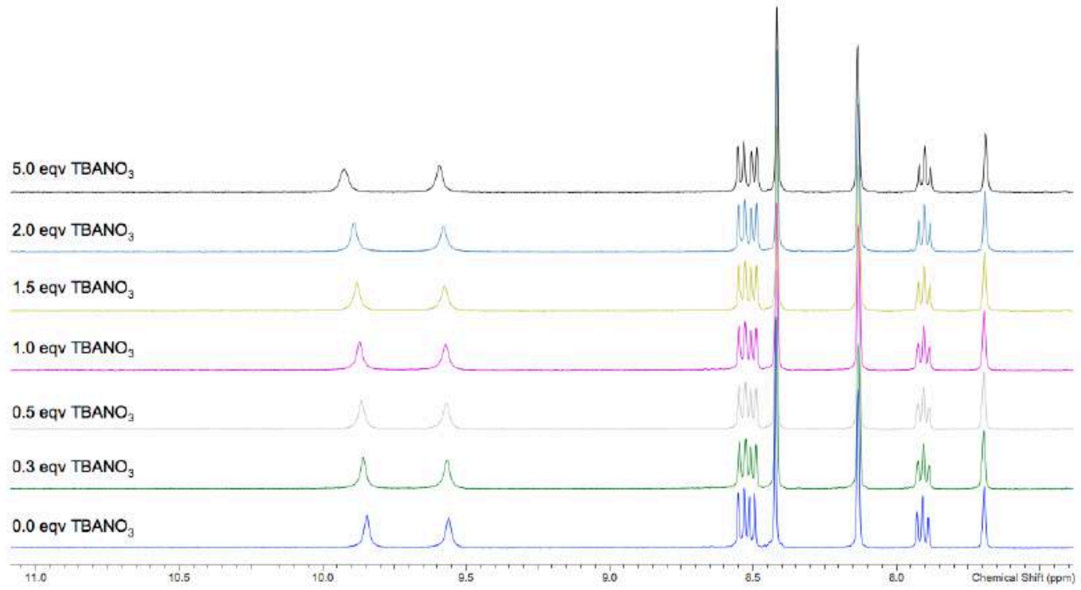

Figure S61: Stack plot showing the aromatic region of the ^{1}H NMR spectrum of **5** during the ^{1}H NMR titration with $TBANO_3$ in DMSO-d_6 with 0.5% H_2O (400 MHz, 298 K).

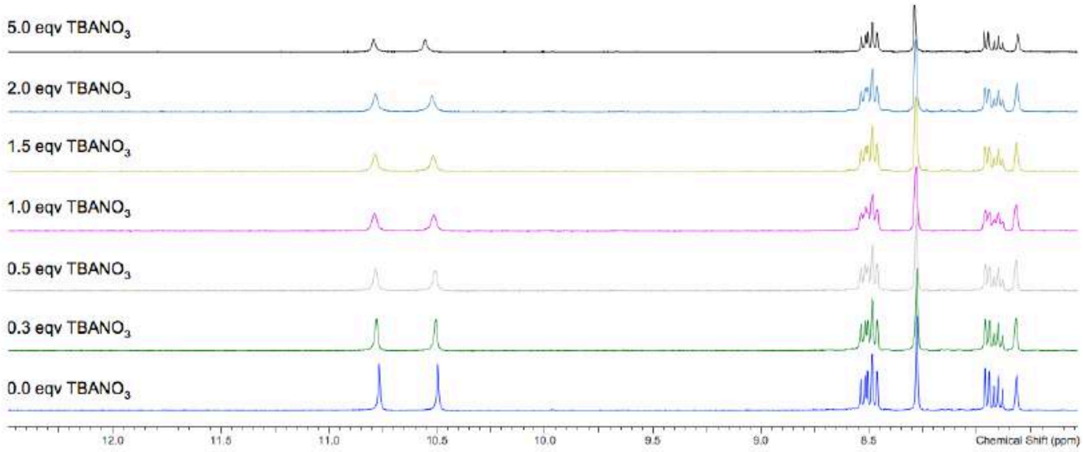

Figure S62: Stack plot showing the aromatic region of the ^{1}H NMR spectrum of **6** during the ^{1}H NMR titration with $TBANO_3$ in DMSO-d_6 with 0.5% H_2O (400 MHz, 298 K).

5 – UV-Vis Studies

5.1 UV-Vis Studies with $TEAHCO_3$

In order to examine the effects of HCO_3^- on **1**-**6** we performed UV-Vis titrations with increasing additions of $TEAHCO_3$ in DMSO.

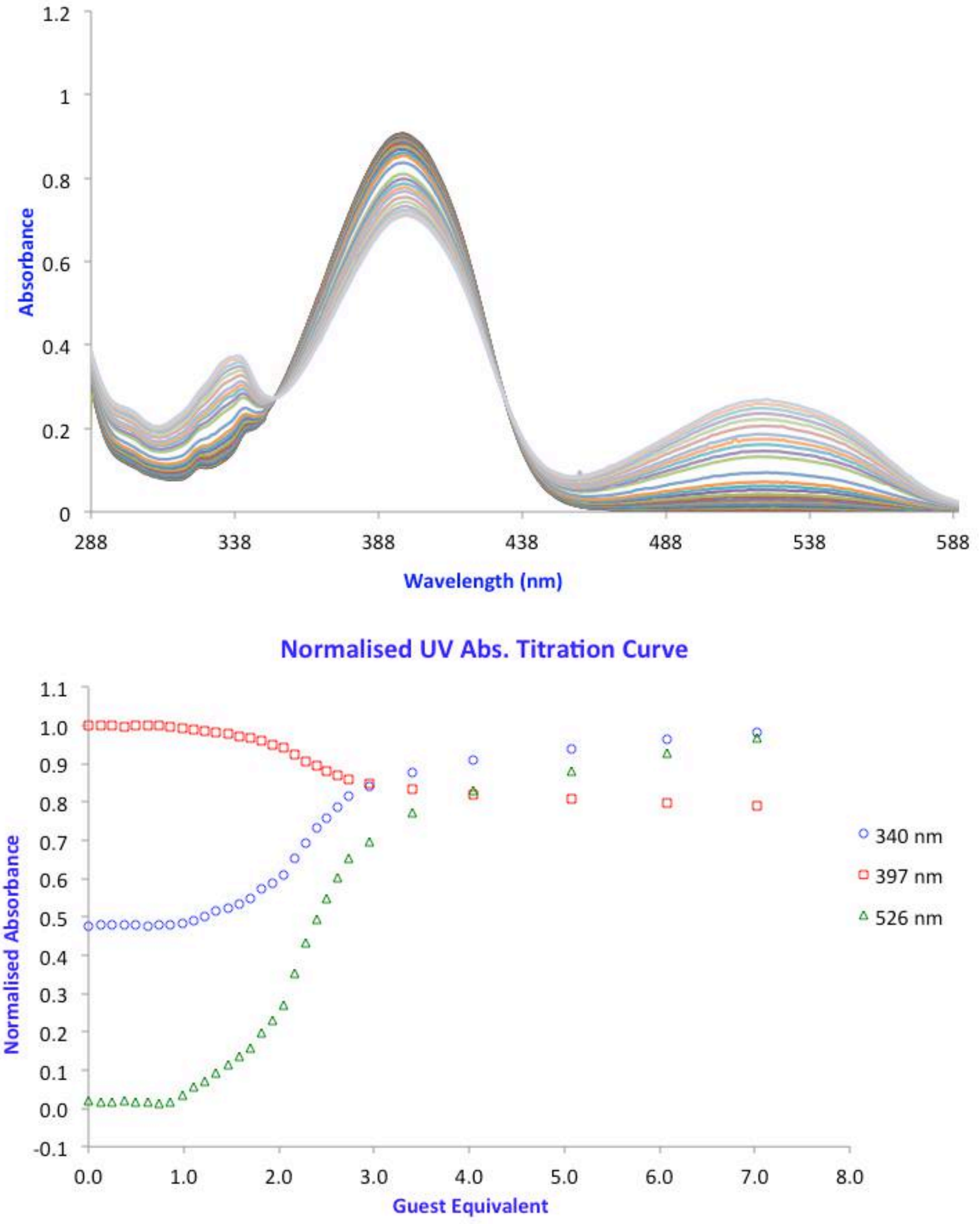

Figure S63: UV-Vis spectra of compound **1** (10 μM) upon increasing additions of $TEAHCO_3$ (above) in DMSO and plot of normalised absorbance versus equivalents of HCO_3^- for the peaks at 340 nm, 397 nm and 526 nm (below).

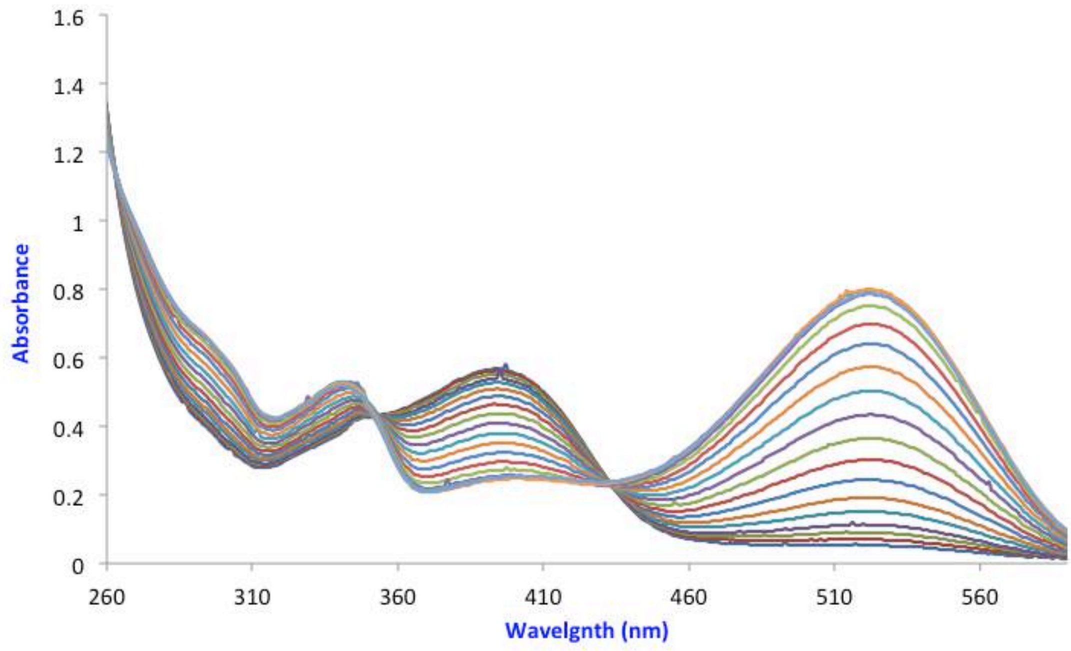

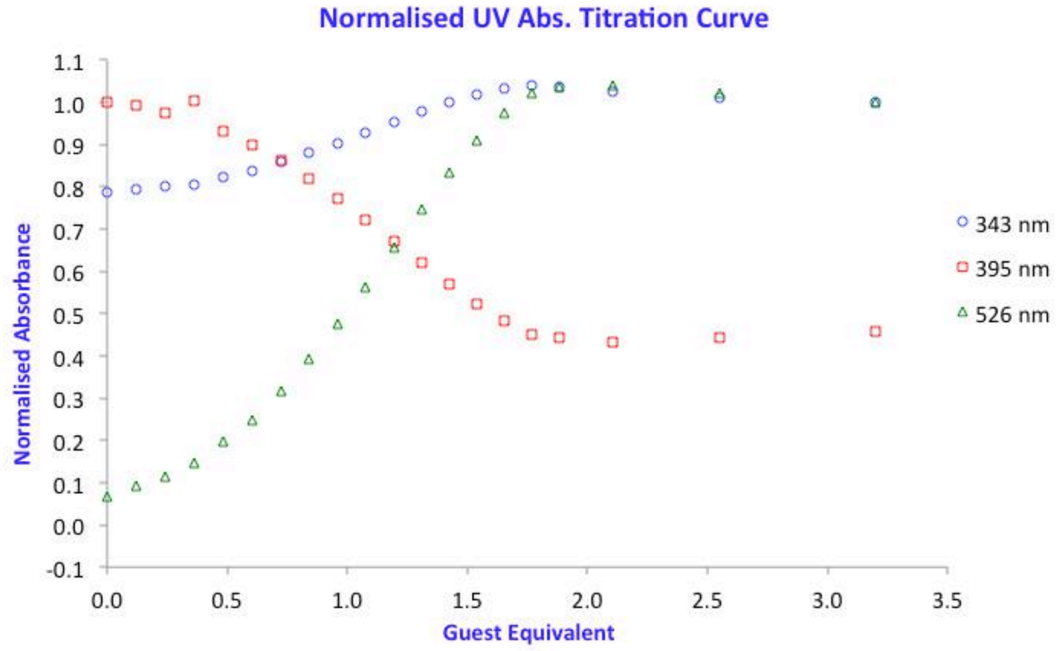

Figure S64: UV-Vis spectra of compound **2** (10 μM) upon increasing additions of $TEAHCO_3$ (above) in DMSO and plot of normalised absorbance versus equivalents of HCO_3^- for the peaks at 343 nm, 395 nm and 526 nm (below).

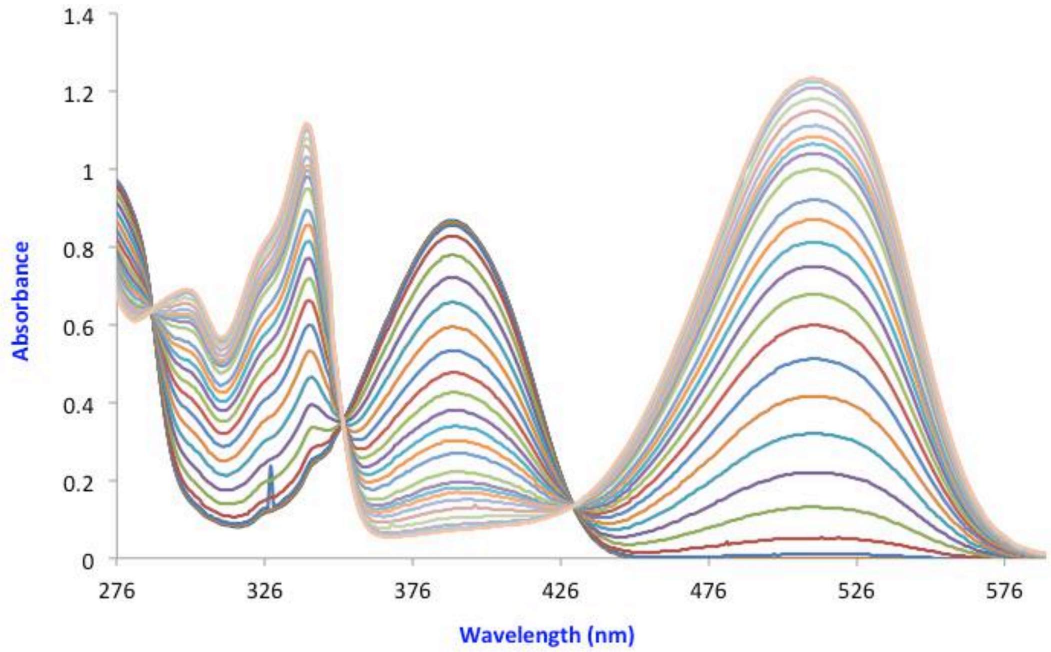

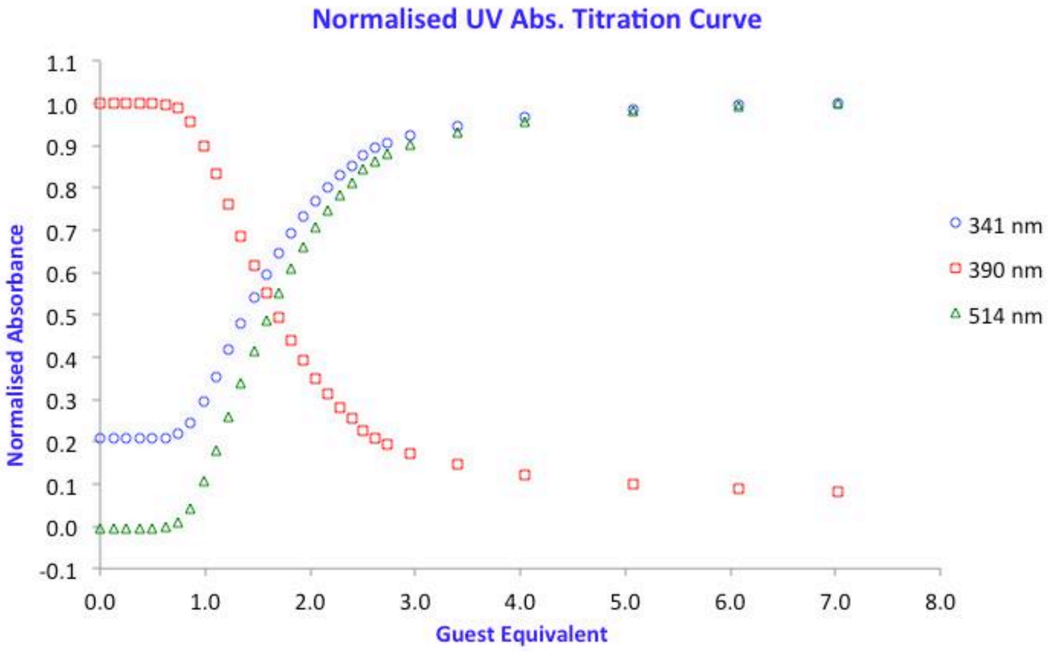

Figure S65 UV-Vis spectra of compound **3** (10 μM) upon increasing additions of $TEAHCO_3$ (above) in DMSO and plot of normalised absorbance versus equivalents of HCO_3^- for the peaks at 341 nm, 390 nm and 514 nm (below).

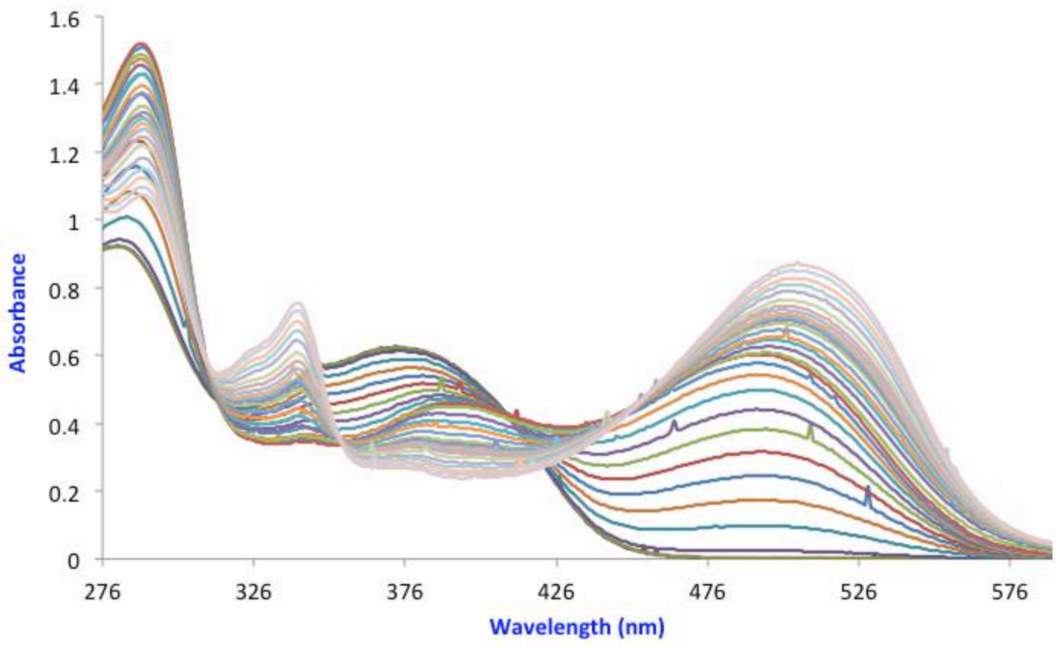

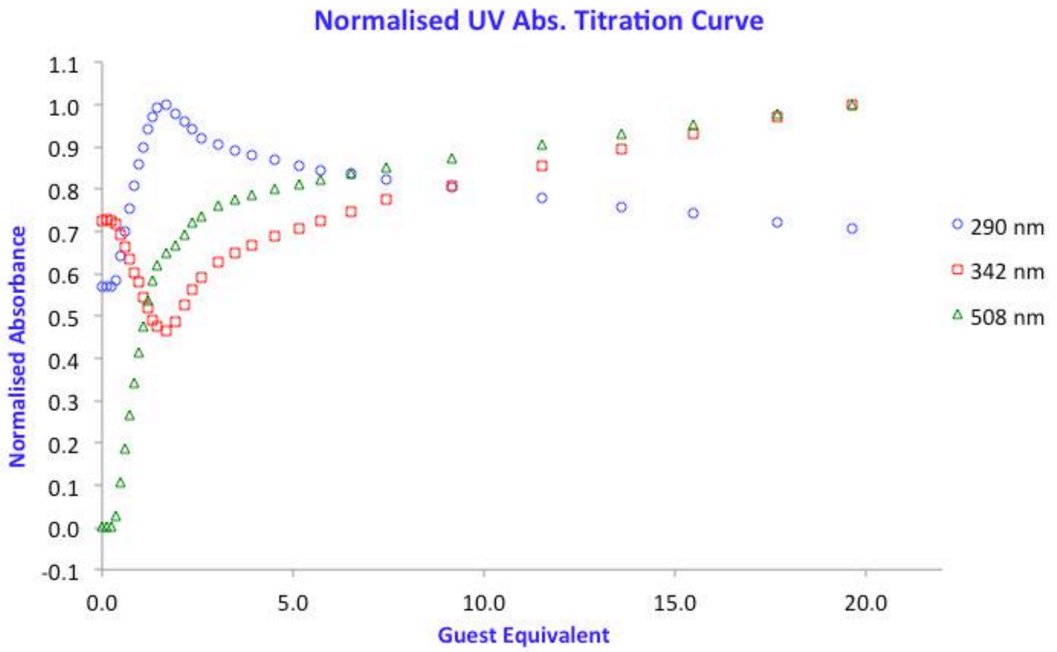

Figure S66 UV-Vis spectra of compound **4** (10 μM) upon increasing additions of $TEAHCO_3$ (above) in DMSO and plot of normalised absorbance versus equivalents of HCO_3^- for the peaks at 290 nm, 342 nm and 508 nm (below).

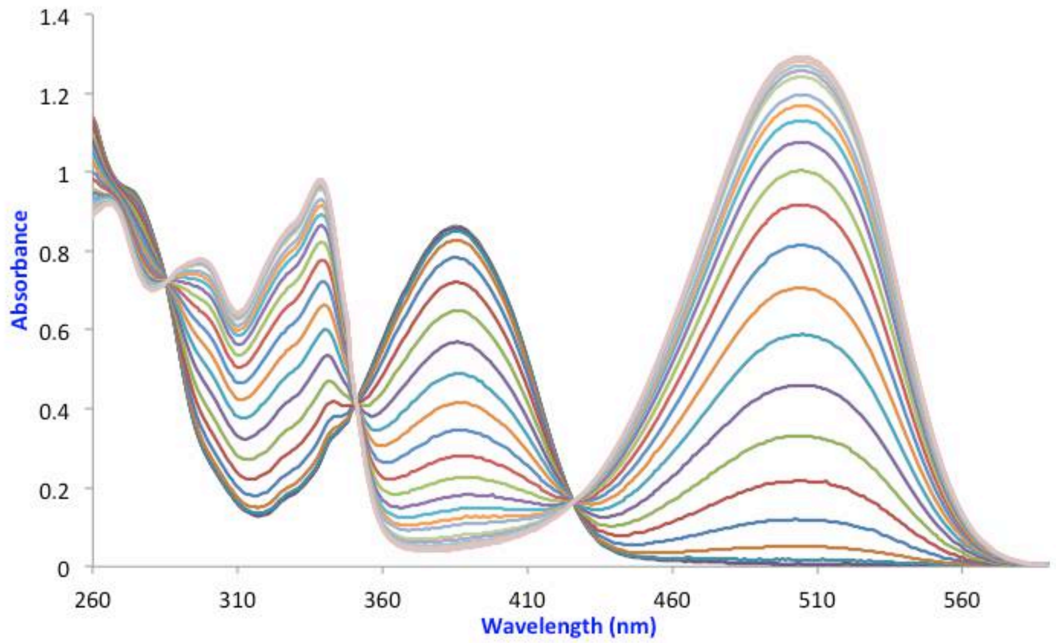

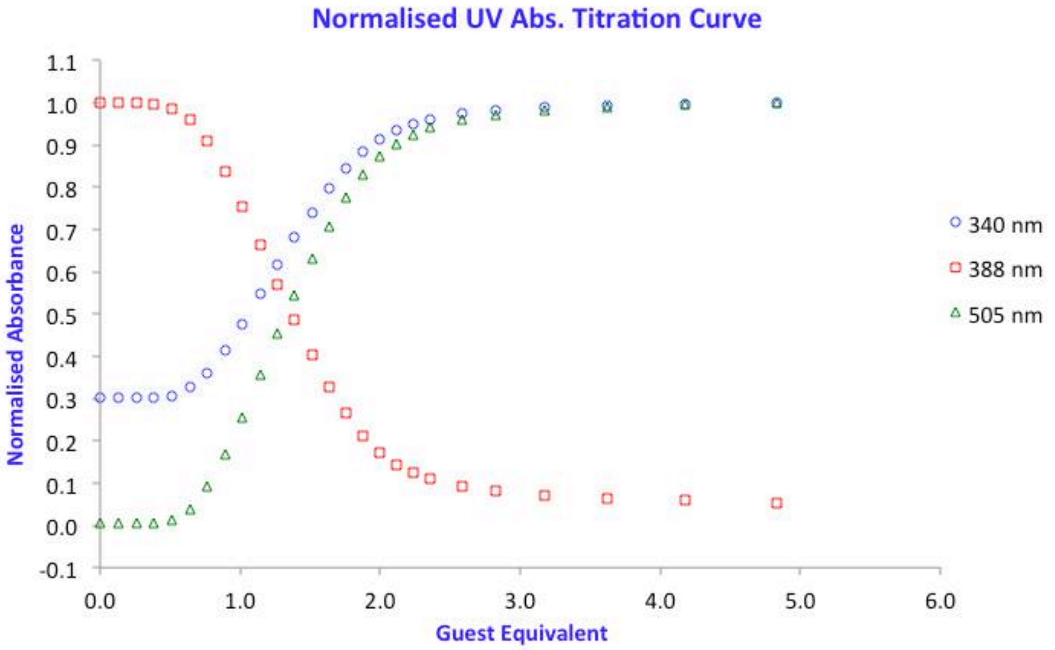

Figure S67 UV-Vis spectra of compound **5** (10 μM) upon increasing additions of TEAHCO$_3$ (top) in DMSO and plot of normalised absorbance versus equivalents of HCO_3^- for the peaks at 340 nm, 388 nm and 505 nm (bottom).

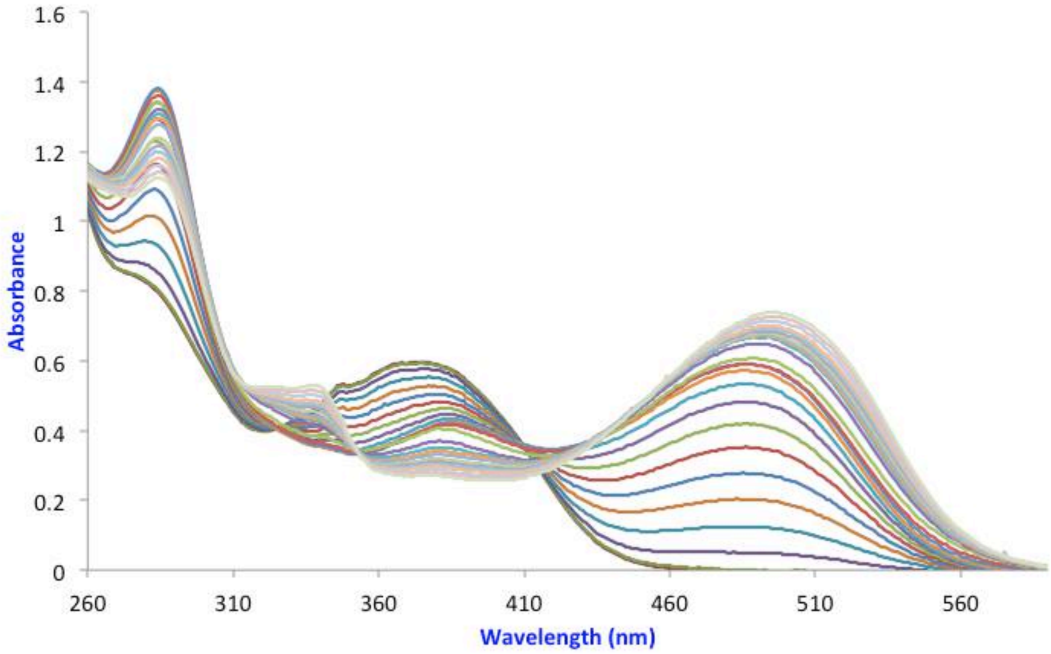

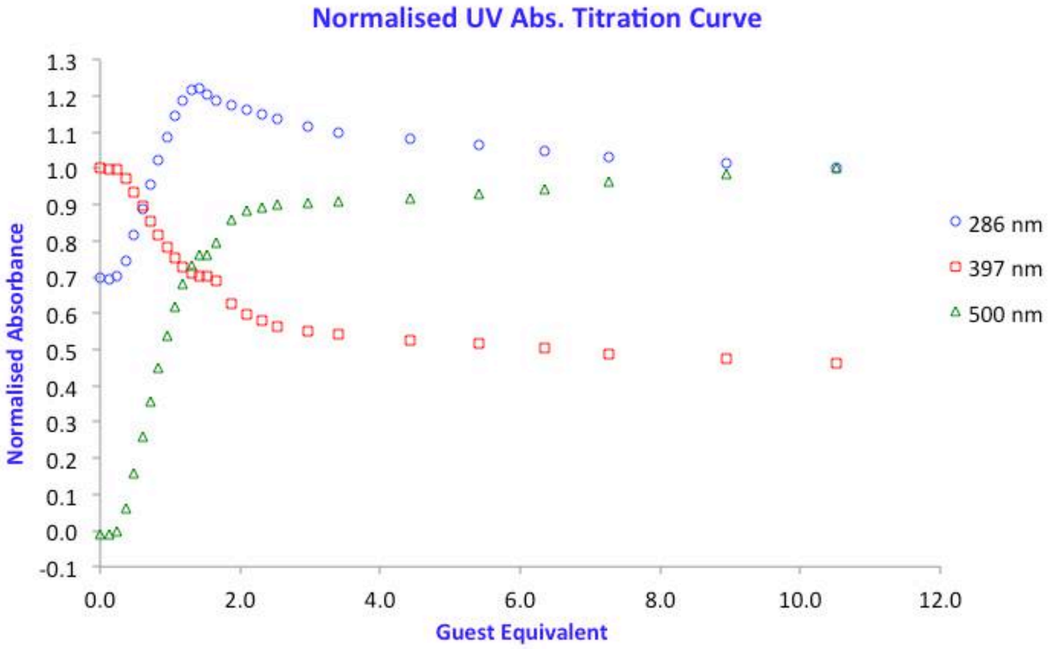

Figure S68: UV-Vis spectra of compound **6** (10 μM) upon increasing additions of $TEAHCO_3$ (top) in DMSO and plot of normalised absorbance versus equivalents of HCO_3^- for the peaks at 286 nm, 397 nm and 500 nm (bottom).

5.2 UV-Vis studies in varying pH

pH studies were performed using a method adapted from Fabbrizzi *et al.*[5] Compounds **1**-**6** were dissolved in DMSO:H_2O (9:1) containing 100 mM $TBAPF_6$ to maintain ionic strength (10 μM compound concentration). The solutions were acidified with a small aliquot of nitric acid and slowly basified by adding small amounts of 0.1M NaOH.

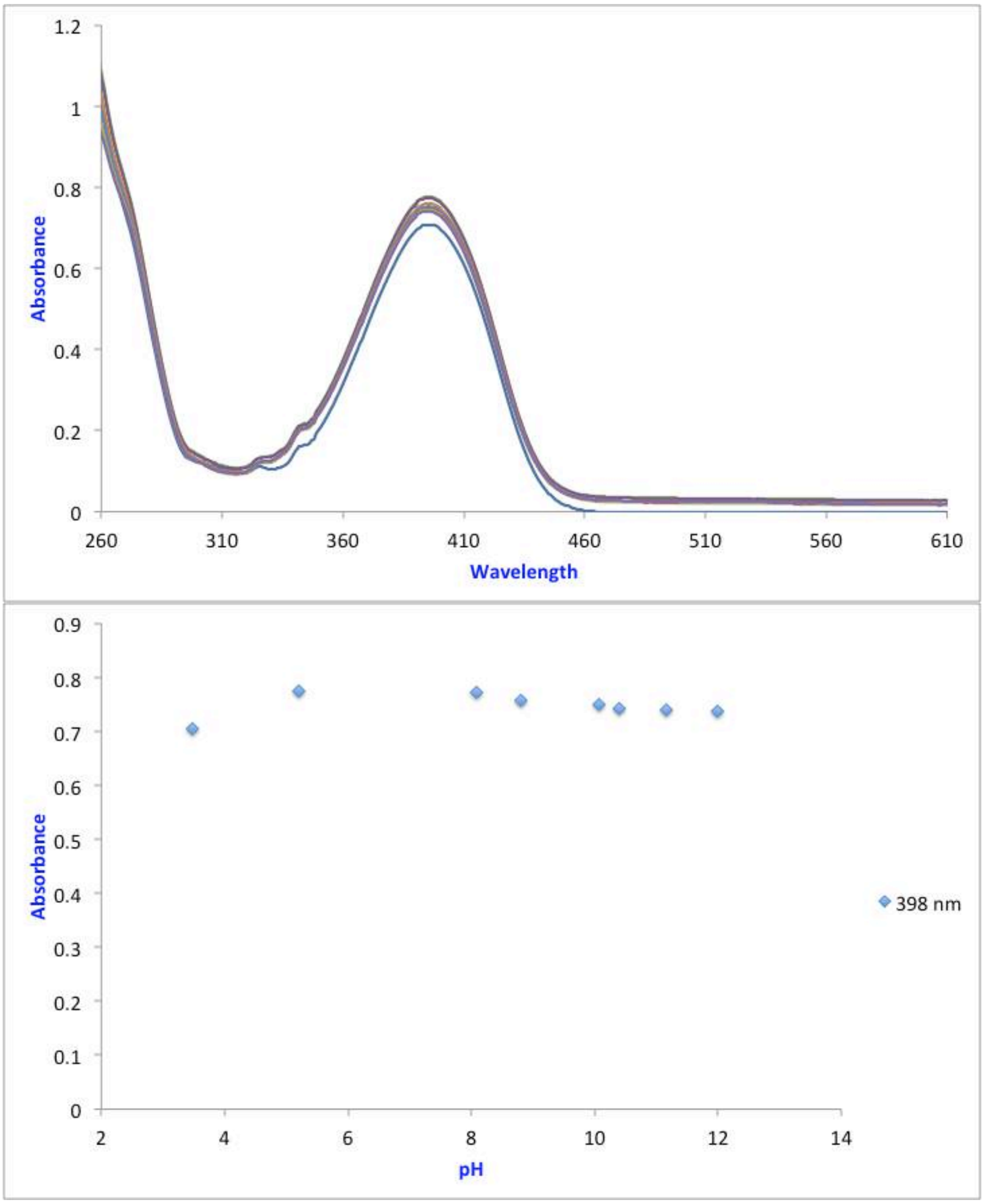

Figure S69: UV-Vis spectra of compound **1** (10 μM) in 9:1 DMSO:H_2O containing 100 mM $TBAPF_6$ in varying pH (above). Plot of absorbance versus pH of the peak at 398 nm (below).

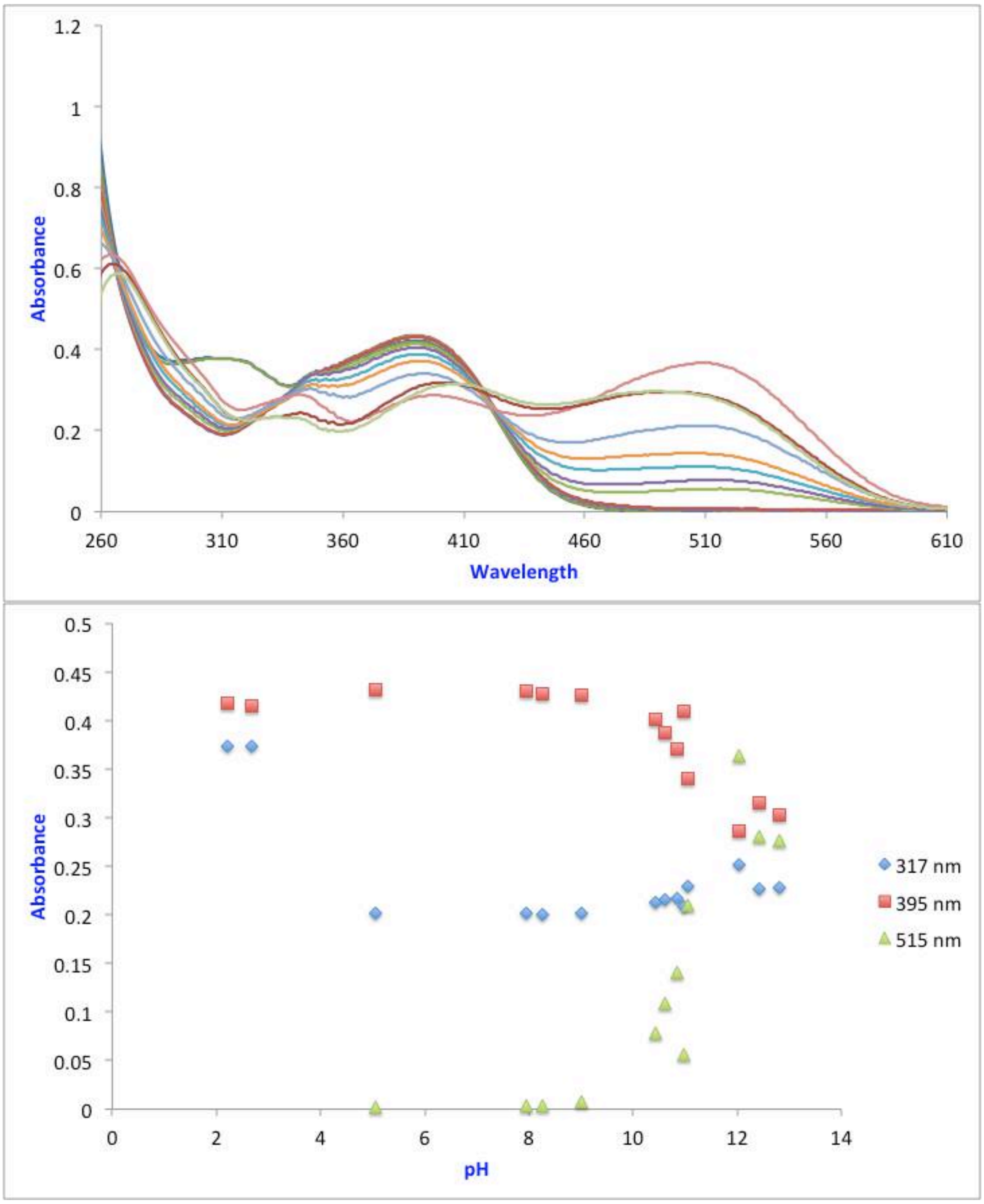

Figure S70: UV-Vis spectra of compound **2** (10 μM) in 9:1 DMSO:H_2O containing 100 mM $TBAPF_6$ at varying pH (top). Plot of absorbance versus pH of the peaks at 317 nm, 395 nm and 515 nm (bottom).

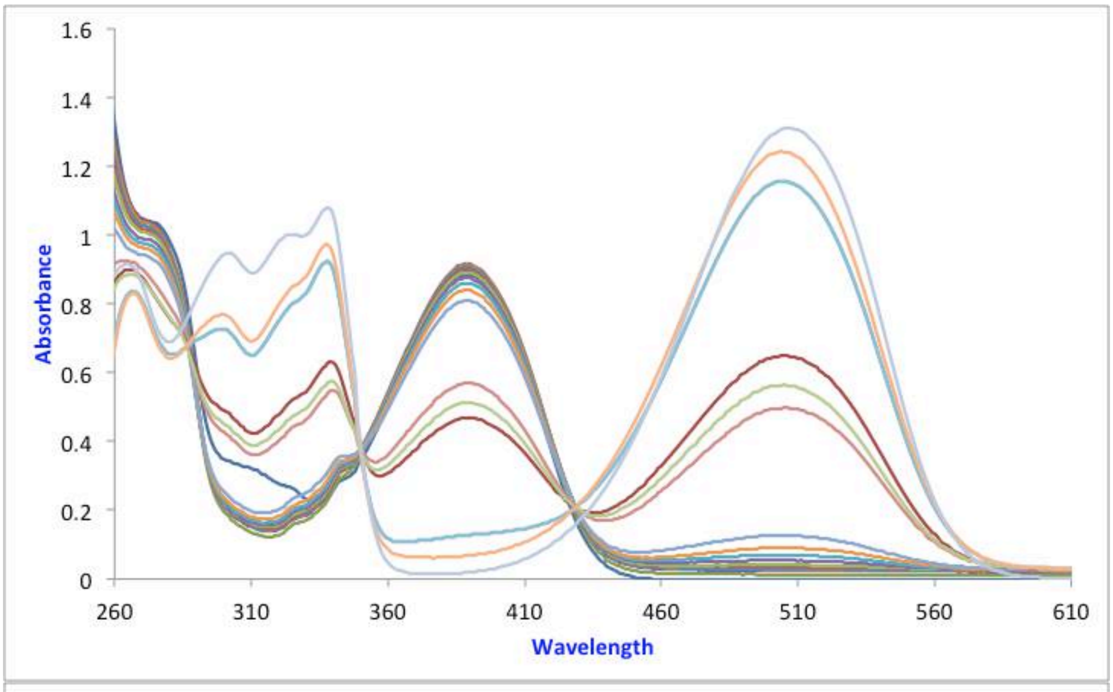

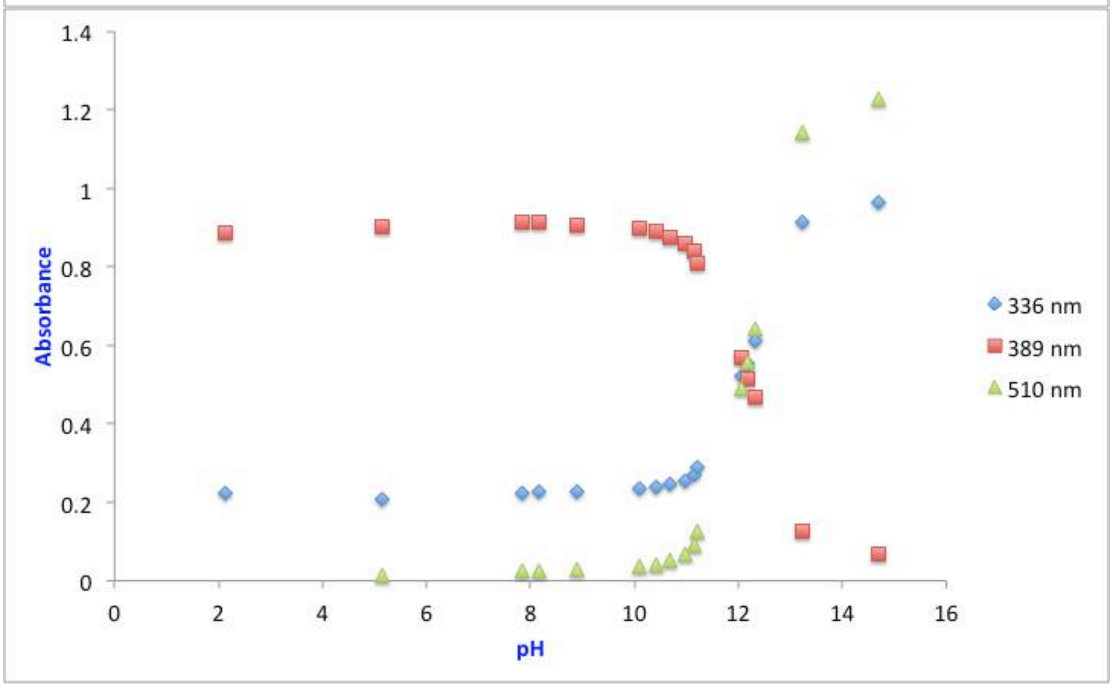

Figure S71 UV-Vis spectra of compound **3** (10 μM) in 9:1 DMSO:H_2O containing 100 mM $TBAPF_6$ in varying pH (above). Plot of absorbance versus pH of the peaks at 336 nm, 389 nm and 510 nm (below).

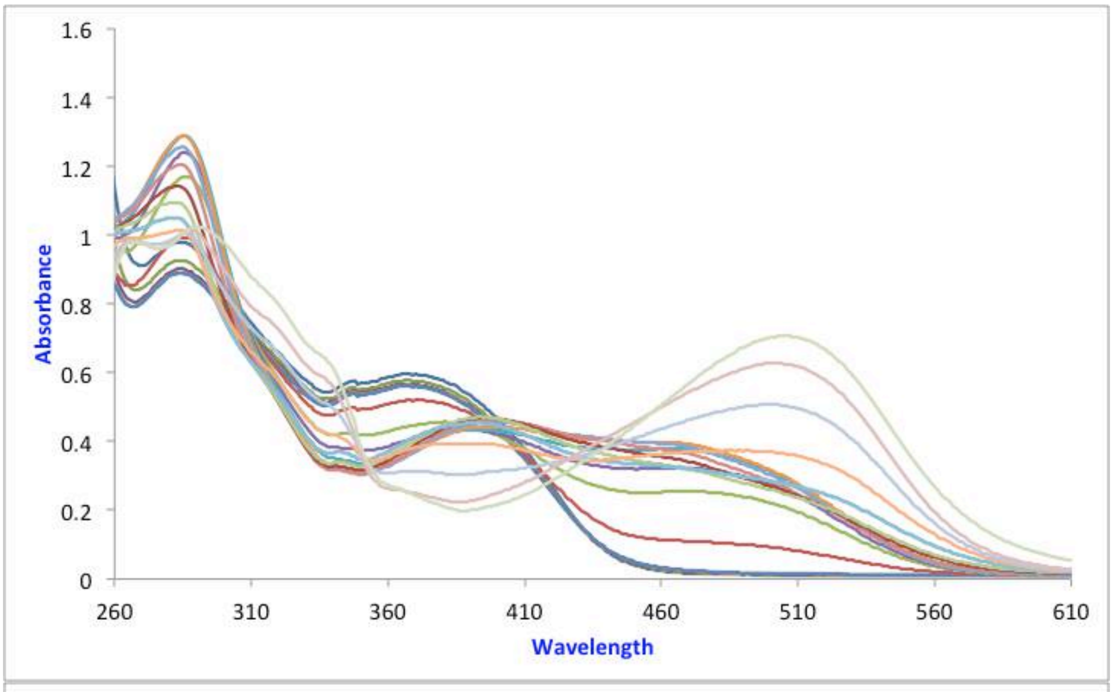

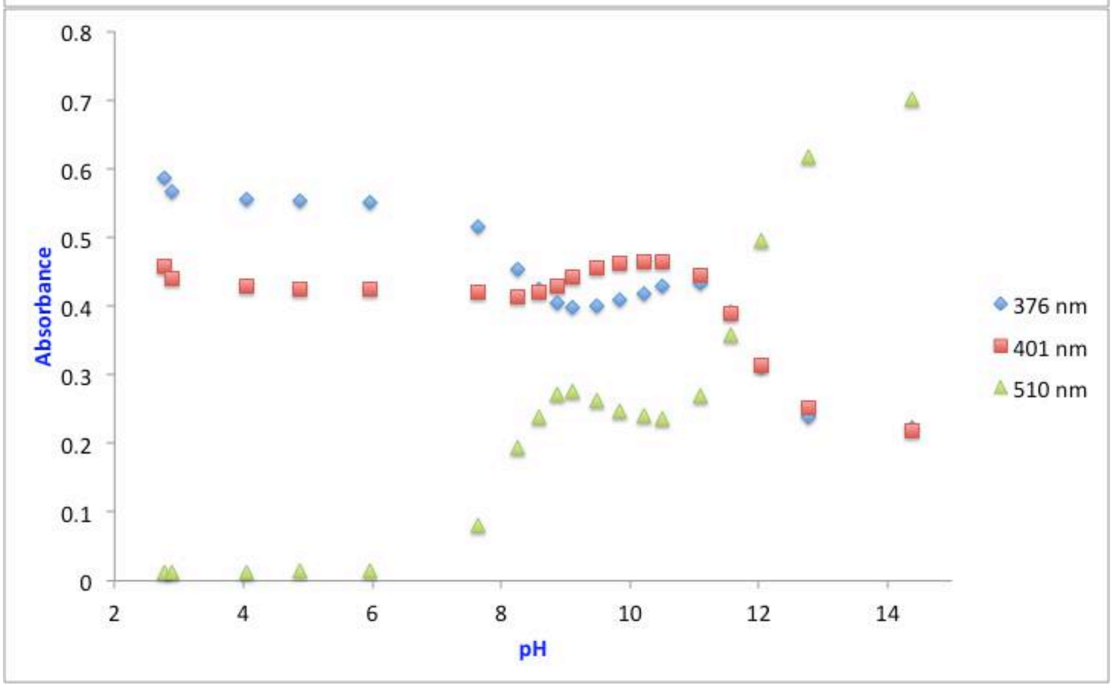

Figure S72 UV-Vis spectra of compound **4** (10 μM) in 9:1 DMSO:H_2O containing 100 mM $TBAPF_6$ in varying pH (above). Plot of absorbance versus pH of the peaks at 376 nm, 401 nm and 510 nm (below).

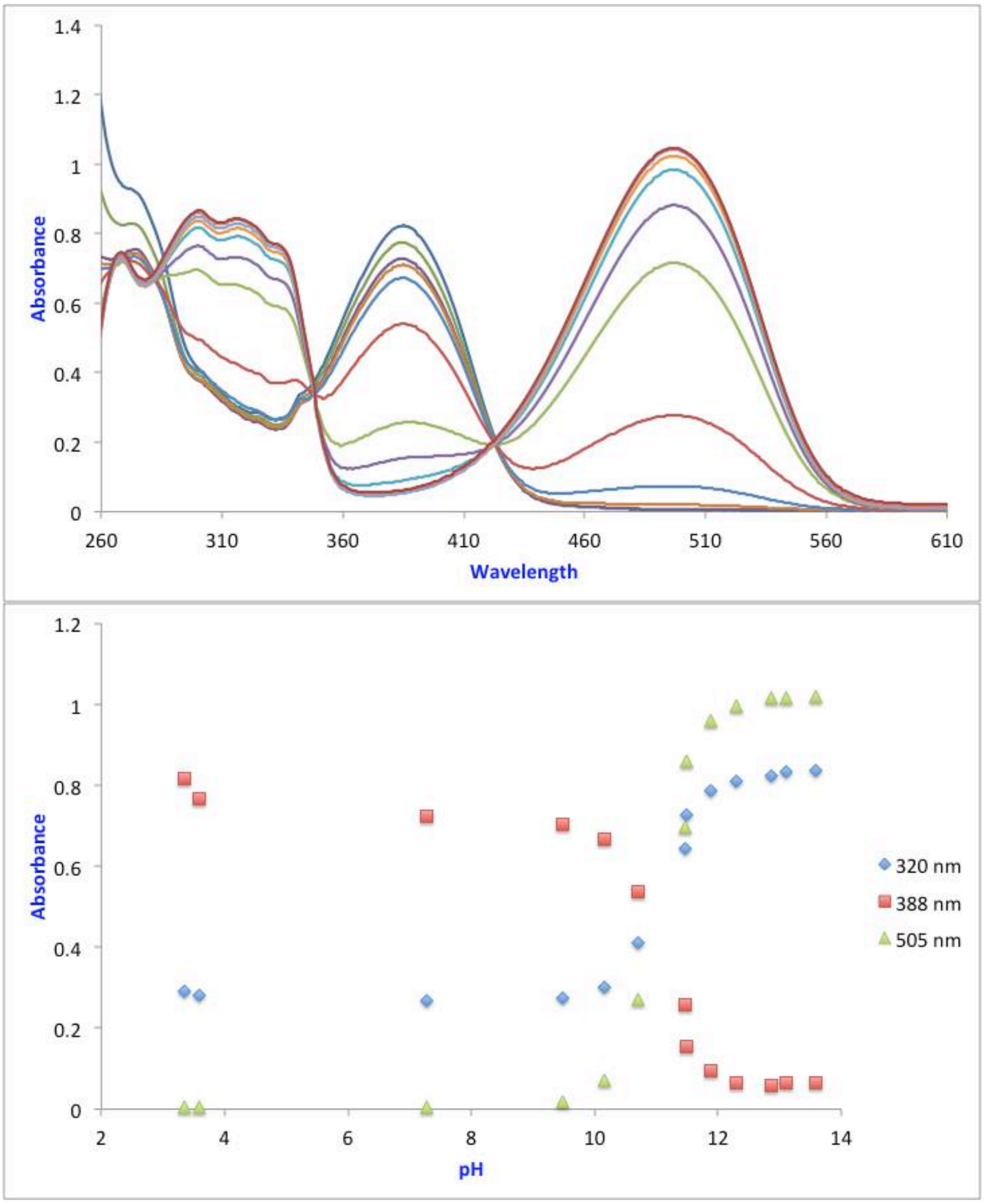

Figure S73 UV-Vis spectra of compound **5** (10 μM) in 9:1 DMSO:H_2O containing 100 mM $TBAPF_6$ in varying pH (above). Plot of absorbance versus pH of the peaks at 320 nm, 388 nm and 505 nm (below).

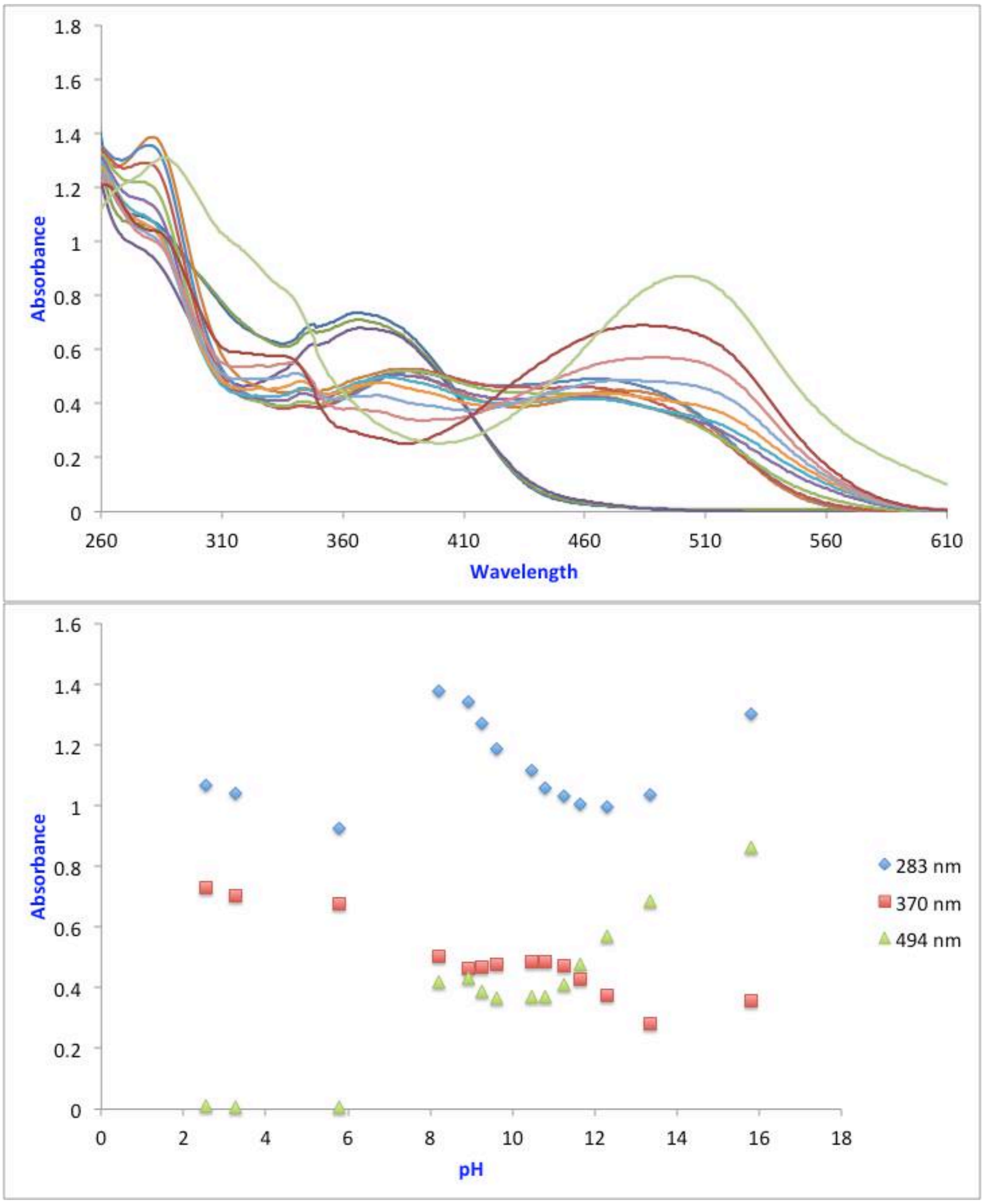

Figure S74 UV-Vis spectra of compound **6** (10 μM) in 9:1 DMSO:H_2O containing 100 mM $TBAPF_6$ in varying pH (above). Plot of absorbance versus pH of the peaks at 283 nm, 370 nm and 494 nm (below).

6 – Additional Fluorescence Studies

6.1 Fluorescence spectra in varying pH

To ascertain whether receptors **1**-**6** were still fluorescent over a pH range we recorded emission spectra in 0.5% DMSO and varying aqueous solutions (10 µM receptor concentration). In all cases the excitation and emission slits were set to 5 nm or 10 nm for the urea –based receptors and the thiourea-based receptors respectively. The artefacts present are presumably due to the compounds aggregating in aqueous solution.

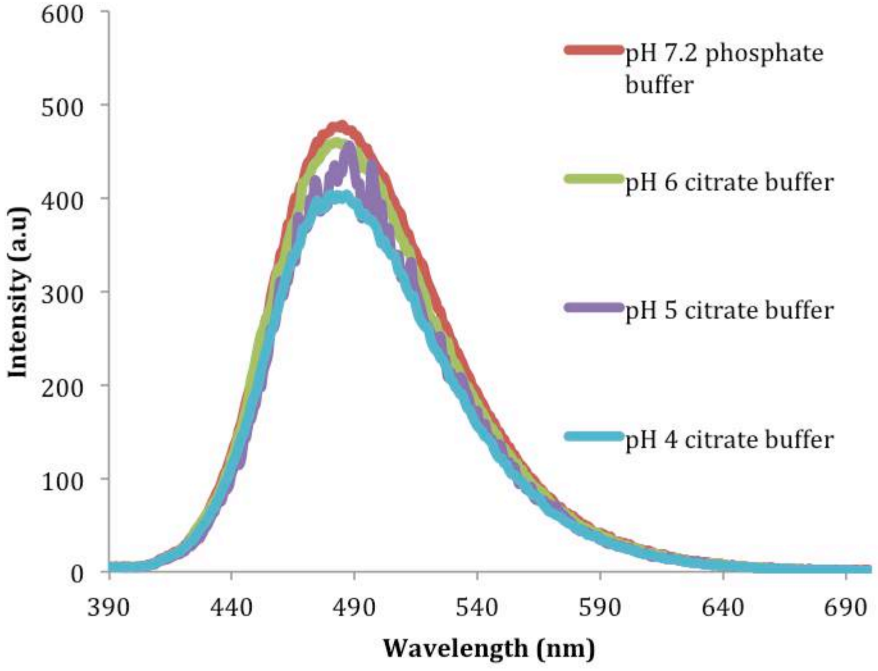

Figure S75: Emission spectra of **1** at various pHs.

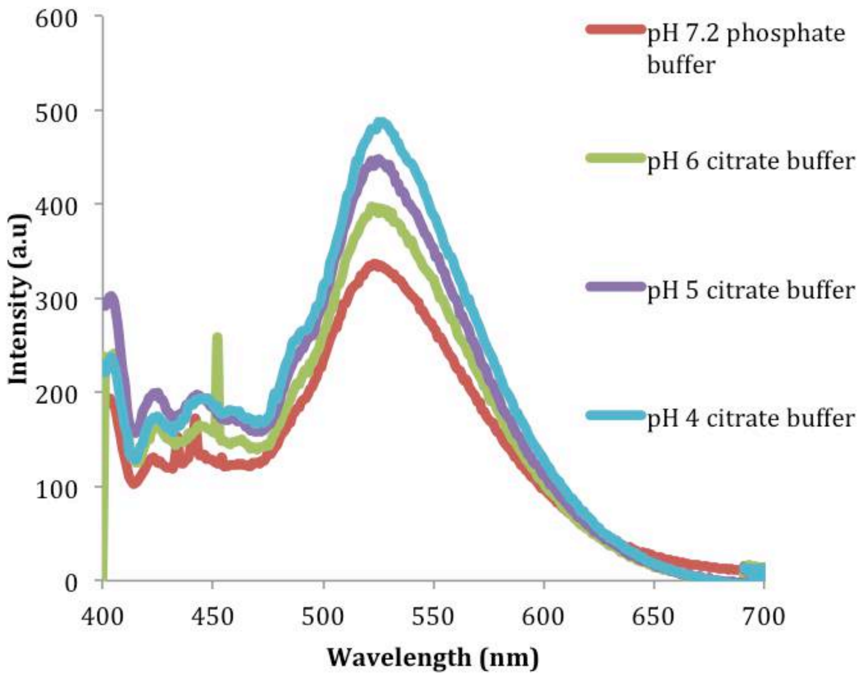

Figure S76: Emission spectra of **2** at various pHs.

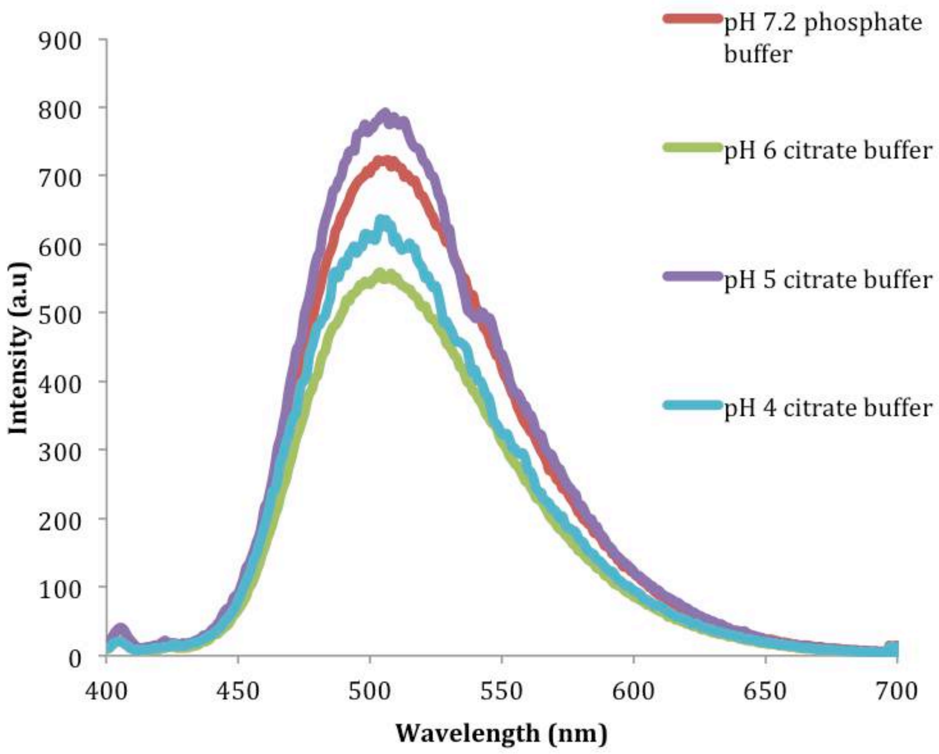

Figure S77: Emission spectra of **3** at various pHs.

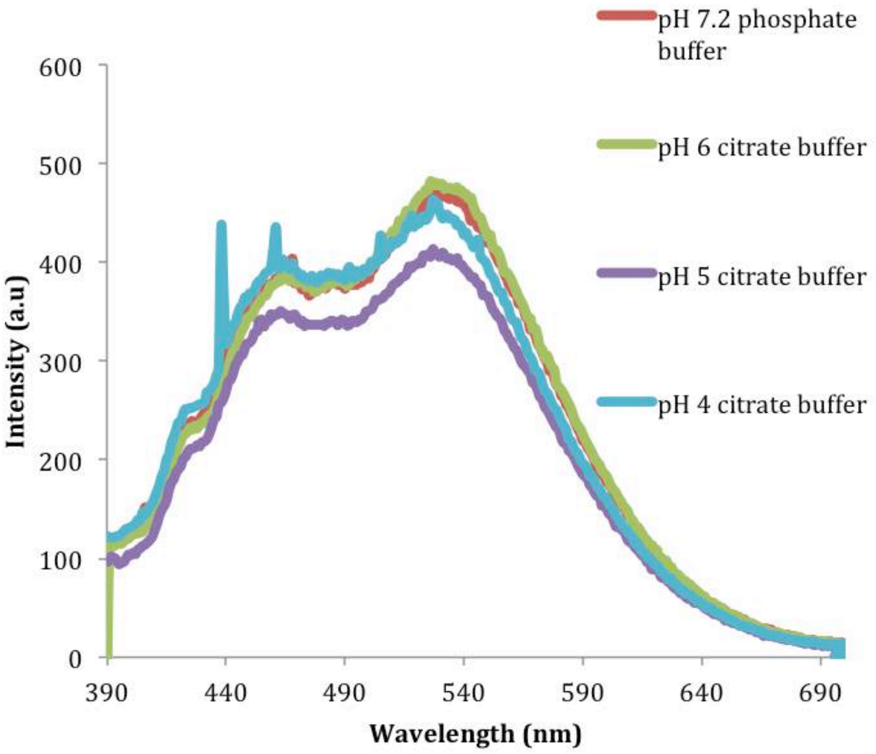

Figure S78: Emission spectra of **4** at various pHs.

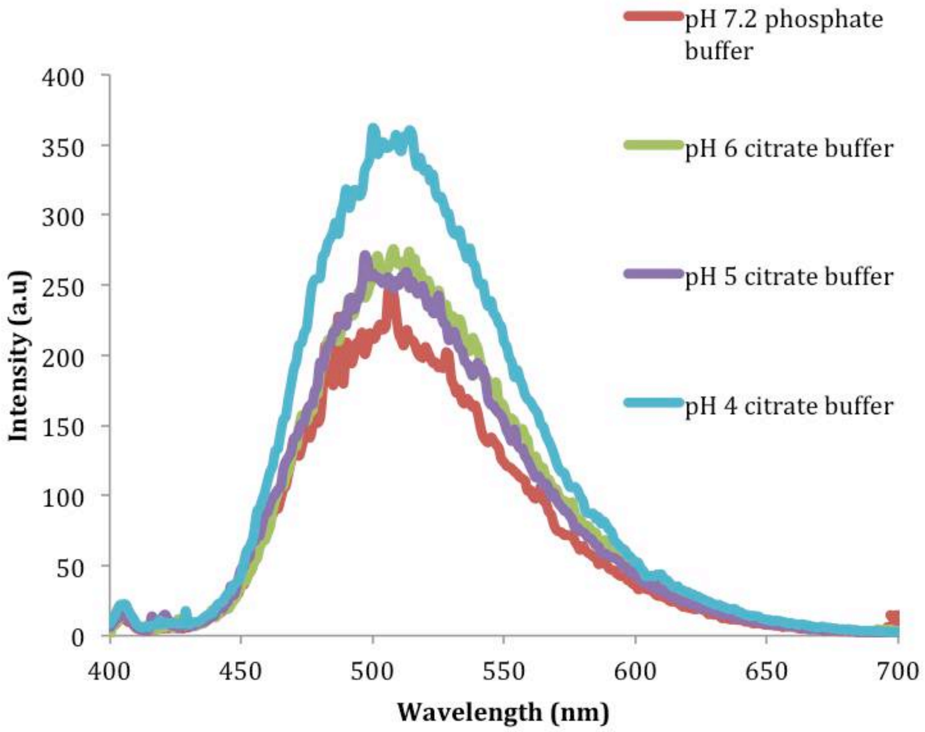

Figure S79: Emission spectra of **5** at various acidic pH.

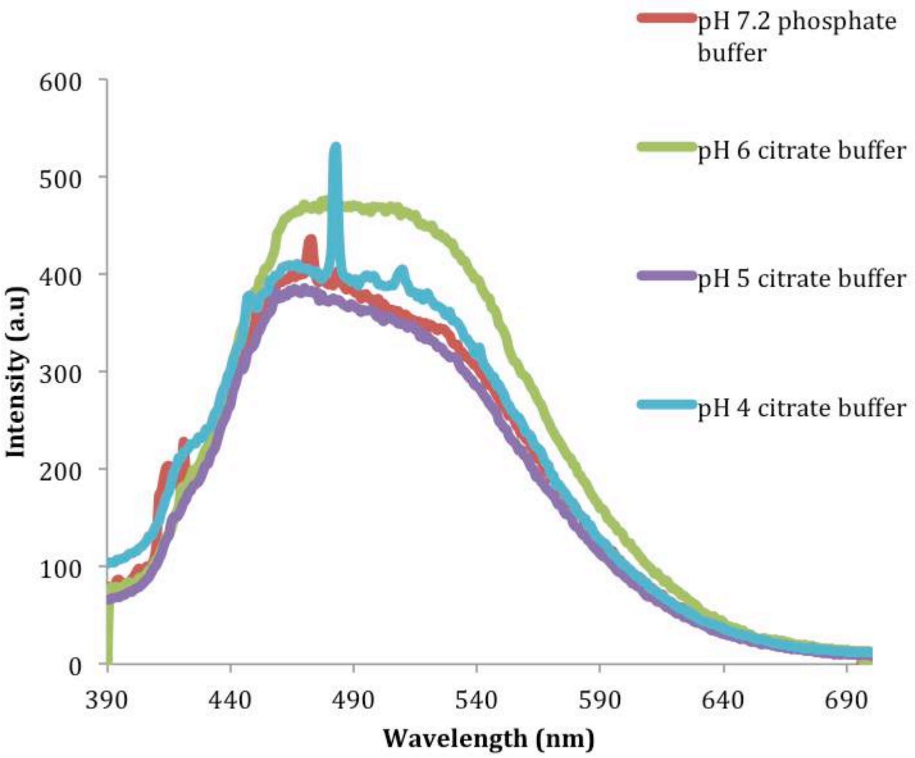

Figure S80: Emission spectra of **6** at various pHs.

6.2 Fluorescence spectra in POPC Vesicles

We examined the fluorescence spectra of **1**-**6** (10 μM) in POPC vesicles (0.5 mM) prepared as previously described suspended in aqueous sodium chloride solution (489 mM) buffered to pH 7.2 with 5 mM sodium phosphate salts. Urea based compounds **1**, **3** and **5** show little signs of aggregation whereas the thiourea-based compounds **2**, **4** and **6** appear to show evidence of aggregation.

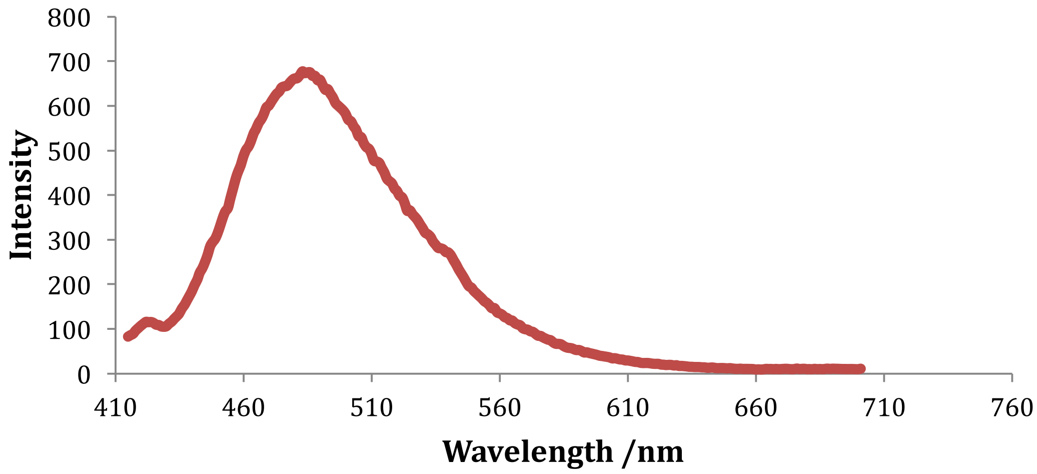

Figure S81: Emission spectra of **1** in POPC vesicles.

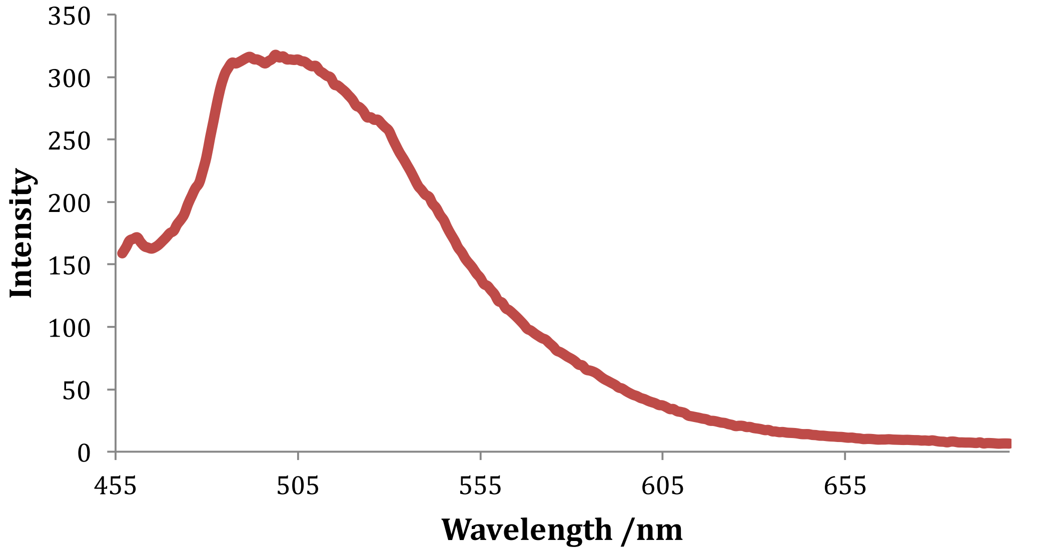

Figure S82: Emission spectra of **2** in POPC vesicles.

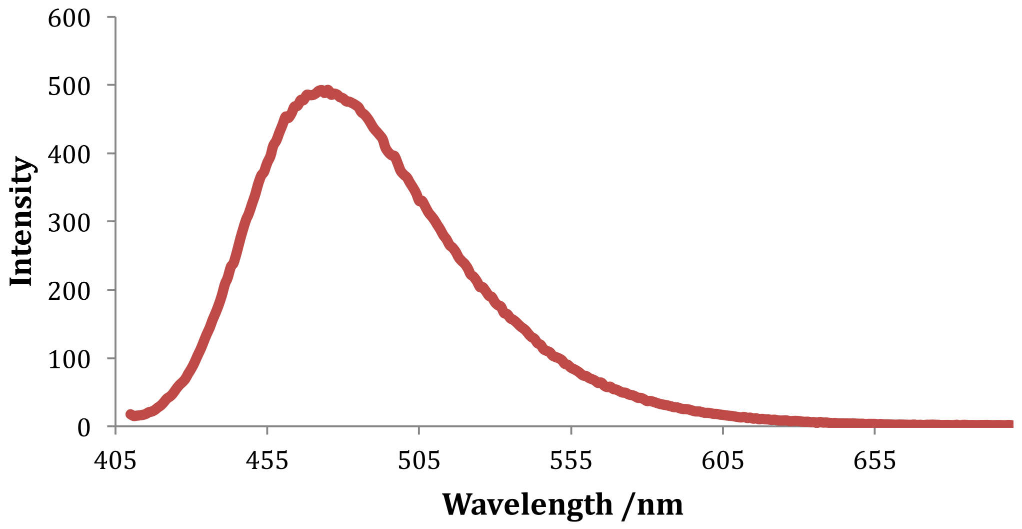

Figure S83: Emission spectra of **3** in POPC vesicles.

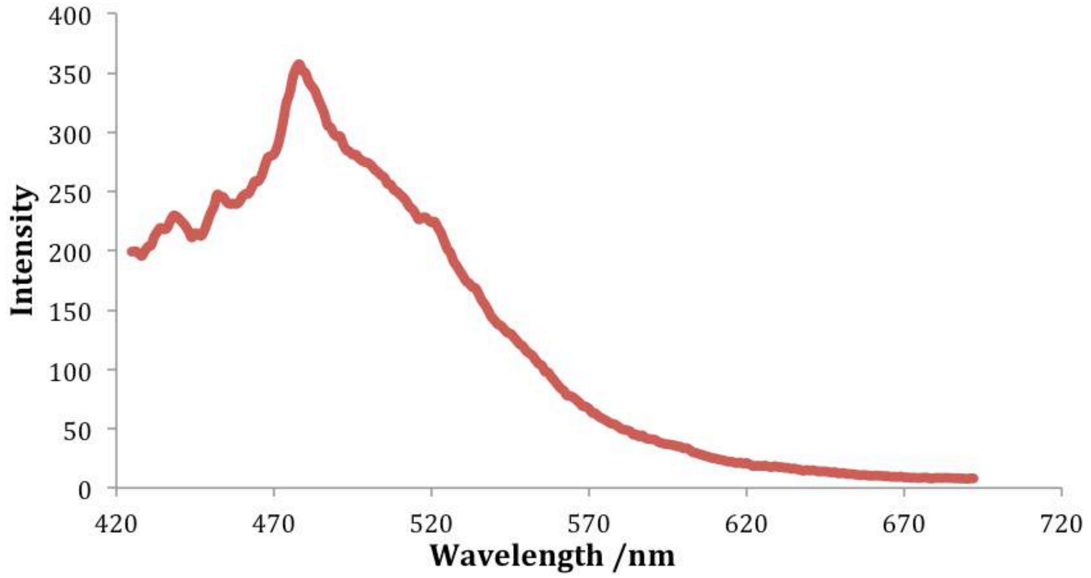

Figure S84: Emission spectra of **4** in POPC vesicles.

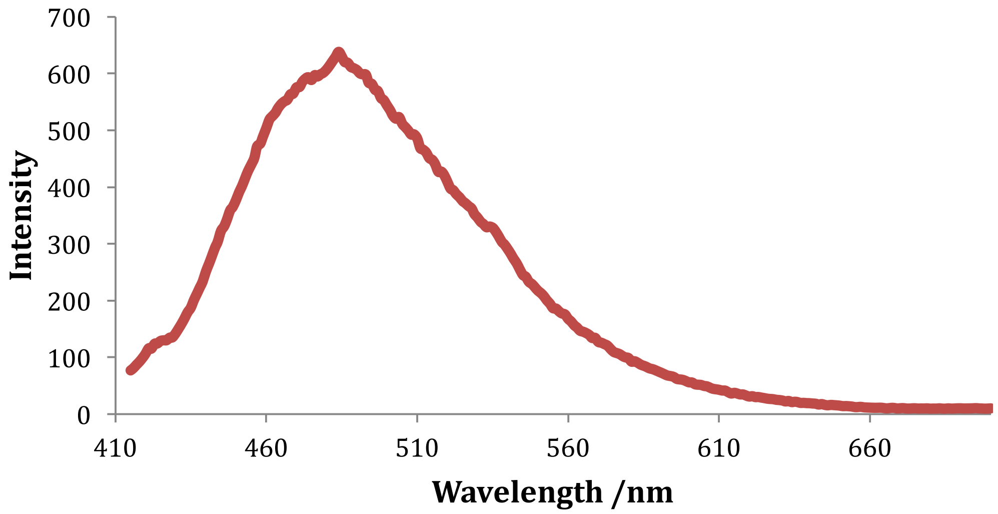

Figure S85: Emission spectra of **5** in POPC vesicles.

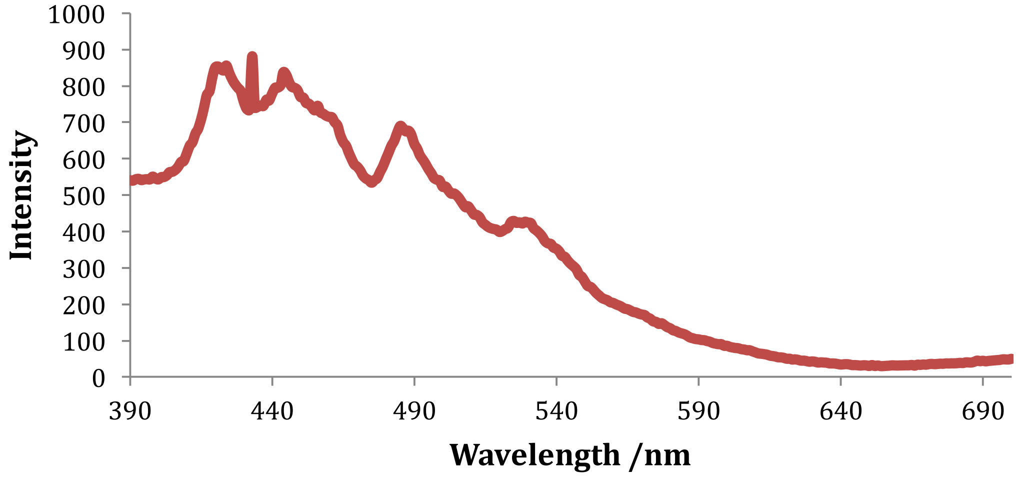

Figure S86: Emission spectra of **6** in POPC vesicles.

7 – Transport Studies

7.1 Overview and Procedure

Unilamellar POPC vesicles were prepared using isotonic intra-vesicular (internal) and extra-vesicular (external) solutions to prevent vesicles from lysing as follows: POPC was dissolved in chloroform (~29 mg/mL). A thin film of lipid was produced by transferring a known quantity of POPC solution to a round-bottomed flask and then removing the solvent *in vacuo*. The film was then dried on a high vacuum line for a minimum of 4 hrs to remove any remaining solvent. The lipid was then suspended in the same volume of internal solution as the initial chloroform solution used using a lab dancer. Next, 9 freeze-thaw cycles were completed by alternating the lipid between liquid nitrogen and room temperature water. The lipid was then left to stand for 30 minutes. The formed vesicles were then extruded through a 200 nm polycarbonate membrans 25 times before being placed in dialysis tubing and then into the desired external solution a minimum of 2 hours. This removes any unencapsulated internal salts. Finally, the lipid was diluted to 1mM using the required external solution.

7.2 Cl^-/NO_3^- Transport Studies

Cl^-/NO_3^- antiport tests were conducted as follows: unilamellar POPC vesicles were prepared containing aqueous internal solution with 489 mM NaCl buffered to pH 7.2 with 5 mM sodium phosphate salts. The vesicles were dispersed in an aqueous external solution containing 489 mM $NaNO_3$ buffered to pH 7.2 with 5 mM sodium phosphate salts. The receptor was loaded at an appropriate molar percentage relative to the moles of lipid as a DMSO solution at 0 s. After 300 s, Triton X-100 detergent was added to lyse the vesicles (2.32 mM in 7:1 H_2O:DMSO v/v) and calibrate the chloride selective electrode to 100% chloride efflux at 420 s. All endpoint values are taken as of 270 s. Chloride concentrations were monitored using an Accumet solid-state combination chloride selective electrode. The lipid concentration per sample was 1 mM. Each data point represents the average of 3 runs. For the blank runs (pure DMSO) there is a minimum of one repeat and a maximum of three. Error bars are shown where appropriate.

Additional Cl^{-}/NO_3^{-} Transport Graphs:

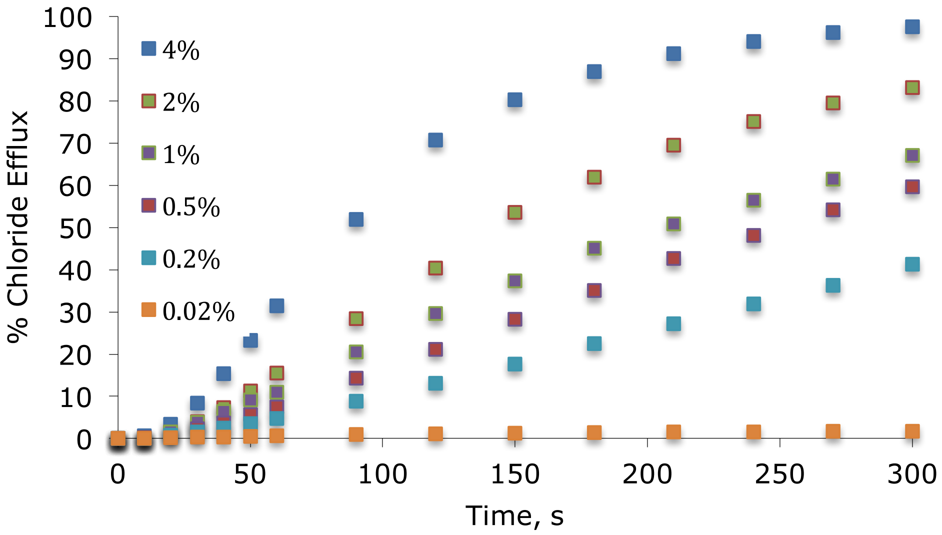

Figure S87: Chloride efflux as a function of time, promoted by the addition of various concentrations of receptor **2**, from unilamellar POPC vesicles containing 489 mM NaCl with 5 mM sodium phosphate salts. The vesicles were suspended in 489 mM $NaNO_3$ buffered to pH 7.2 with 5 mM sodium phosphate salts. The receptor was loaded as a DMSO solution at 0 s. The vesicles were lysed at the end of the experiment to calibrate 100% chloride efflux.

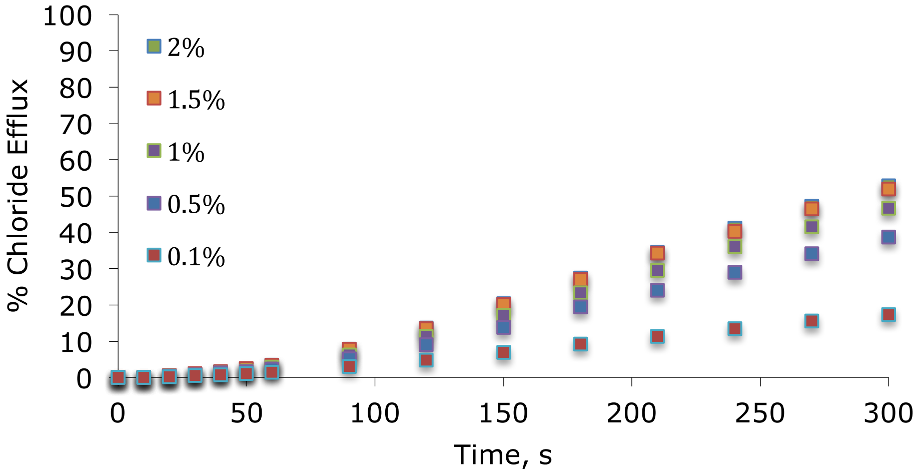

Figure S88: Chloride efflux as a function of time, promoted by the addition of various concentrations of receptor **3**, from unilamellar POPC vesicles containing 489 mM NaCl with 5 mM sodium phosphate salts. The vesicles were suspended in 489 mM $NaNO_3$ buffered to pH 7.2 with 5 mM sodium phosphate salts. The receptor was loaded as a DMSO solution at 0 s. The vesicles were lysed at the end of the experiment to calibrate 100% chloride efflux.

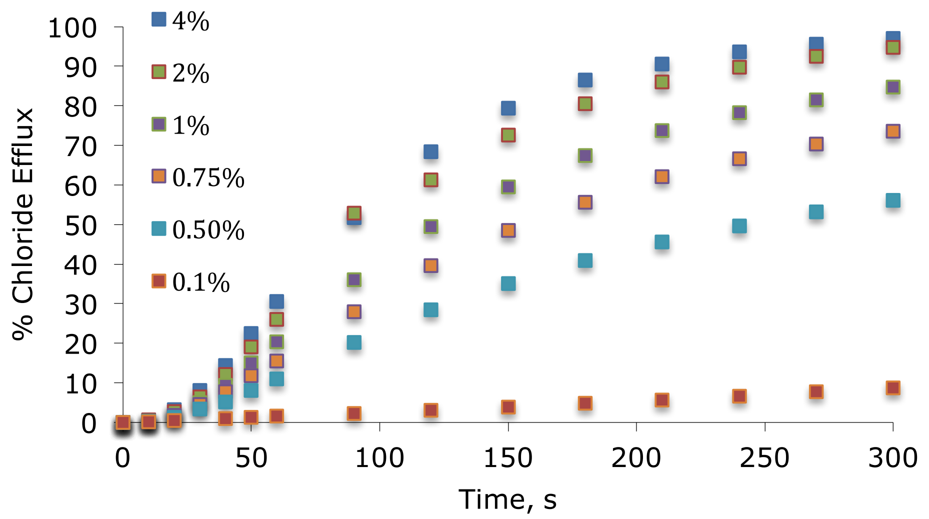

Figure S89: Chloride efflux as a function of time, promoted by the addition of various concentrations of receptor **4**, from unilamellar POPC vesicles containing 489 mM NaCl with 5 mM sodium phosphate salts. The vesicles were suspended in 489 mM $NaNO_3$ buffered to pH 7.2 with 5 mM sodium phosphate salts. The receptor was loaded as a DMSO solution at 0 s. The vesicles were lysed at the end of the experiment to calibrate 100% chloride efflux.

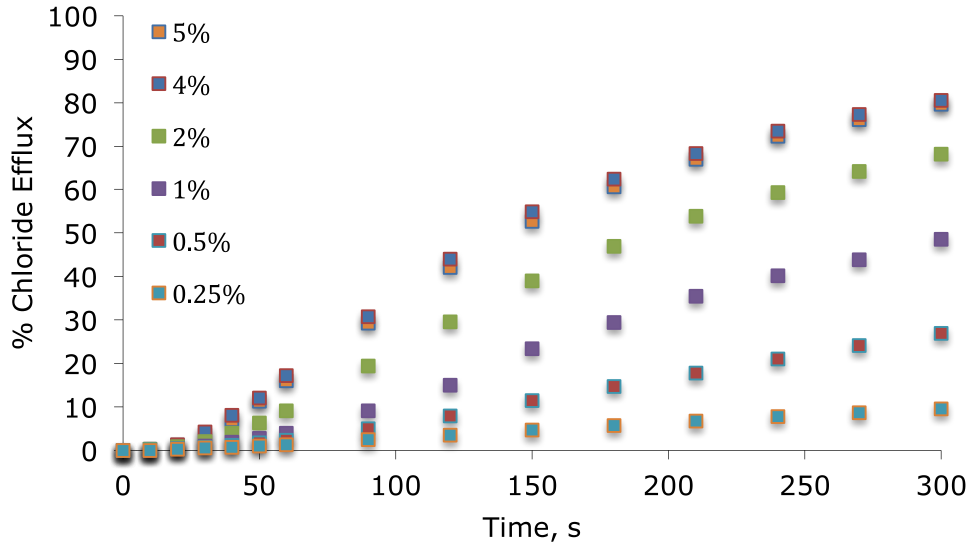

Figure S90: Chloride efflux as a function of time, promoted by the addition of various concentrations of receptor **6**, from unilamellar POPC vesicles containing 489 mM NaCl with 5 mM sodium phosphate salts. The vesicles were suspended in 489 mM $NaNO_3$ buffered to pH 7.2 with 5 mM sodium phosphate salts. The receptor was loaded as a DMSO solution at 0 s. The vesicles were lysed at the end of the experiment to calibrate 100% chloride efflux.

7.3 Hill Plots and Analysis

Hill plots were conducted for Cl^-/NO_3^- transport assays by performing transport assays at various concentrations of receptor. Plots of receptor concentration vs chloride efflux at 270 s (the endpoint of transport assay) were fitted to the Hill equation using Origin 9.1:

$$y = V_{max} \frac{x^n}{k^n + x^n} = 100\% \frac{x^n}{(EC_{50})^n + x^n}$$

This is where y is the chloride efflux at 270 s (%) and x is the receptor concentration (mol % with respect to lipid concentration). V_{max}, k and n are the parameters to be determined. V_{max} is the maximum efflux possible and is therefore fitted to 100% as this is physically the maximum chloride efflux possible. k is the carrier concentration needed to reach $V_{max}/2$ and therefore when V_{max} is fixed to 100%, k is the EC_{50} value. Each data point on each Hill plot are an average of three repeated runs. Error bars represent standard deviation about the mean.

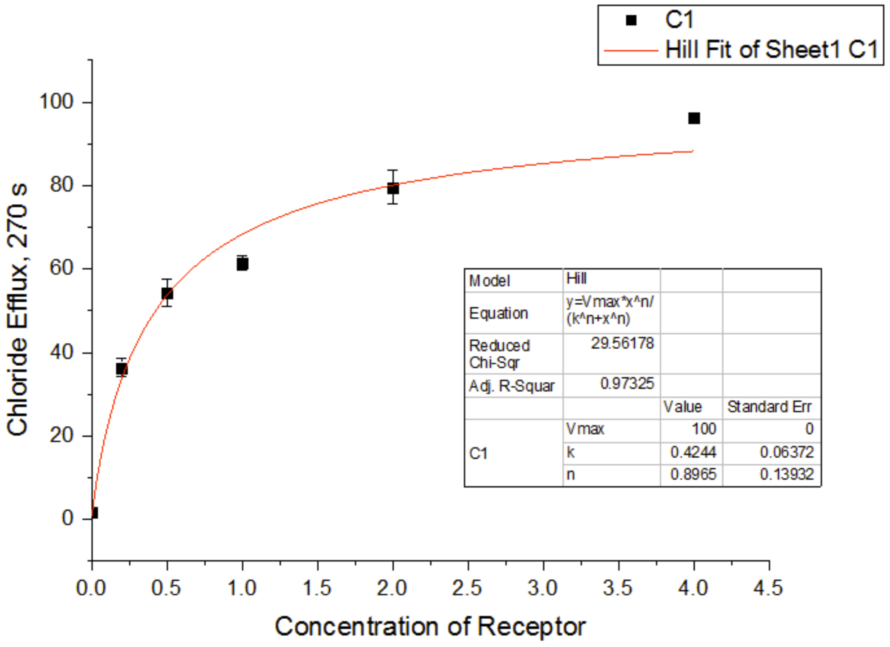

Figure S91: Hill analysis for Cl^-/NO_3^- antiport mediated by **2**. Data fitted to the Hill equation using Origin 9.1.

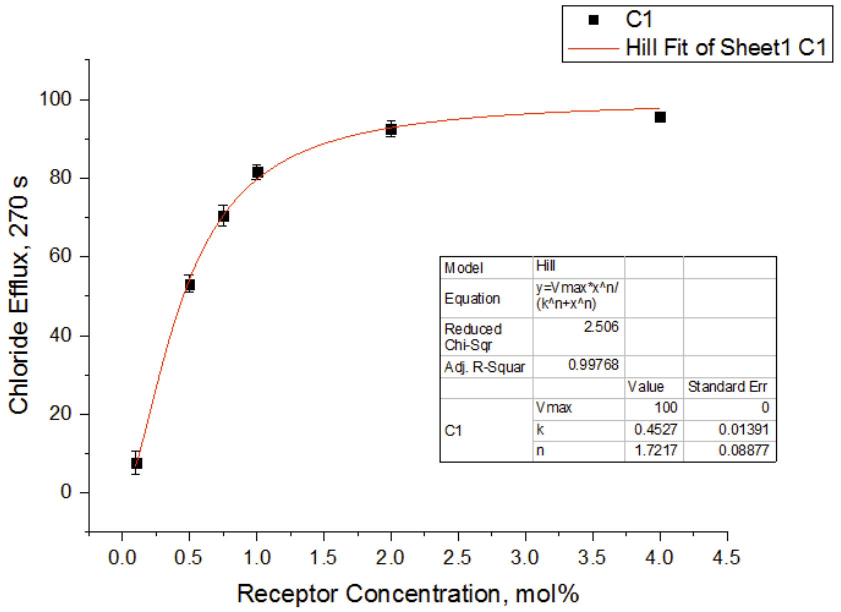

Figure S92: Hill analysis for Cl^-/NO_3^- antiport mediated by **4**. Data fitted to the Hill equation using Origin 9.1.

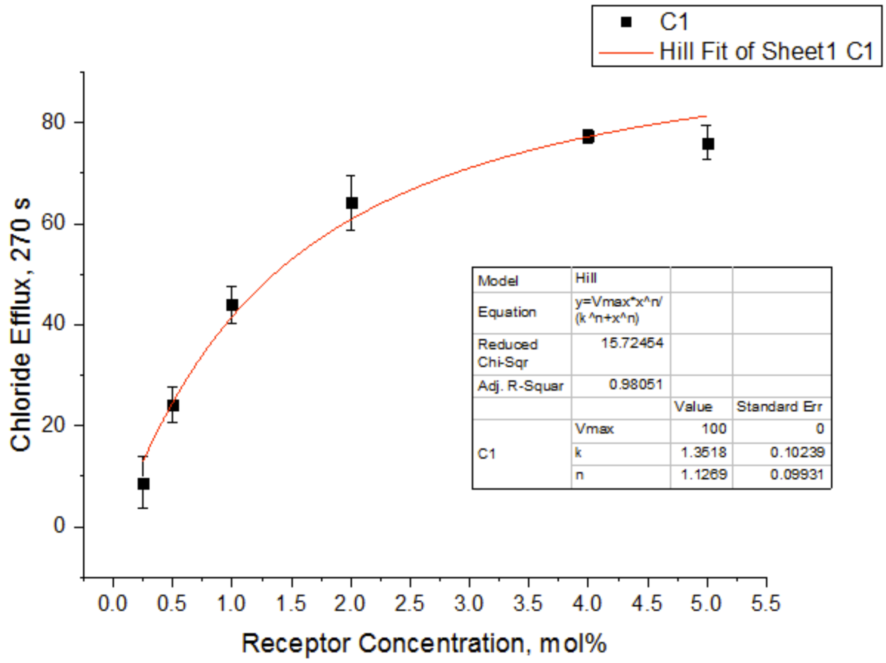

Figure S93: Hill analysis for Cl^-/NO_3^- antiport mediated by **6**. Data fitted to the Hill equation using Origin 9.1.

7.4 Cl^-/HCO_3^- Transport Studies

Cl^-/HCO_3^- transport assays were carried out in a similar way to the Cl^-/NO_3^- assays. Unilamellar POPC vesicles containing 489 mM NaCl solution buffered to pH 7.2 with 20 mM sodium phosphate salts, prepared as described above, were suspended in the external medium consisting of a 162 mM Na_2SO_4 solution buffered to pH 7.2 with sodium phosphate salts (20 mM buffer). The lipid concentration per sample was 1 mM. A DMSO solution of the carrier molecule (10 mM) was added to start the experiment and chloride efflux was monitored using a chloride sensitive electrode. At 2 min, a $NaHCO_3$ solution (1 M in 162 mM Na_2SO_4 buffered to pH 7.2 with 20 mM sodium phosphate salts) was added so that the outer solution contained 40 mM $NaHCO_3$. At 7 min, the vesicles were lysed with 50 µl of triton X-100 (2.32 mM in 7:1 water:DMSO v/v) and a total chloride reading was taken at 9 min.

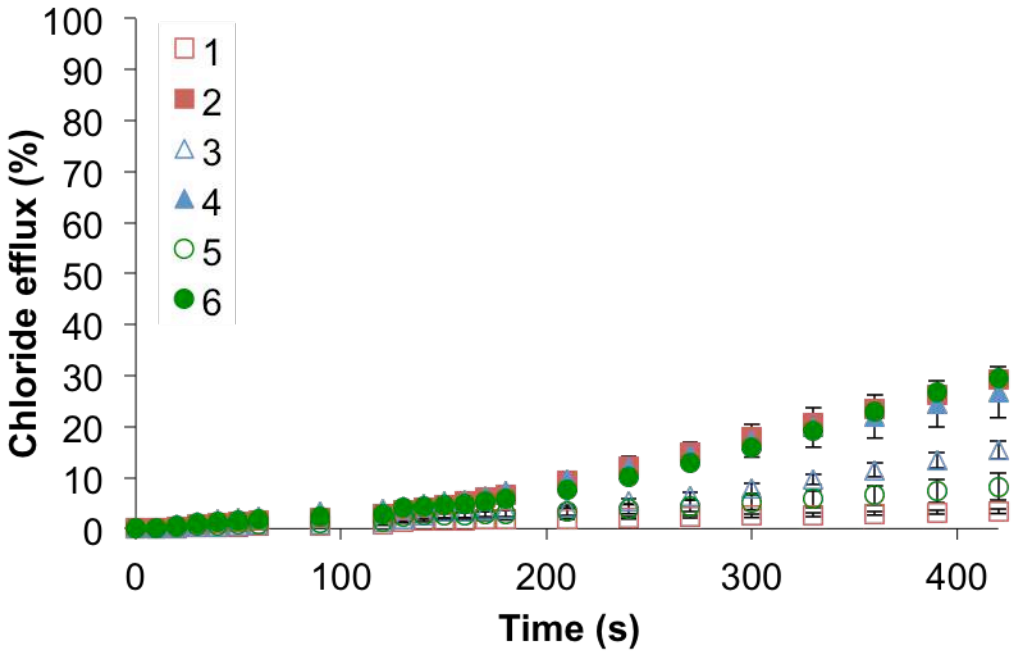

Figure S94: Chloride efflux promoted by a DMSO solution of compounds **1**-**6** (2 mol% receptor concentration versus lipid) from unilamellar POPC vesicles loaded with 489 mM NaCl buffered to pH 7.2 with 20 mM sodium phosphate salts. At t = 120 s a solution of sodium bicarbonate was added such that the external bicarbonate concentration was 40 mM. At the end of the experiment, the vesicles were lysed to calibrate the electrode to 100% efflux. Each point is an average of 3 repeated runs, error bars are ± standard deviation about the mean.

7.5 Evidence for Antiport

To test for possible Na^+/Cl^- symport, we repeated the Cl^-/NO_3^- transport assay but with vesicles encapsulating CsCl instead of NaCl. The comparison graph below shows neglible difference (within error) or transport between NaCl containing vesicles and CsCl vesicles, effectively ruling out metal/choride symport as a transport mechanism.

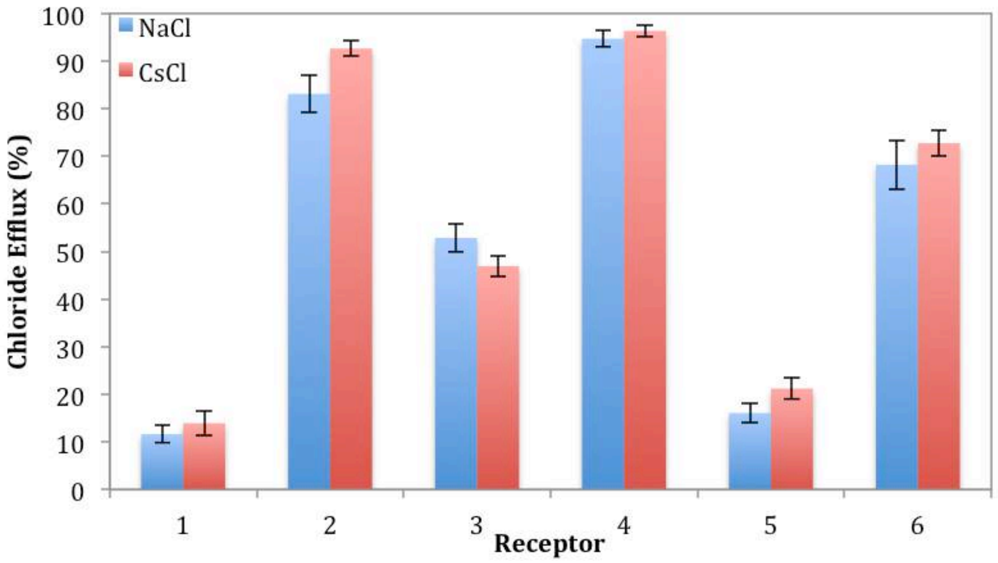

Figure S95: Comparison graph showing chloride efflux at 270 s promoted by receptors **1**-**6** from unilamellar POPC vesicles (200 nm) containing NaCl (489 mM buffered to pH 7.2with 5 mM sodium phosphate salts, blue bars) and CsCl (489 mM buffered to pH 7.2 with 5 mM sodium phosphate salts, red bars). Both sets of vesicles were suspended an external solution of $NaNO_3$ (498 mM, buffered to pH 7.2 with 5 mM sodium phosphate salts). Receptors were added at 2 mol% with respect to POPC concentration. Graph shows negligible discrepancies between NaCl and CsCl containing vesicles.

8 – In Vivo Assays

General cell culture information:

Human cell lines A549 (lung carcinoma) and MCF-7 (breast adenocarcinoma) were obtained from the American Type Culture Collection (ATCC, Manassas VA) and maintained in DMEM media (Biological Industries, Beit Haemek, Israel) supplemented with 100 U/mL penicillin, 100 µg/mL streptomycin, and 2 mM L-glutamine, all from Biological Industries and 10% fetal bovine serum (FBS; Invitrogen-Life Technologies, Carlsbad, CA). Cells were grown at 37 °C under a 5% CO_2 atmosphere.

8.1 Cell viability assay (MTT)

Cell viability was determined by the MTT assay. Cells (1×10^5 cells/mL) were seeded in 96-well microtiter plates and incubated for 24 h to allow cells to attach. Afterwards, they were treated for 24 h with different compound concentrations to obtain the dose-response curves (range 0.78 to 100 µM) and inhibitory concentration of 50% of cell population (IC_{50} values) were calculated. Compound diluent (maximum 1% DMSO) was added to control cells. Then, 10 µM of 3-(4,5-dimethylthiazol-2-yl)-2,5-diphenyltetrazolium bromide diluted in PBS (MTT, Sigma-Aldrich, St Louis, MO) was added to each well for an additional 2 h. The medium was removed and the MTT formazan precipitate was dissolved in 100 µl of DMSO. Absorbance was read on a Multiskan multiwell plate reader (Thermo Scientific Inc., Waltham, MA) at 570 nm.

For each condition, at least three independent experiments were performed in duplicate. Cell viability was expressed as a percentage of control cells, and data are shown as the mean value ± S.D. Dose-response curves and IC_{50} values were calculated with GraphPad Prism 5 software. Compounds **1** and **2** showed no toxicity at 50 µM.

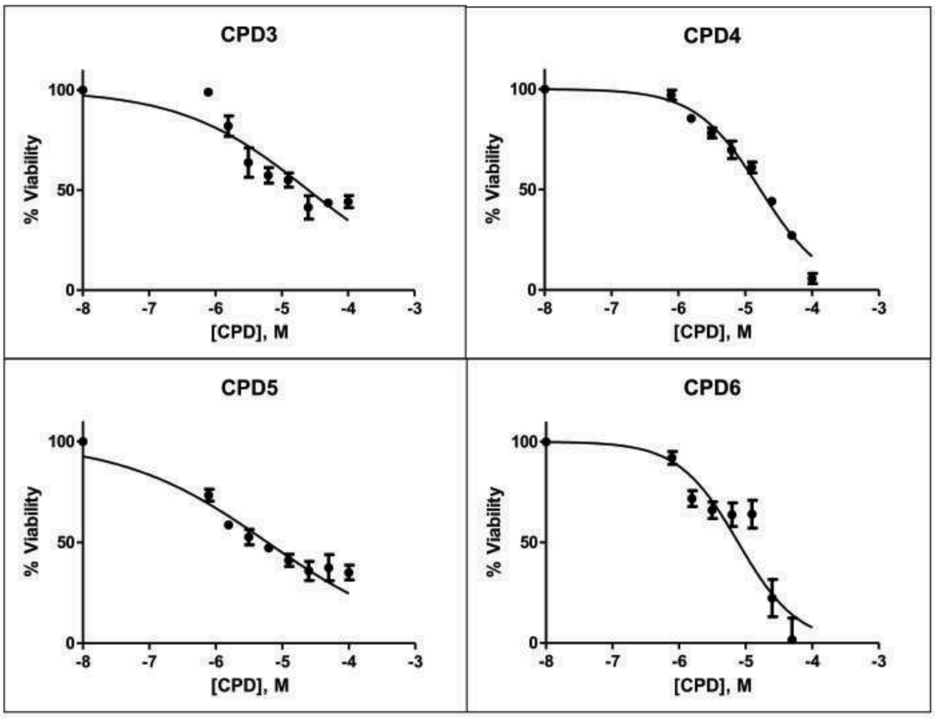

Fig S96: Dose-response cell viability curves from A549 cells treated with varying doses of **3**-**6** measured by MTT assay.

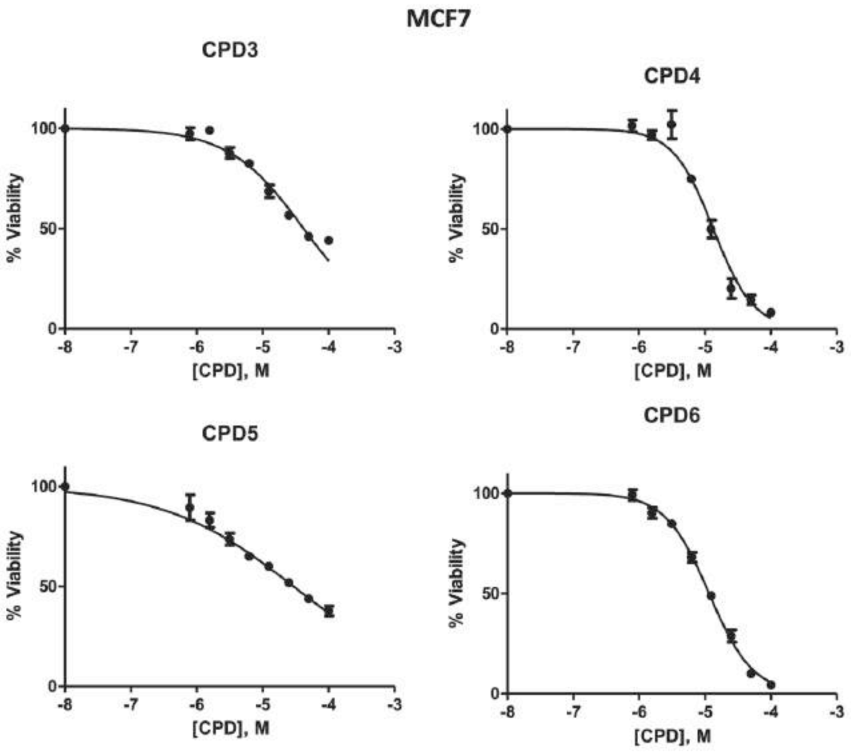

Fig S97: Dose-response cell viability curves from MCF-7 cells treated with varying doses of **3**-**6** measured by MTT assay.

8.2 Annexin-V Assay

A549 cells ($2x10^5$ cells) were seeded in 6-well plates and, after 24 h, they were treated with 50, 75 and 100 µM of compound **6** for an additional 24 h. Afterwards, cells were detached, centrifuged at 300g for 5 min, washed with 1X PBS and centrifuged again. Finally, cells were resuspended in 200 µl of 1X PBS-1%FBS:Annexin-V kit buffer (1:1; Muse Annexin V & Dead Cell Assay, Merck Millipore, Merck KGaA, Darmstadt, Germany). After 20 min incubation at room temperature, cells were examined on the Muse™ Cell Analyzer (Merck Millipore). All the conditions were assessed at least in three independent experiments. Results are shown as the mean value ± S.D. and representative results are shown in the selected histograms.

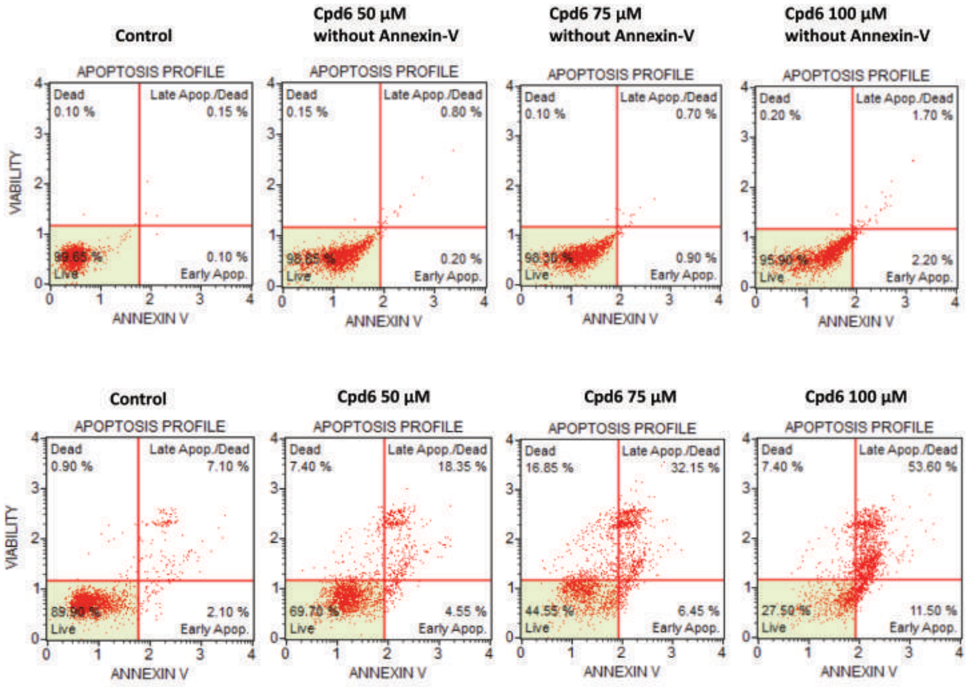

Fig S98: Flow cytometry of A549 cells treated with various concentrations of **6** for 24 hours and stained with Annexin-V and PI (annexin V binding versus PI uptake, 'viability'). Top row shows **6** at various concentrations without Annexin-V, bottom row shown **6** plus Annexin-V.

Discussion on Annexin Results:

Compound **6** exhibited significant autofluorescence in this Annexin assay (fig. S98 top row), however, by increasing the threshold for the 'live cells', we were still able to clearly detect late apoptosis at high receptor loadings. Due to the high threshold set, early apoptosis was not detectable through this assay. Moreover, the late apoptosis occurred at concentrations higher than the receptors IC_{50} value, suggesting the possibility that cell cycle arrest and/or another type of cell death was taking place at low concentrations. It is probable therefore, that **6** causes cell death by a mixture of pathways, by apoptosis and another type, probably necrosis, as suggested by visible changes in morphology and debris observed when investigating these compounds in cells at high loadings.

8.3 Fluorescence Imaging of Transporters in Cells

A549 cells were seeded in 6 well plates ($1x10^5$ cells per ml) and allowed to grow continuously for 24 hours before treatment with compounds **1**-**6** for the stated time. Cells were washed twice with PBS prior to incubation with **1**-**6** (1 or 5 µM in 0.5% DMSO/DMEM). Control cells were treated with 0.5% DMSO/DMEM solution. Post treatment, cells were washed twice with PBS and fresh media added to prevent further localisation. The fluorescence was examined with a Nikon ECLIPSE Ti fluorescence microscope at 40 x using the following filter: λ_{ex} = 340-380 nm, λ_{em} = 490-530 nm.

8.3.1 Time dependent cellular localisation studies

In order to determine how quickly **1**-**6** were able to localise in the cells, time-dependent localisation studies were performed where cells were incubated with compound for various incubation time points before washing twice with PBS and images taken. All compounds were detectable in cells after at least 30 minutes incubation time.

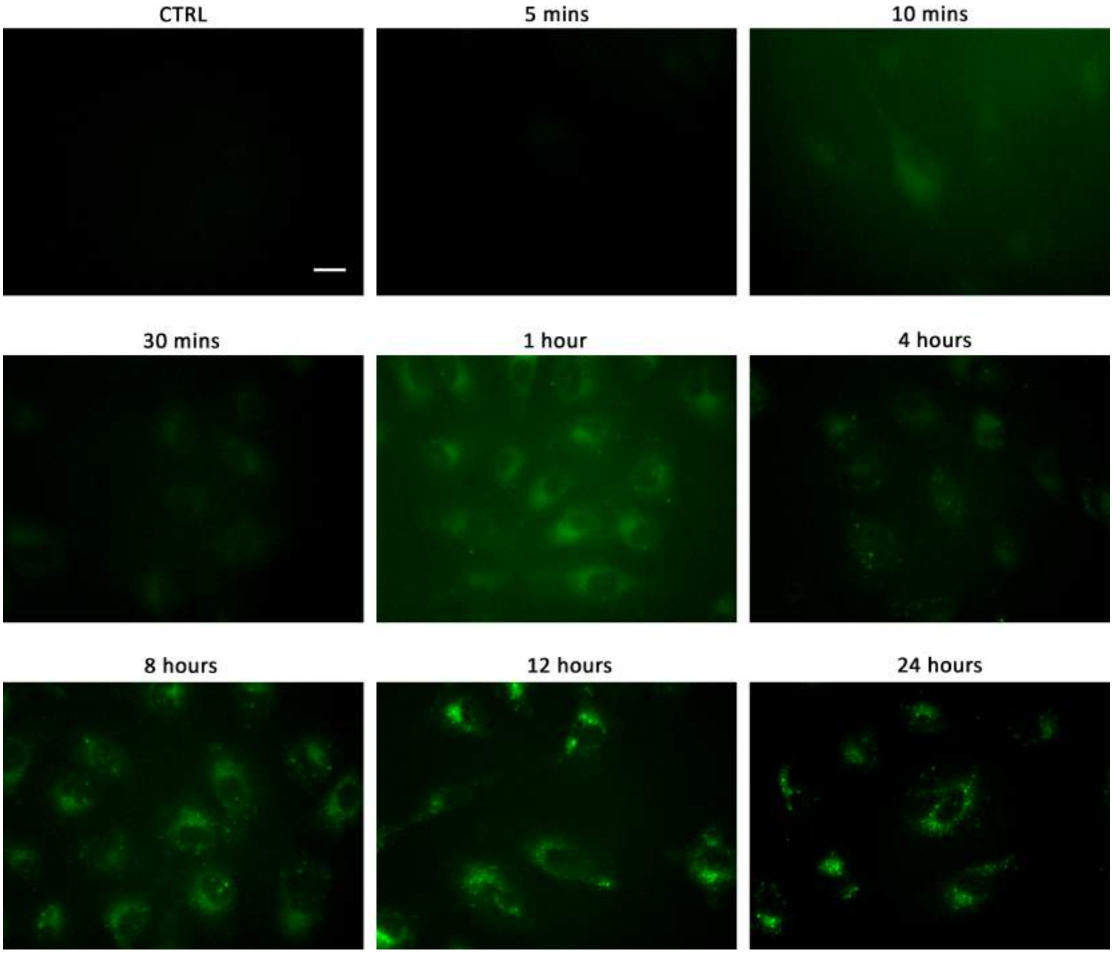

Fig S99: Fluorescence micrographs of A549 cells imaged after various incubation time points with compound **1** (1 µM). Control image is of A549 cells imaged after 24 hours incubation with 0.5% DMSO/DMEM. Scale bar is 20 µm. Images were corrected for contrast to the brightest image (24 hrs) so as to be comparable.

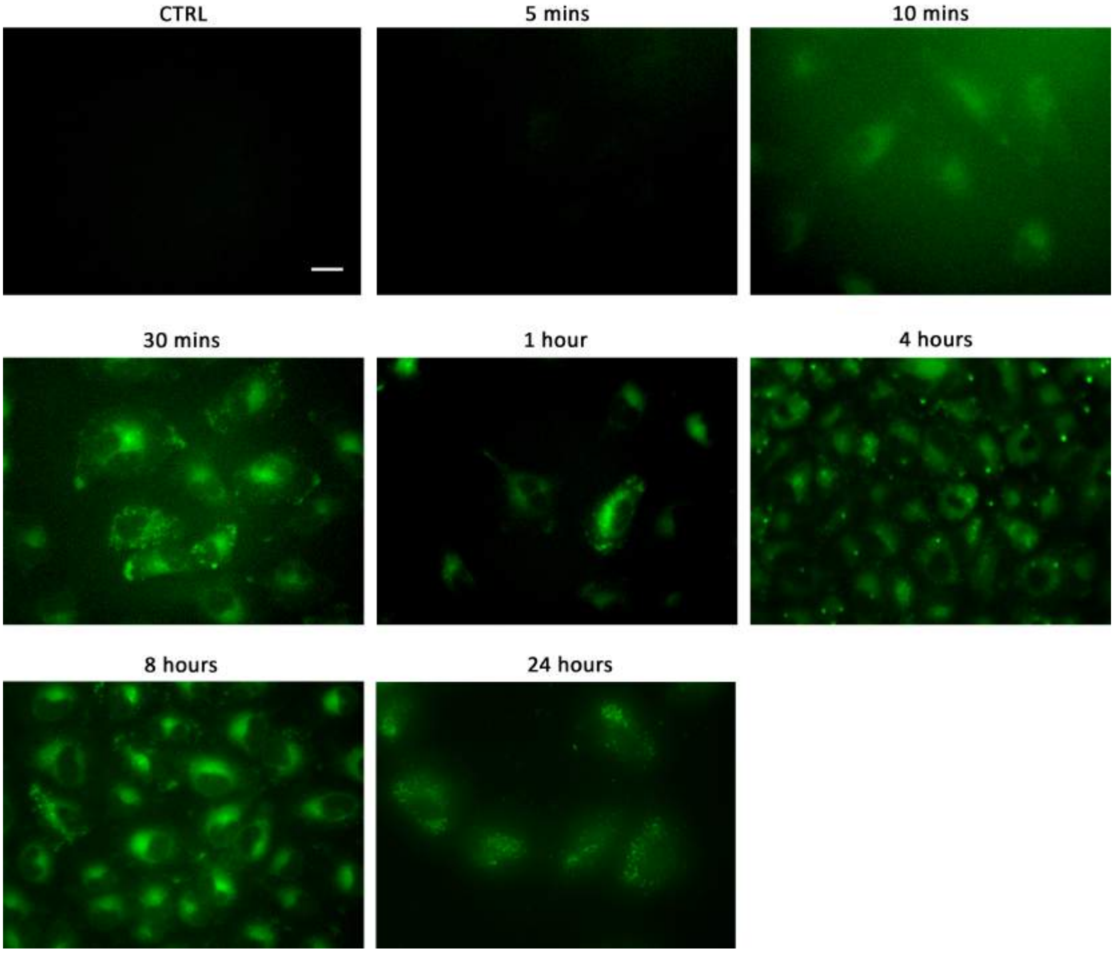

Fig S100: Fluorescence micrographs of A549 cells imaged after various incubation time points with compound **2** (1 µM). Control image is of A549 cells imaged after 24 hours incubation with 0.5% DMSO/DMEM. Scale bar is 20 µm. Images were corrected for contrast to the brightest image (24 hrs) so as to be comparable.

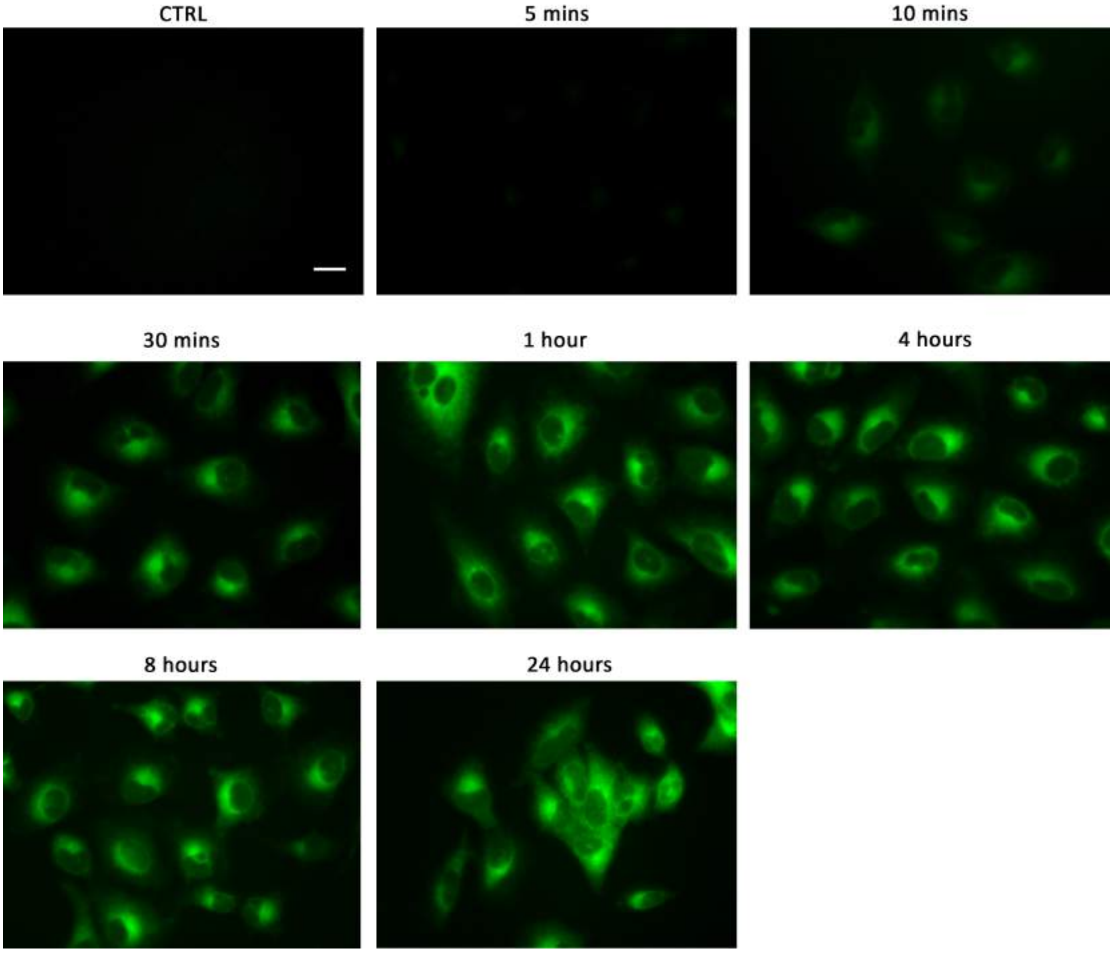

Fig S101: Fluorescence micrographs of A549 cells imaged after various incubation time points with compound **3** (1 µM). Control image is of A549 cells imaged after 24 hours incubation with 0.5% DMSO/DMEM. Scale bar is 20 µm. Images were corrected for contrast to the brightest image (24 hrs) so as to be comparable.

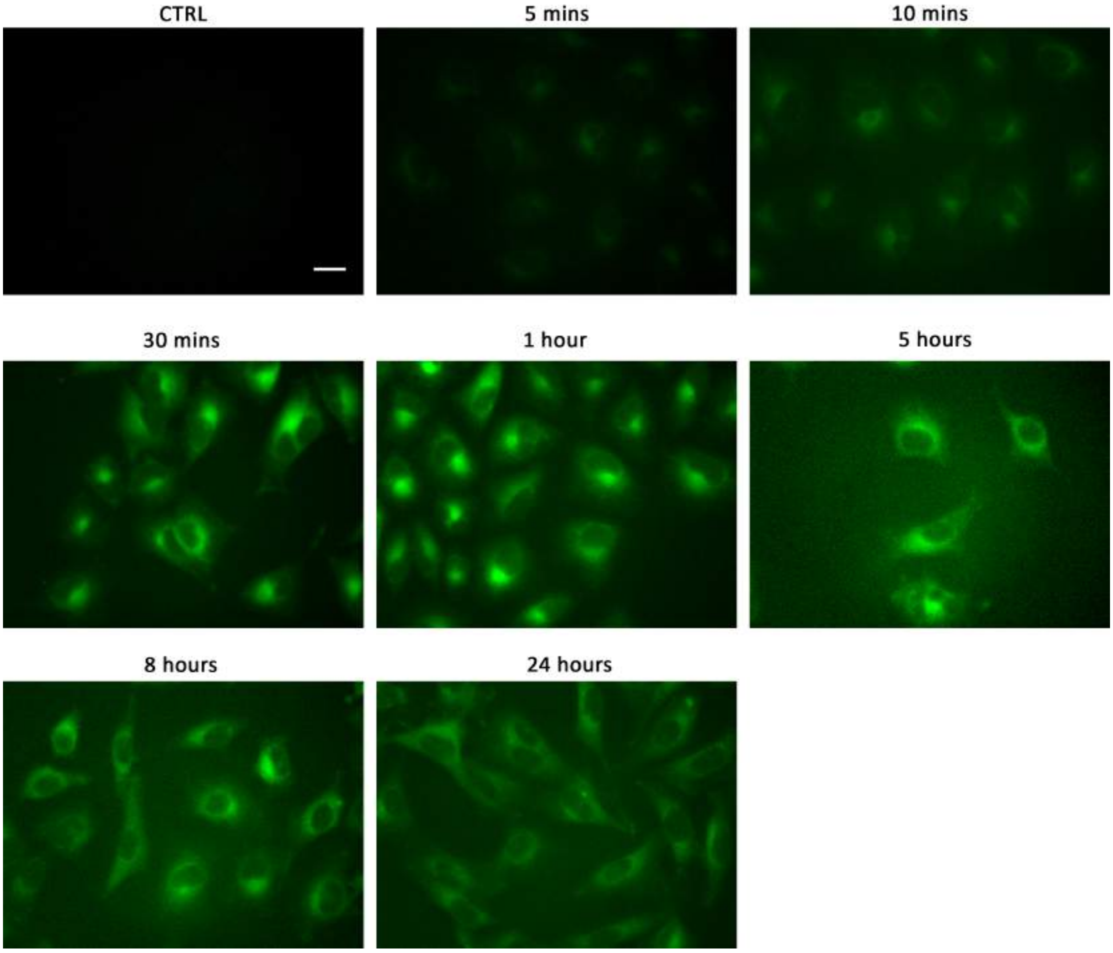

Fig S102: Fluorescence micrographs of A549 cells imaged after various incubation time points with compound **4** (1 µM). Control image is of A549 cells imaged after 24 hours incubation with 0.5% DMSO/DMEM. Scale bar is 20 µm. Images were corrected for contrast to the brightest image (24 hrs) so as to be comparable.

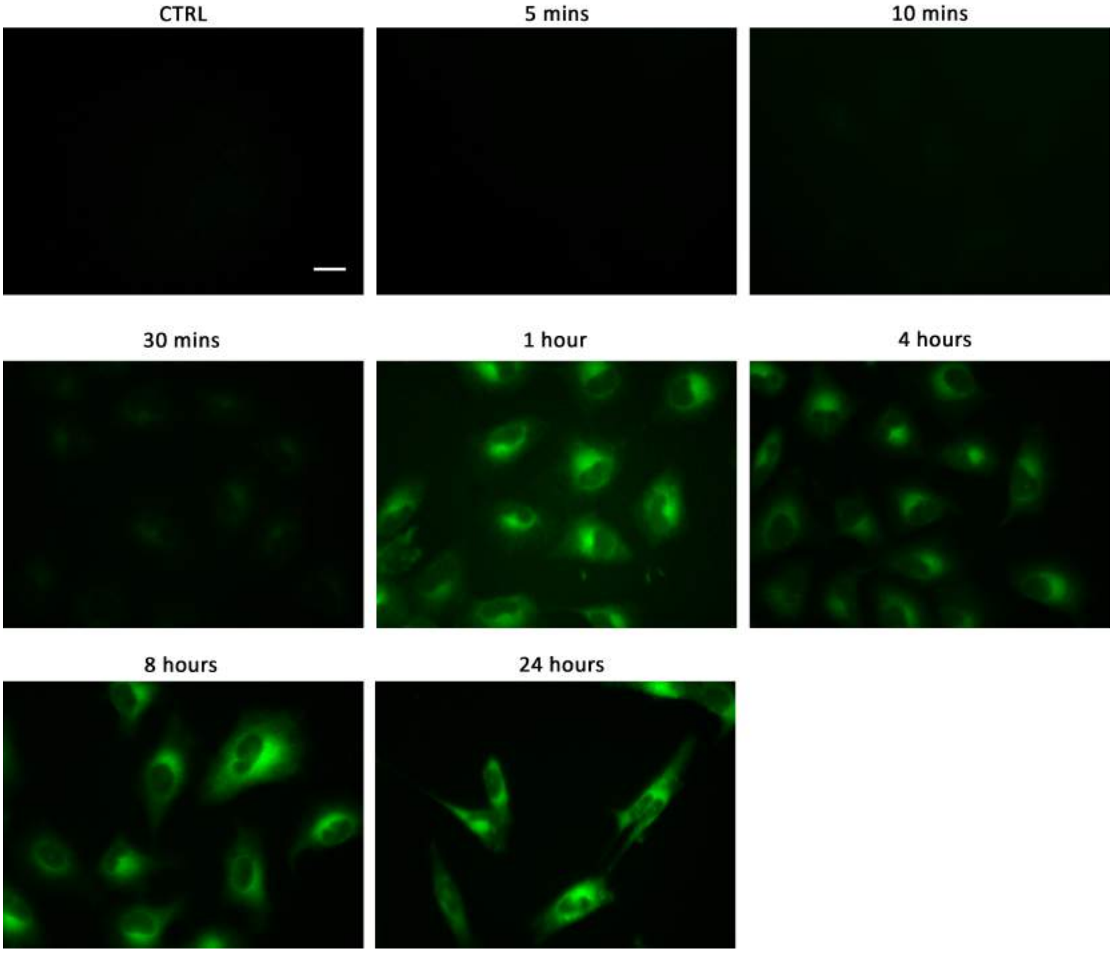

Fig S103: Fluorescence micrographs of A549 cells imaged after various incubation time points with compound **5** (1 µM). Control image is of A549 cells imaged after 24 hours incubation with 0.5% DMSO/DMEM. Scale bar is 20 µm. Images were corrected for contrast to the brightest image (24 hrs) so as to be comparable.

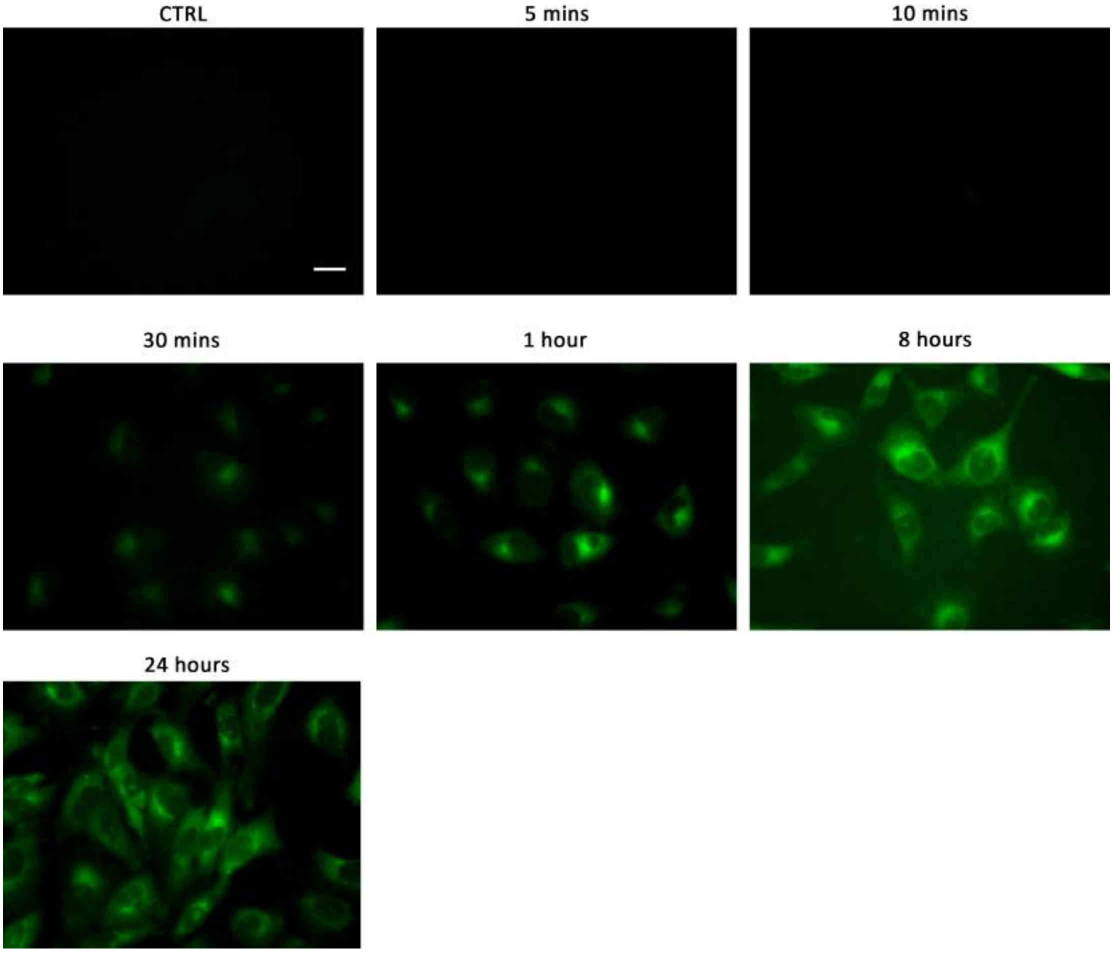

Fig S104: Fluorescence micrographs of A549 cells imaged after various incubation time points with compound **6** (1 μM). Control image is of A549 cells imaged after 24 hours incubation with 0.5% DMSO/DMEM. Scale bar is 20 μm. Images were corrected for contrast to the brightest image (24 hrs) so as to be comparable.

8.3.2 'Washing-Out' Imaging Experiments

'Washing-out' experiments were performed whereby we investigated the reversibility of transporters **1**-**6** to cross the cells plasma membrane. These experiments were performed as follows: A549 cells ($1x10^5$ cells/mL) were seeded in a 6 well plate and allowed to grow continuously for 24 hours before treatment with compounds **1**-**6**. Cells were washed twice with PBS and then incubated in a solution of **1**-**6** (1 or 5 μM in 0.5% DMSO/DMEM) for 1 hour. After this hour, the cells were washed twice with PBS and fluorescence micrographs taken (1st wash). Following this time, the cells were place back in the incubator at 37 °C for 1 hour, washed twice

with PBS and imaged again (2nd wash). The cells were then incubated again for a final hour, washed twice with PBS and imaged a final time (3rd time). All images were corrected for contrast to the brightest image (1st wash) so as to be comparable.

The 'washing out' experiments showed that these compound were easily washed out of the cells, demonstrating reversible crossing over the cells plasma membrane, much in the same way we presume these compounds cross POPC bilayers in the transport studies.

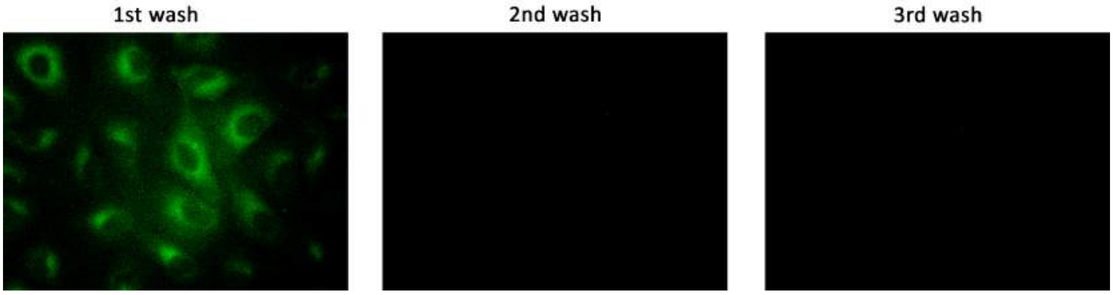

Fig. S 105: Fluorescent micrographs from 'washing out' experiment for compound **1**. A549 cells were incubated with **1** (1 µM) for 1 hour, washed twice with PBS and then imaged. Cells were then returned to the incubator for a second hour, washed twice and then imaged again (2nd wash). This process was repeated a third time (3rd wash). Images were corrected for contrast to the brightest image (1st wash) so as to be comparable.

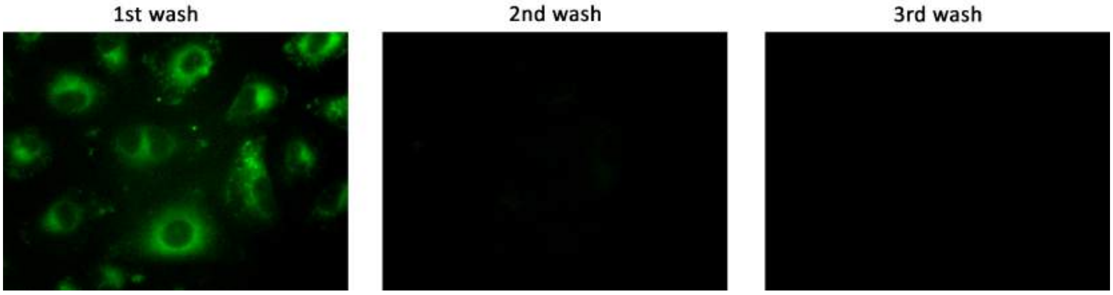

Fig. S106: Fluorescent micrographs from 'washing out' experiment for compound **2**. A549 cells were incubated with **2** (1 µM) for 1 hour, washed twice with PBS and then imaged. Cells were then returned to the incubator for a second hour, washed twice and then imaged again (2nd wash). This process was repeated a third time (3rd wash). Images were corrected for contrast to the brightest image (1st wash) so as to be comparable.

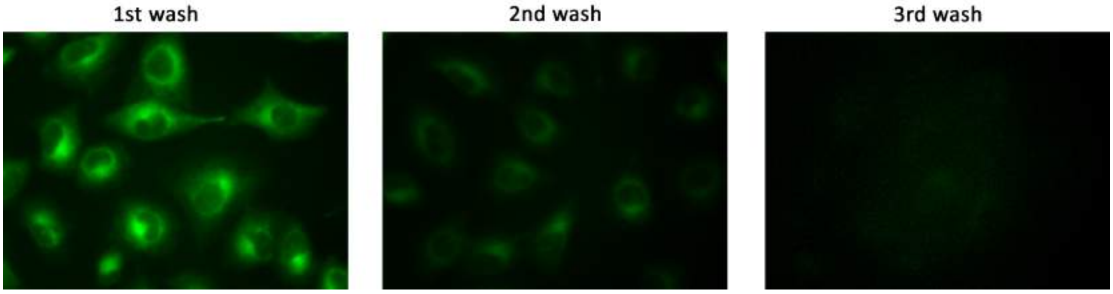

Fig. S107: Fluorescent micrographs from 'washing out' experiment for compound **3**. A549 cells were incubated with **3** (1 μM) for 1 hour, washed twice with PBS and then imaged. Cells were then returned to the incubator for a second hour, washed twice and then imaged again (2nd wash). This process was repeated a third time (3rd wash). Images were corrected for contrast to the brightest image (1st wash) so as to be comparable.

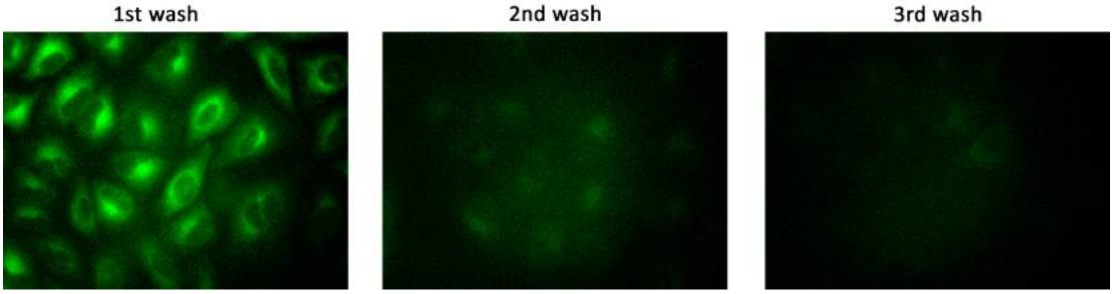

Fig. S108: Fluorescent micrographs from 'washing out' experiment for compound **4**. A549 cells were incubated with **4** (1 μM) for 1 hour, washed twice with PBS and then imaged. Cells were then returned to the incubator for a second hour, washed twice and then imaged again (2nd wash). This process was repeated a third time (3rd wash). Images were corrected for contrast to the brightest image (1st wash) so as to be comparable.

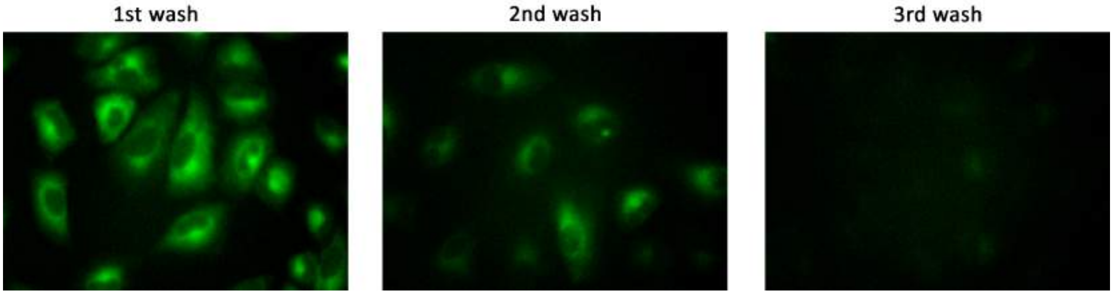

Fig. S109 Fluorescent micrographs from 'washing out' experiment for compound **5**. A549 cells were incubated with **5** (1 μM) for 1 hour, washed twice with PBS and then imaged. Cells were then returned to the incubator for a second hour, washed twice and then imaged again (2nd wash). This process was repeated a third time (3rd wash). Images were corrected for contrast to the brightest image (1st wash) so as to be comparable.

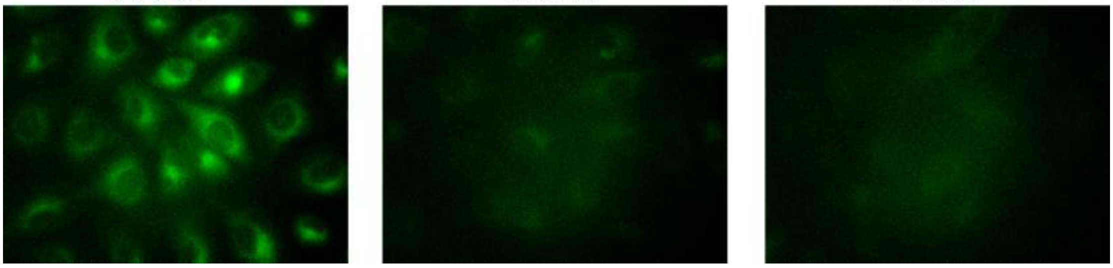

Fig. S110: Fluorescent micrographs from 'washing out' experiment for compound **6**. A549 cells were incubated with **6** (1 µM) for 1 hour, washed twice with PBS and then imaged. Cells were then returned to the incubator for a second hour, washed twice and then imaged again (2nd wash). This process was repeated a third time (3rd wash). Images were corrected for contrast to the brightest image (1st wash) so as to be comparable.

8.3.3 Co-Localisation Experiments

Co-localisation experiments were performed as follows: A549 cells (10^5 cells/mL)/6 well-plate were pre-stained by incubation with with **1** (1 µM) or **2** (5 µM) for 24 hours. Cells were washed twice with PBS before incubation with LysoTracker Red (50 nM) or MitoTracker Deep Red (50 nM) for 1 hour. Subsequently cells were washed twice with PBS and the media replaced and fluorescence micrographs in 20x with the organelle marker observed using the Texas Red filter (LysoTracker) or Cy5 filter (MitoTracker). Pearson's coefficients were calculated using the JACoP plugin for ImageJ.[6]

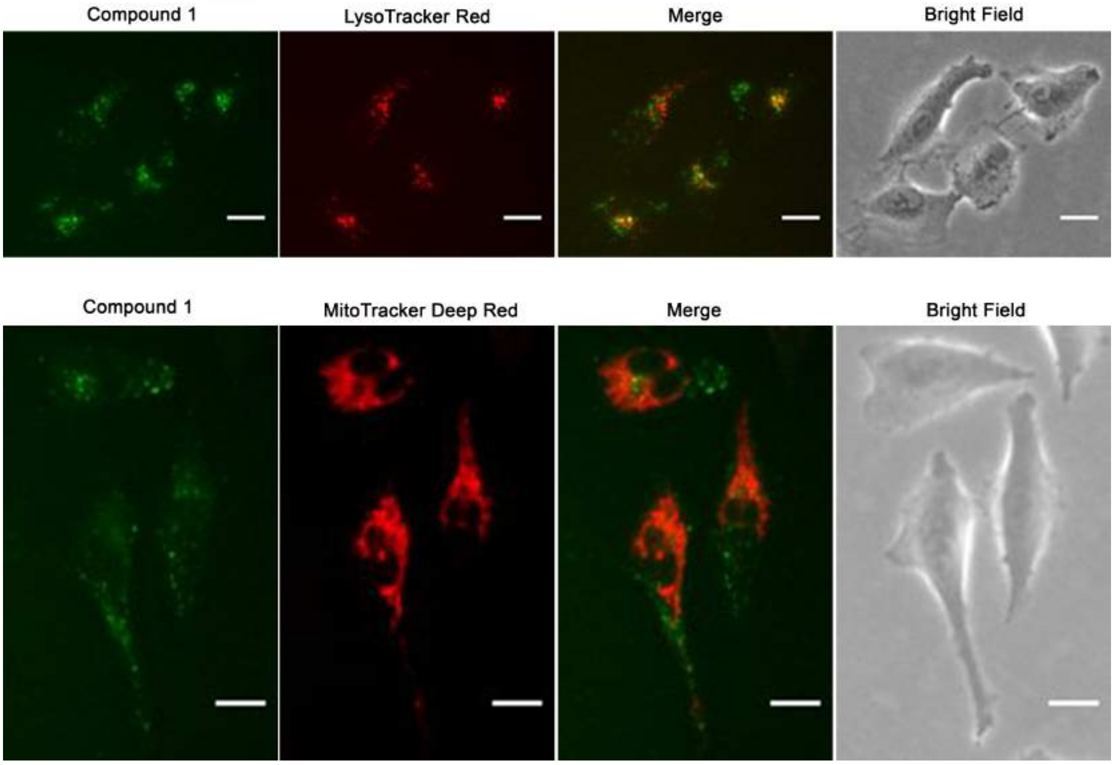

Fig S111: Co-localisation experiment for A549 cells prestained with **1** (1 µM, 24 hrs) and further treated with LysoTracker Red (50 nM, 1 hr) top or MitoTracker Deep Red (50 nM, 1 hr) bottom. Green colour indicated **1**, red indicates cell marker, yellow indicated merged signal. Pearson's coefficients = 0.641 (lysosomes) and 0.449 (mitochondria). Scale bars are 20 µm.

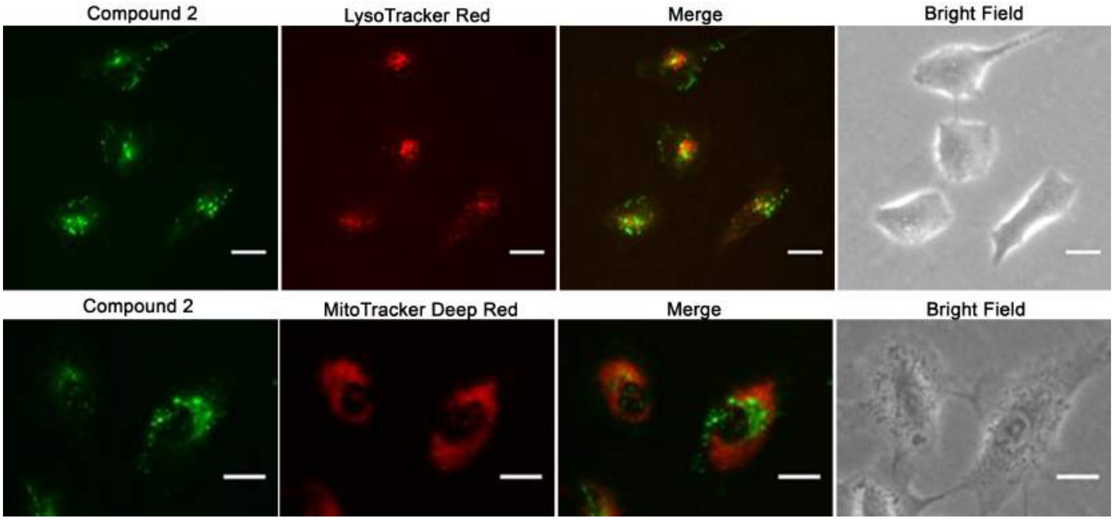

Fig S112: Co-localisation experiment for A549 cells prestained with **2** (5 µM, 24 hrs) and further treated with LysoTracker Red (50 nM, 1 hr) top or MitoTracker Deep Red (50 nM, 1 hr) bottom. Green colour indicated **2**, red indicates cell marker, yellow indicated merged signal. Pearson's coefficients = 0.639 (lysosomes) and 0.496 (mitochondria). Scale bars are 20 µm.

10 – References

1 E. B. Veale and T. Gunnlaugsson, *J. Org. Chem.*, 2008, **73**, 8073–6.

2 T. Minami, Y. Liu, A. Akdeniz, P. Koutnik, N. A. Esipenko, R. Nishiyabu, Y. Kubo

and P. Anzenbacher, *J. Am. Chem. Soc.*, 2014, **136**, 11396–401.
3 P. Thordarson, *Chem. Soc. Rev.*, 2011, **40**, 1305–23.
4 *www.supramolecular.org*. Accessed August 2015.
5 V. Amendola, L. Fabbrizzi, L. Mosca and F.-P. Schmidtchen, *Chem. Eur. J.*, 2011, **17**, 5972–81.
6 S. Bolte and F. P. Cordelières, *J. Microsc.*, 2006, **224**, 213–32.
